# A-to-I editing in human miRNAs is enriched in seed sequence, influenced by sequence contexts and significantly hypoedited in glioblastoma multiforme

**DOI:** 10.1038/s41598-017-02397-6

**Published:** 2017-05-26

**Authors:** Deepanjan Paul, Ashis Narayan Sinha, Arjun Ray, Megha Lal, Subhashree Nayak, Anchal Sharma, Bharati Mehani, Debasish Mukherjee, Saurabh V. Laddha, Ashish Suri, Chitra Sarkar, Arijit Mukhopadhyay

**Affiliations:** 1 Genomics & Molecular Medicine Unit, CSIR-Institute of Genomics & Integrative Biology, Delhi, India; 2 Academy of Scientific and Innovative Research (AcSIR), Delhi, India; 3 Department of Pathology, All India Institute of Medical Sciences, Delhi, India; 4 Department of Neurosurgery, All India Institute of Medical Sciences, Delhi, India; 5 School of Environment and Life Sciences, University of Salford, Salford, United Kingdom

## Abstract

Editing in microRNAs, particularly in seed can significantly alter the choice of their target genes. We show that out of 13 different human tissues, different regions of brain showed higher adenosine to inosine (A-to-I) editing in mature miRNAs. These events were enriched in seed sequence (73.33%), which was not observed for cytosine to uracil (17.86%) editing. More than half of the edited miRNAs showed increased stability, 72.7% of which had ΔΔG values less than −6.0 Kcal/mole and for all of them the edited adenosines mis-paired with cytosines on the pre-miRNA structure. A seed-editing event in hsa-miR-411 (with A – C mismatch) lead to increased expression of the mature form compared to the unedited version in cell culture experiments. Further, small RNA sequencing of GBM patients identified significant miRNA hypoediting which correlated with downregulation of ADAR2 both in metadata and qRT-PCR based validation. Twenty-two significant (11 novel) A-to-I hypoediting events were identified in GBM samples. This study highlights the importance of specific sequence and structural requirements of pre-miRNA for editing along with a suggestive crucial role for ADAR2. Enrichment of A-to-I editing in seed sequence highlights this as an important layer for genomic regulation in health and disease, especially in human brain.

## Introduction

RNA molecules undergo multiple post-transcriptional modifications^1^ for performing diverse functions. Various efforts have been made for identification^2, 3^ and understanding the significance of these modifications^4, 5^. RNA editing – the most well studied modification - changes the information encoded by the genome and adds complexity to the gene regulatory networks^6–8^. The predominant editing event, adenosine-to-inosine (A-to-I) is mediated by ADAR (Adenosine deaminase acting on RNA) family members which acts on double-stranded RNA (dsRNA) with or without a perfect complementarity^9^. With the advent of next generation sequencing multiple groups have devised experimental^10, 11^ as well as computational^12, 13^ approaches to identify genome-wide A-to-I editing events in RNA. For protein-coding transcripts A-to-I editing is essential for normal development^14, 15^ and is enriched in the brain^16^. A-to-I modification happens more promiscuously within perfect dsRNA substrates, deaminating up to 50% of the adenosine residues^17^ whereas internal mismatches and bulges in dsRNA substrates is associated with ADAR selectivity^18^.

Another form of canonical RNA editing event involves cytosine to uracil (C-to-U) deamination^15^ mediated by APOBEC1 (apolipoprotein B mRNA editing enzyme, catalytic polypeptide-like 1). APOBEC1 mediated editing events provide tissue specificity and diversity for ApoB mRNAs^19^ but deregulation of APOBEC1 can also bring about devastating phenotype like cancer^20^.

MicroRNAs (miRNAs) are ~22 nucleotide long, non-coding RNA which usually down regulate gene expression by binding to the 3′-untranslated region (3′-UTR) of mRNAs^21^. Bases 2–8 (seed region) from the 5′-end of the mature miRNA are critical determinants of target complementarity^22^. Premature forms of a miRNA, being a dsRNA molecule, can undergo A-to-I editing at different stages of biogenesis affecting it’s maturation and expression^9, 23^. A recent paper has shown that ADAR1 can bind to miRNAs in its primary, precursor and mature forms, where binding to the primary miRNA was found to be the highest^24^. A-to-I editing in miRNAs can affect its cleavage in the nucleus^25^ or cytoplasm^26^ and might also result in altered target genes. MiRNA editing has been shown to be important in tissue specific regulation in normal brain^27^. A recent study has also shown that A-to-I editing in miRNA increases during development, by analysing different developmental stages of mouse brain^28^.

There is a considerable body of literature for A-to-I editing events in miRNAs^27, 29, 30^. Recently, studies have also started reporting importance of C-to-U editing in miRNAs^31, 32^. However, for both these canonical miRNA editing types, the tissue specific spectrum in normal human tissues remains to be seen. In addition, currently there is no consensus on effect of editing at pri/pre level on processing and expression of mature miRNAs. There are reports that indicate both enhanced^33, 34^ and reduced^25, 26, 35^ processing and expression upon editing.

In this study we have performed a massively parallel sequencing based large-scale analysis for both A-to-I and C-to-U editing on human miRNAs across 13 different tissues. We explored the positional bias of these events and the role of editing in pri-miRNA on mature miRNA expression. Further, editing in different parts of the brain from same individuals were analyzed to look for intra-individual variability and compared with the scenario in brain from patients of glioblastoma multiforme.

## Results

### A-to-I editing in miRNAs are enriched in seed sequence in diverse human tissues

**Figure 1:**
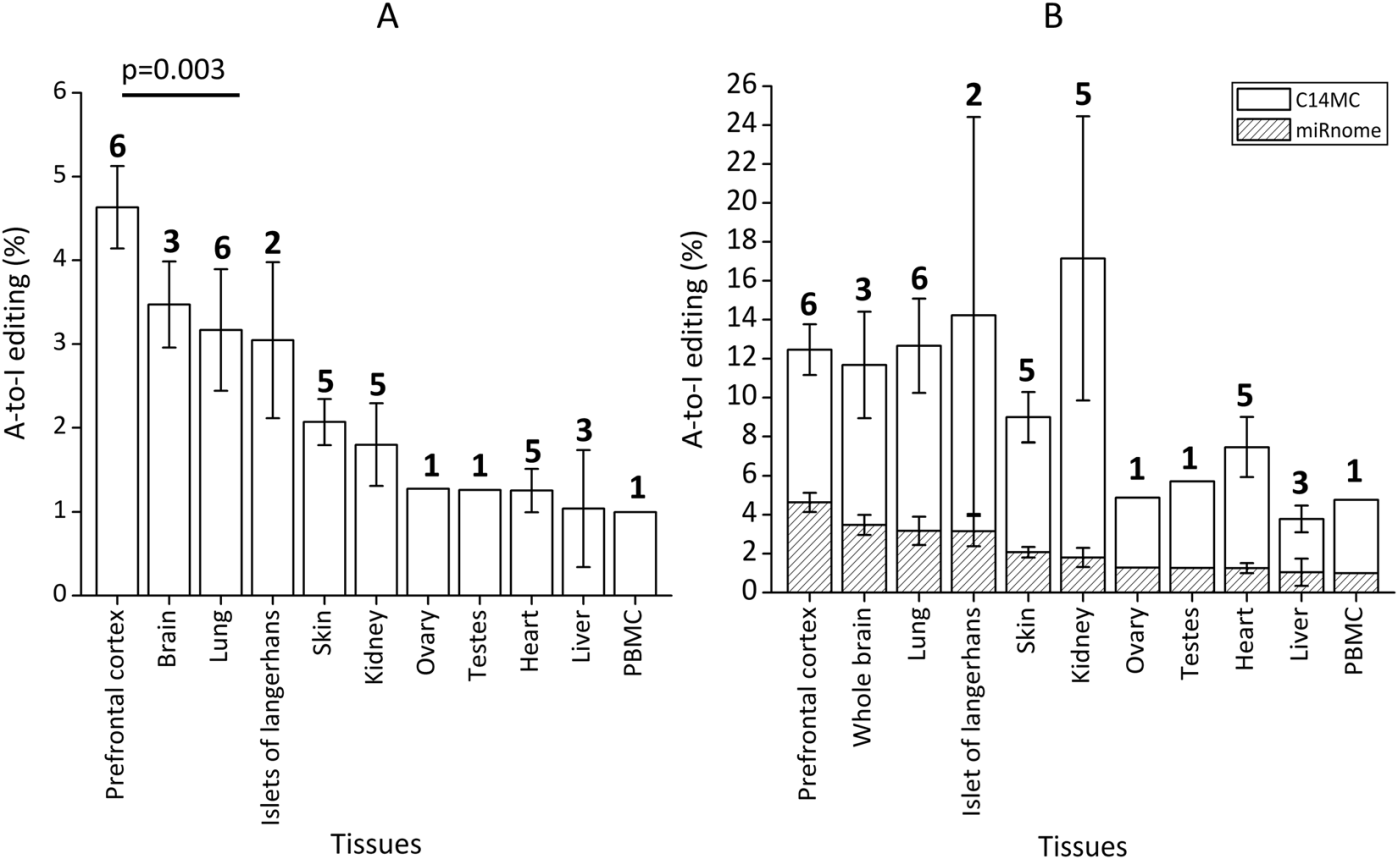
Distribution of A-to-I editing in different healthy human tissues. (**A**) A-to-I editing in mature miRNAs is the highest in Prefrontal cortex (two-tailed t-test; p = 0.003 with respect to lung samples). Percentage A-to-I editing was calculated by dividing the number of edited miRNAs by the total number of miRNAs expressed with a read count greater than equal to 10. The numbers above the bars represent the number of different individuals analysed. (**B**) C14 miRNA cluster show enriched A-to-I editing. The fraction of edited miRNAs from C14 was significantly higher compared to the miRnome average in all tissues analyzed (p < 0.008), the tissues have been arranged according to descending order of miRnome-wide editing.

We have analysed >1 billion sequences from 50 small RNA sequencing experiments representing 13 diverse healthy human tissues (Supplemental Table S1) and identified 60 and 56 non-redundant A-to-I and C-to-U editing events, respectively (Supplemental Table S2). A-to-I editing levels within mature miRNAs were found to be the highest in prefrontal cortex followed by total RNA from brain (Fig. 1A) whereas for C-to-U, liver revealed higher editing (Supplemental Figure S1). Prefrontal cortex harbored 30 non-redundant A-to-I sites (4.63% of the total expressed miRNAs; average of six independent experiments), 11 of which were found in all six samples (Supplemental Figure S2A). Total RNA from brain had 24 A-to-I sites (3.47% of the total expressed miRNAs; average of three independent experiments) out of which eight sites were found in all three samples (Supplemental Figure S2B). Amongst other tissues editing was found to be higher in lung (3.16%; average of six independent experiments; Fig. 1) with 23 non-redundant sites, eight of which were shared in all six samples (Supplemental Figure S2C). Such consistent editing events across multiple samples for other tissues were also found. A detailed list of all A-to-I and C-to-U editing events in all tissues is provided in Supplemental Table S2.

Thirteen out of 60 non-redundant A-to-I editing events (21.66%) were located on chromosome 14. Ten out of these 13 were in miRNAs belonging to the large miR-379/miR-656 cluster alone (henceforth C14MC). For all tissues analysed the fraction of edited miRNAs from C14MC was significantly higher compared to the random chance namely the miRnome average (p < 0.008; Fig. 1B).

**Figure 2:**
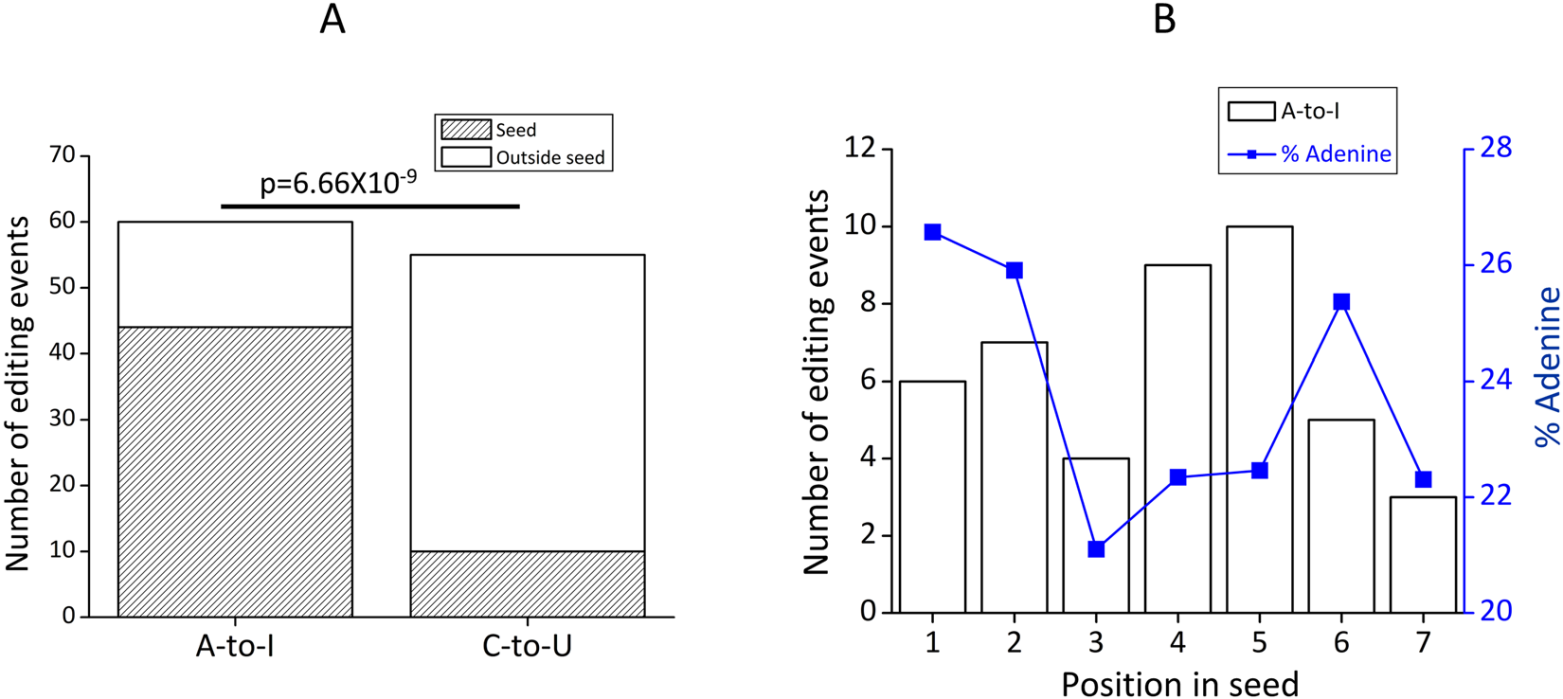
A-to-I editing in mature miRNAs is enriched in seed sequence. (**A**) 73.33% (44/60) of the A-to-I editing events was found to be localized in the seed sequence of mature miRNAs whereas for C-to-U only 17.86% (10/56) were in the seed sequence. This enrichment is significantly (two-tailed proportional test, p = 6.66 × 10^−9^) higher for A-to-I compared to C-to-U. (**B**) Primary y-axis (bars) shows the number of editing events in the seed, the secondary y-axis (line) shows the percentage of adenine in seed sequences of all human mature miRNAs (miRBase20) and the x-axis shows the position within the seed (where position 1–7 corresponds to position 2–8 from the 5′-end of mature miRNAs). 19 out of 44 seed editing was located in the 4^th^ (nine events) and 5^th^ (ten events) positions of the seed (marked by black arrow) without a bias for adenine at that position in human mature miRNAs.

We focused on editing events occurring within the seed sequence of mature miRNAs and found 73.33% (44/60) such sites, whereas such a bias was not observed for C-to-U editing (Fig. 2A; p = 6.66 × 10^−9^). Interestingly, 43% (19/44) of the A-to-I events were located in 4^th^ and 5^th^ positions of the seed without any specific bias for adenosine in those positions (Fig. 2B and Supplemental Figure S3).

### Neuron rich frontal cortex has more A-to-I editing events than corpus callosum of the same individuals

**Figure 3:**
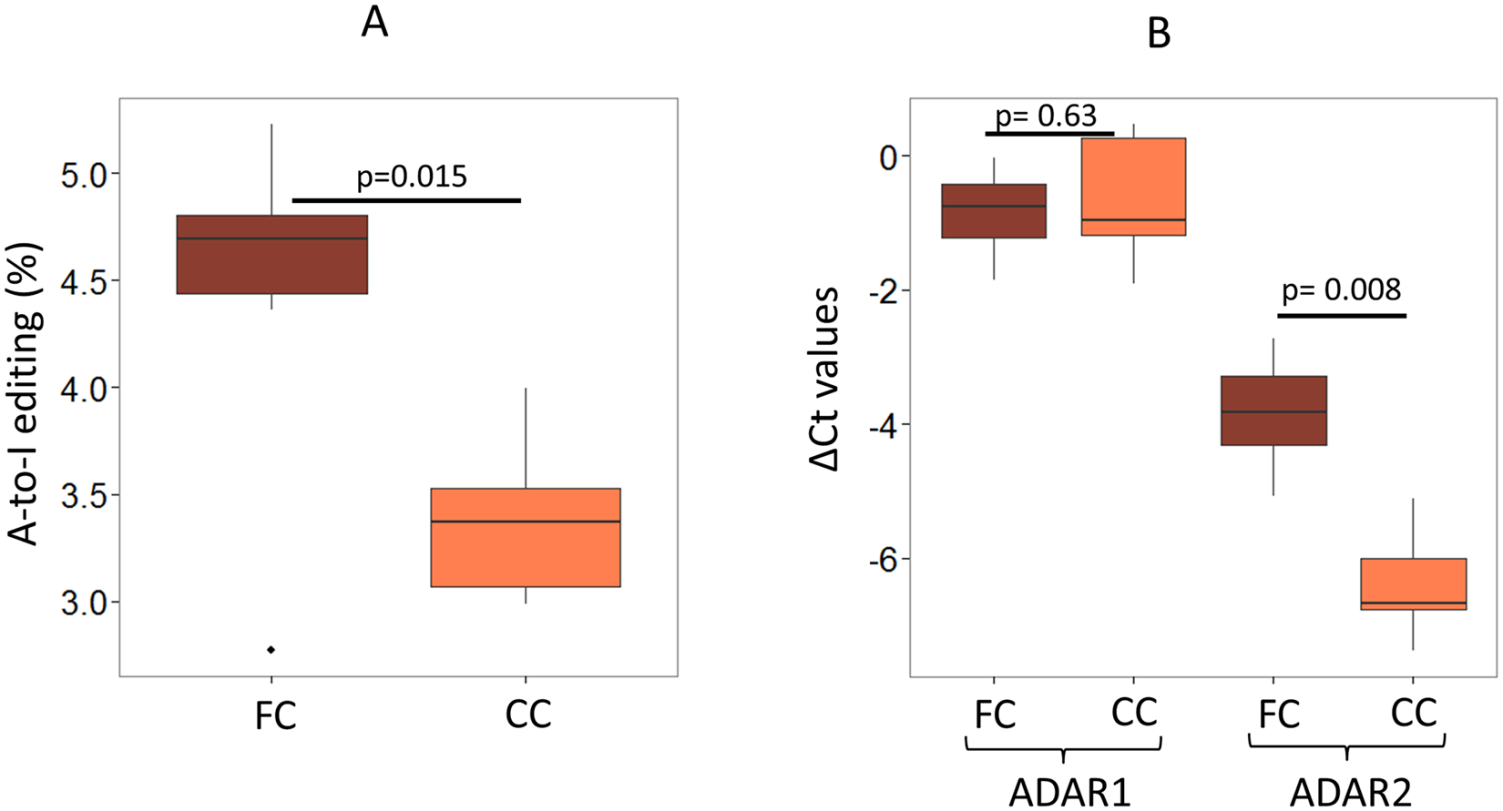
Neuron rich frontal cortex (FC) showed higher A-to-I editing in mature miRNAs than the corresponding corpus callosum (CC) of the same individuals. (**A**) FC showed higher A-to-I editing than CC of the same individuals (two-tailed t-test; p = 0.015). (**B**) Real-time PCR was done and delta Ct was plotted, ADAR2 (and not ADAR1, two-tailed t-test; p = 0.63) showed significant upregulation in FC compared to CC samples (two-tailed t-test; p = 0.008). B2M was used to normalize expression in all samples.

We have performed small RNA sequencing using massively parallel sequencing methods in paired samples of frontal cortex (FC) and corpus callosum (CC) from six individuals. We had exome sequence (DNA) data available for four of these pairs. The exome data was used to rule out the possibility that the A-to-I editing sites were due to variations at the DNA level of the specific individuals. Out of all sites, 75% (33/44) and 70.58% (24/34) were captured by the exome data, in FC and CC, respectively (Supplemental Figure S4A and B). Two observed A > G variations were found independently in FC and CC DNA and hence were excluded from the editing dataset. Over-laying the sites with dbSNP (build 142) identified two SNPs, one in FC (rs515924, A-to-G) and the other (rs554506562) both in FC and CC, which were also excluded. After filtering out these variations at the DNA level, the extent of editing was significantly higher in FC than CC (p = 0.015; Fig. 3A) as well as for all other tissues (p = 0.02 with respect to lung). Our analysis revealed a total of 157 and 122 mature miRNA A-to-I sites in FC and CC, respectively (41 and 32 non-redundant sites in FC and CC, respectively; Supplemental Table S2). As observed for other tissues, in FC and CC also we found an enrichment of editing events within the seed region (70.73% in FC and 75% for CC, Supplemental Table S2). More than one-third of them were present in all six samples for FC and CC. The novel A-to-I miRNA editing events detected in our study is provided in Supplemental Table S3. We did not find significant difference (p = 0.54) in the levels of C-to-U editing between FC and CC samples (Supplemental Figure S4C and D and Supplemental Figure S5).

An event in let-7e showed tissue specificity as well as intra-individual differences in miRNA editing. Hsa-let-7e was expressed in all tissues, while an editing event in its seed sequence was exclusively found in FC (4 out of 6, up to 4.76% edited, Supplemental Table S2) but not in the corpus callosum of the same individuals which has been validated using targeted SnaPshot experiments (Supplemental Figure S6). The edited form potentially targets three genes whereas the unedited form has five targets with none in common (Supplemental Table S4). Apart from these there were five other editing events, which were specific to FC samples and one event specific to CC samples (Supplemental Table S4).

Since ADAR family members are known to mediate the A-to-I editing events, we checked the expression of ADAR1 and ADAR2 in FC and CC by quantitative PCR. Interestingly, for five sample-pairs tested, ADAR2 expression levels were significantly higher in FC than CC (p = 0.008; Fig. 3B) in line with increased editing levels in FC (Fig. 3A). Such a significant expression difference was not observed for ADAR1 (p = 0.63, Fig. 3B).

### A-to-I (and not C-to-U) edited miRNAs are predictably more stable than their unedited forms

**Figure 4:**
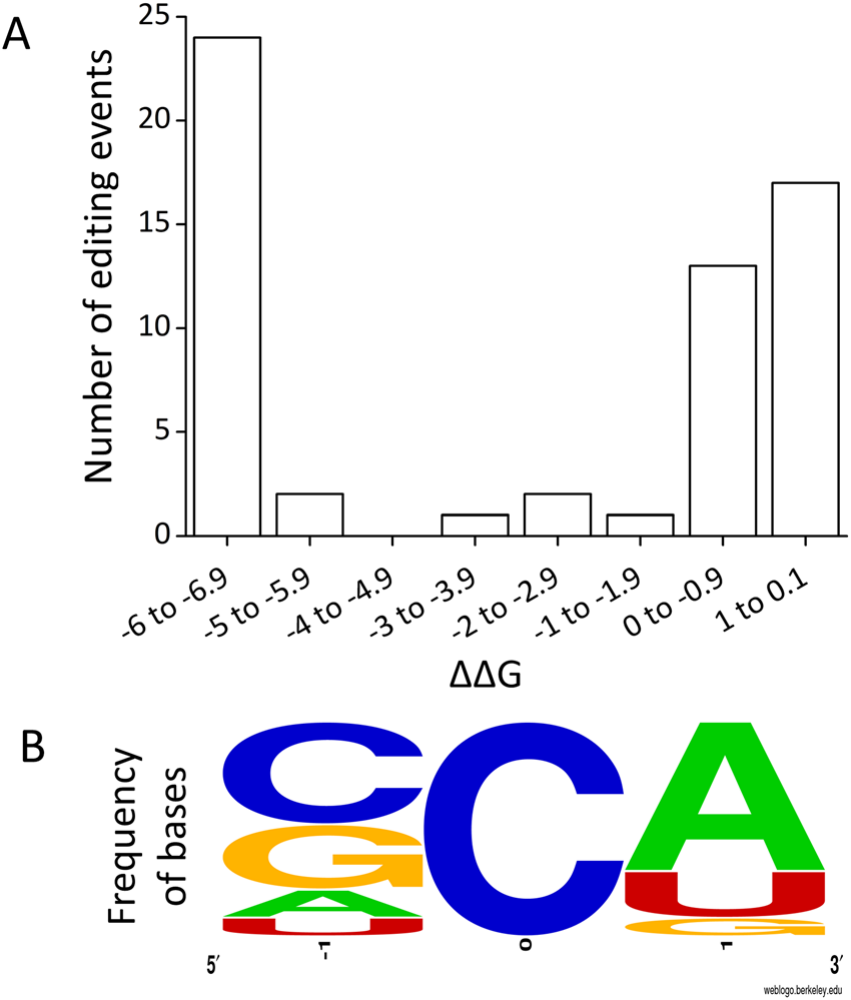
A-to-I edited miRNAs show predicted increased stability than their unedited forms. (**A**) The minimum free energy (MFE) for edited (“G” containing) and unedited (“A” containing) pre-miRNA was obtained using RNA fold. ΔΔG was calculated by subtracting MFE of unedited miRNA from edited miRNA. Negative value of ΔΔG means more stable edited forms. 55% (33/60) sites showed an increase in stability (ΔΔG < zero), 24 out of the 33 sites showed a value less than −6 Kcal/mole. (**B**) The motif for sequence preference in the pre-miRNA fold-back structure is analysed. For all the miRNAs that gained stability upon editing (ΔΔG < −6.0 Kcal/mole) the edited adenosines were found to have mis-paired cytosines on the pre-miRNA hairpin structure.

Using RNA secondary structure analysis we observed that 55% of the edited sites in pre-miRNAs (33/60; Supplemental Table S5A) show an increased stability whereas 16.67% (10/60) show no change in free energy and the rest show a minor decrease in stability (ΔΔG is 0.1 to 0.9 Kcal/mole). 72.7% (24/33) of the edited sites in pre-miRNAs that gain on stability has ΔΔG values less than −6.0 Kcal/mole (Fig. 4A). Only six out of 56 C-to-U edited events (10.71% compared to 55% for A-to-I) were predicted to have increased stability (ΔΔG < 0 kcal/mole) upon editing (Supplementary Figure S7 and Supplementary Table S5B). As ADAR specifically edits double stranded RNA (dsRNA) substrates, we checked the sequence context of the pre-miRNA hairpins. For all the miRNAs that gained stability upon editing (ΔΔG < −6.0 Kcal/mole) the edited adenosines had mis-paired cytosines on the pre-miRNA hairpin structure (Fig. 4B and Supplemental Table S5A). This was not observed for the edited sites that did not provide a stability advantage based on free energy change (Supplemental Table S5A). To validate the altered minimum free energy levels upon editing, we randomly selected 18 miRNAs to check in the three-dimensional structure. Nine out of the 10 candidates that gained in stability in 2D analysis showed the same trend in three-dimensional analysis (Supplemental Table S5A). All these miRNAs had an A-C mismatch in the pre-miRNA hairpin structure pointing towards the importance of this mismatch in conferring stability upon editing.

**Figure 5:**
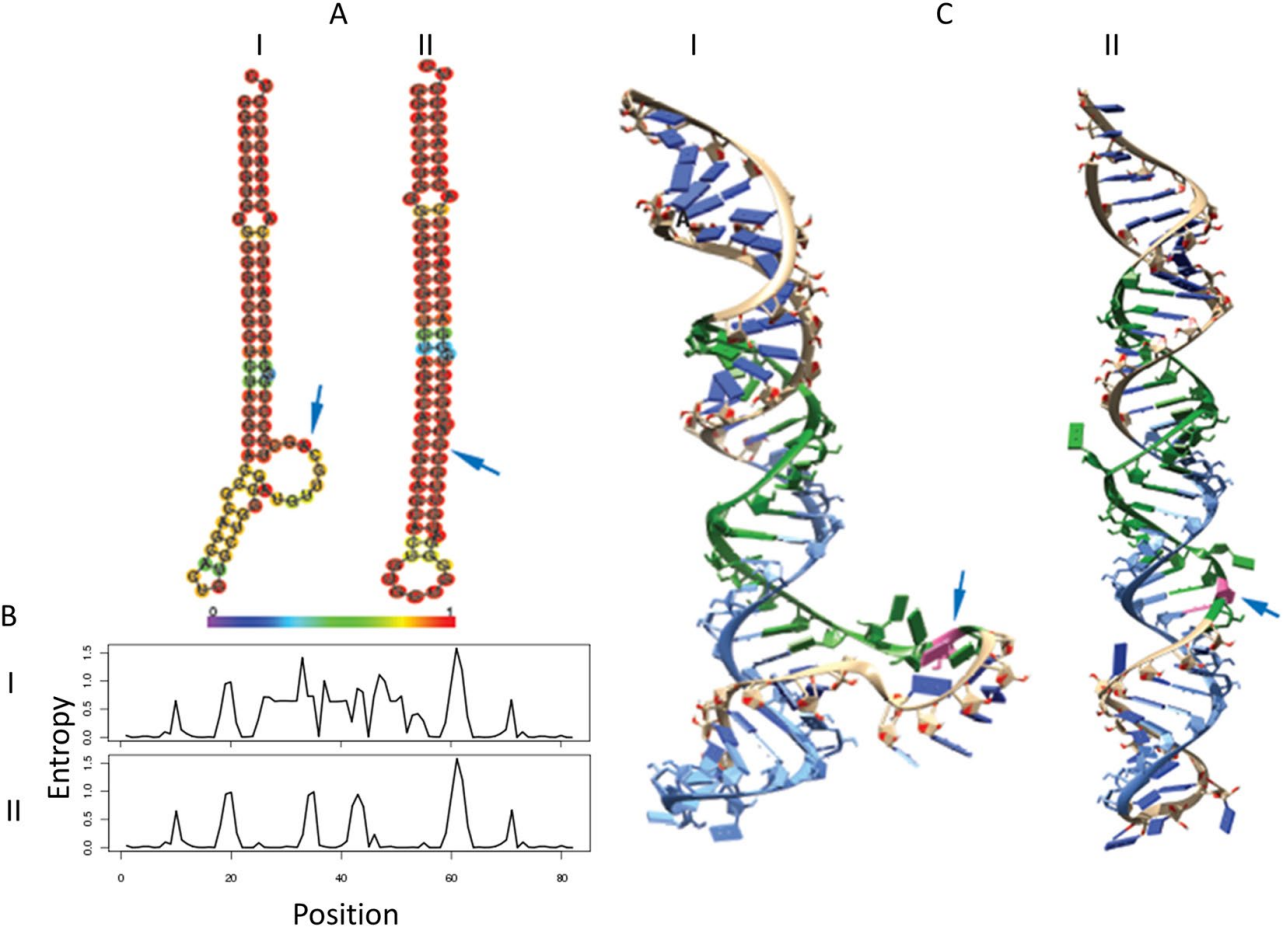
The two- and three-dimensional analysis for a representative pre-miRNA (mir-1301) unedited and edited sequence. (**A**) The minimum free energy secondary structures and its sequences, colored according to the base-pairing probability. The structure was predicted using RNAFold which uses a loop-based energy model and the dynamic programming algorithm. (**B**) The corresponding positional entropy for each position. (**C**) The lowest free energy three-dimensional structure was built using mcfold followed by mcsym where the RNAFold’s secondary structure information was given as structural constrain for unpaired nucleotides. The 5p arm is colored as blue while the 3p arm is colored green, with the edited nucleotide colored as pink. ‘I’ represent the unedited form while ‘II’ represents the edited form. The arrows mark the site of editing. The minimum free energy change at the two- and three dimensional levels for the unedited and edited pre-mirna-1301 is provided in Supplemental Table S5A.

Figure 5 shows the structural features of unedited and edited forms of miR-1301 as a representative example in both two and three dimensional space. The edited species makes 4 hydrogen bonds with guanine while the unedited species has only 1 with adenine (the total hydrogen bonds was 113 and 122, in unedited and edited structures, respectively). The decrease in Gibb’s free energy in 2D is reflected by more compact and ordered structure in 3D (Fig. 5C) with corresponding change in its entropy profile (Fig. 5B).

### ADAR2 downregulation and miRNA hypoediting in Glioblastoma Multiforme

In order to check the expression of ADARs in a disease scenario, we have analyzed data available in the TCGA server for 593 GBM samples and controls. The expression of ADAR2 was significantly downregulated (after bonferroni correction, p = 0.004; Supplemental Figure S8) in GBM. In line with the results described above such significant downregulation was not observed for ADAR1 in the TCGA data (p = 0.2; Supplemental Figure S8).

**Figure 6:**
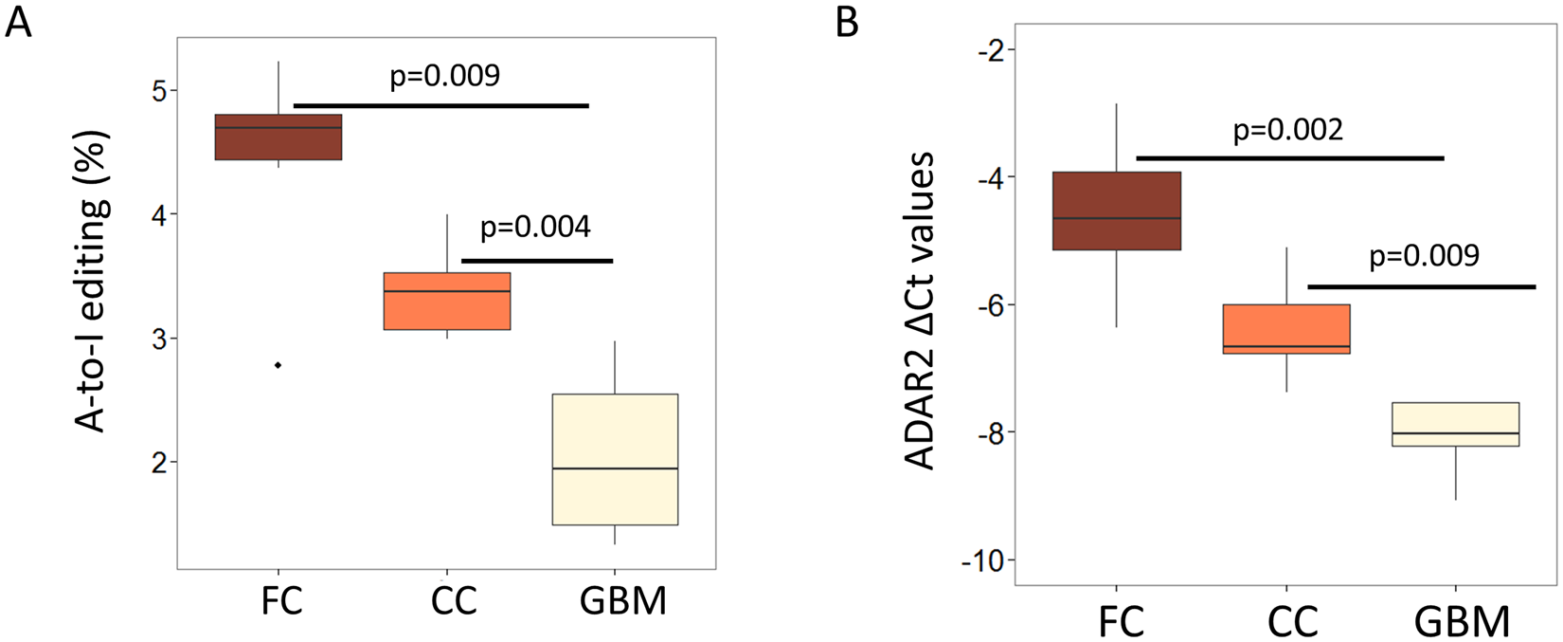
MiRnome wide hypo-editing and ADAR2 downregulation in glioblastoma multiforme. (**A**) Small RNA sequencing revealed miRnome-wide significant (two-tailed Wilcoxon test) hypoediting in GBM compared to FC (p = 0.009) and CC (p = 0.004) samples. (**B**) Real time PCR validation of ADAR2 in GBM samples compared to FC and CC samples. Y-axis shows ΔCt values of ADAR2 in five FC, five CC and five GBM samples and B2M expression was used for normalization. ADAR2 is significantly (two-tailed t-test) downregulated in GBM compared to FC (p = 0.002) and CC (p = 0.009) samples.

**Figure 7:**
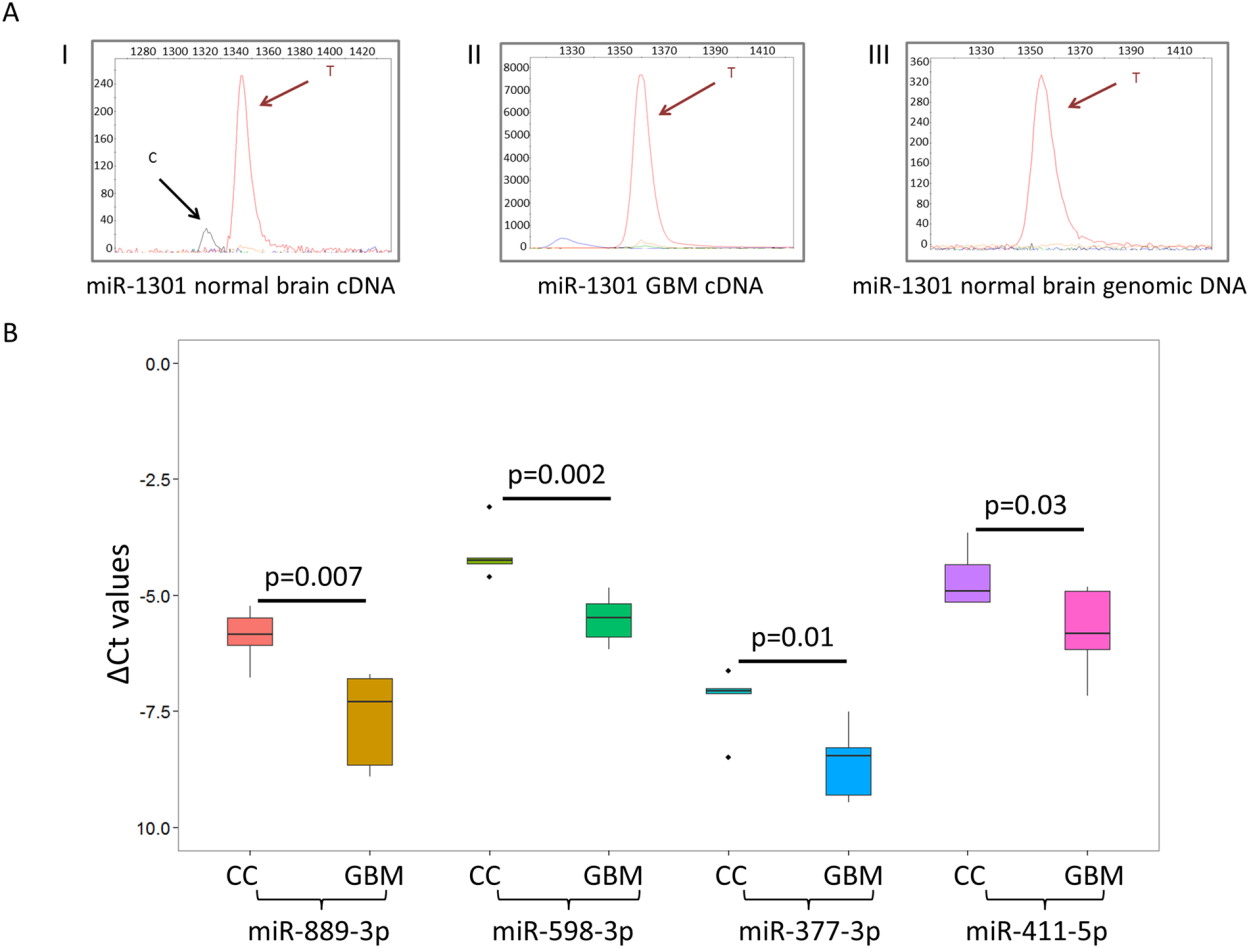
Validation of hypoediting and downregulation of mirna in GBM. (**A**) The X- and Y-axes represents the relative size of the SnaPshot product and relative fluorescence unit (RFU) respectively. One representative example of hypoedited miRNA (mir-1301) in GBM is shown. The editing event observed in normal brain RNA (I) is absent in the GBM tumor RNA (II) and in the normal genomic DNA (III). The SnaPshot specific primer was chosen from the negative strand, hence A to I (G) editing is depicted as T and C peaks. (**B**) Real time PCR validation of downregulation for hypoedited miRNAs in GBM samples compared to CC samples. Y-axis shows ΔCt values of miRNAs in five CC and five GBM samples and U6 snRNA expression was used for normalization. Statistical significance of downregulation was determined by one-tailed t-test. The delta Ct values for these four miRNAs are provided in Supplemental Table S7.

We observed significant hypoediting of mature miRNAs in a set of five GBM samples that we sequenced compared to the six FC and six CC samples used as controls (2.06% in GBM, 4.43% in FC and 3.38% in control; p = 0.009 and p = 0.004, respectively; Fig. 6A). These GBM samples also showed significant downregulation for ADAR2 (p = 0.002 and p = 0.009; Fig. 6B) which most likely have resulted in the observed hypoediting. When checked for individual miRNAs in GBM samples that were edited in FC and CC (total 29 non-redundant events in at least 3 FC and CC samples), 22 (75.86%) showed significant hypoediting (Wilcoxon-two-tailed, p < 0.05) or no editing in GBM. Eleven out of these 22 events were novel miRNA hypoediting events in GBM (Table 1). The hypoediting for three miRNAs was also confirmed by SnaPshot experiments (Fig. 7A and Supplemental Figure S9). Sixteen out of 22 events were within the seed sequence and would result in drastic redirection of targets with the maximum target overlap before and after editing being only 7.53% (Table 1). For eight of these hypoedited miRNAs the editing level was below the level of detection in all GBM samples and amongst others we observed up to 5-fold reduction of the level of editing in GBM (e.g. for hsa-miR-411, Table 1).

**Table 1:** A-to-I hypoedited miRNAs in Glioblastoma Multiforme (GBM).

miRNAs	^a^Presence in samples	^b^Median editing (in %)	Seed editing event	Position in precursor	Target Prediction	^c^Overlap (%)
					Before/After editing	
hsa-mir-598-3p	6/5/0	0.49/0.34/0	Yes	62	11/9	0 (0)
hsa-mir-376a-1-5p	6/6/0	11.24/8.43/0	Yes	9	131/166	4 (3.05)
hsa-mir-337-3p*	4/1/0	4.21/−/0	Yes	66	146/197	11 (7.53)
hsa-mir-376c-3p	6/5/2	3.72/1.9/−	Yes	48	156/192	11 (7.05)
hsa-mir-1301-3p^#,*^	6/6/2	7.59/3.94/−	Yes	52	230/7	2 (0.87)
hsa-mir-421	6/6/3	1.40/0.61/0.57	Yes	54	271/4	1 (0.37)
hsa-mir-99b-3p*	6/6/2	3.61/1.65/−	Yes	47	33/21	0 (0)
hsa-mir-641-5p	6/6/3	5.62/7.08/3.35	Yes	18	355/128	11 (3.1)
hsa-let-7e-3p*	4/0/0	2.09/0/0	Yes	57	5/3	0 (0)
hsa-mir-1251-5p^#,*^	4/4/0	11.98/11.87/0	Yes	10	58/305	4 (6.9)
hsa-mir-381-3p^#^	6/6/5	6.87/7.15/3.07	Yes	52	638/302	48 (7.52)
hsa-mir-411-5p	6/6/5	27.57/30.85/5.71	Yes	20	64/58	0 (0)
hsa-mir-130a-3p	5/5/0	0.76/0.97/0	Yes	56	724/172	27 (3.73)
hsa-mir-151a-3p	6/6/3	2.87/1.26/0.48	Yes	49	76/77	3 (3.95)
hsa-let-7d-3p	6/4/0	0.70/0.31/0	Yes	66	9/1	0 (0)
hsa-mir-27b-3p*	5/0/0	0.25/0/0	Yes	64	921/8	2 (0.22)
hsa-mir-301b-3p*	5/2/1	1.24/−/−	No	63	NA	NA
hsa-mir-340-3p*	4/0/0	1.19/0/0	No	70	NA	NA
hsa-mir-377-3p*	4/3/0	8.10/4.6/0	No	54	NA	NA
hsa-mir-539-5p*	6/6/0	2.23/1.62/0	No	18	NA	NA
hsa-mir-889-3p*	5/5/0	0.62/0.41/0	No	62	NA	NA
hsa-mir-99a-5p	6/6/4	3.40/1.44/0.30	No	13	NA	NA

22 miRNAs were found to be significantly hypoedited in GBM (wilcoxon-two-tailed, p < 0.05).

^a^Presence in samples (FC/CC/GBM).

^b^Median editing (FC/CC/GBM).

^c^Percentage overlap was calculated by overlapped targets/targets before editing.

NA, not applicable because they were not seed editing events.

^“−”^Indicates miRNAs where median value could not be calculated.

The asterisk (*) represents 11 novel miRNAs found to be hypoedited in GBM in this study.

^“#”^Indicates events validated by SNPShot (data in Fig. 7A and Supplemental Figure S9).

**Figure 8:**
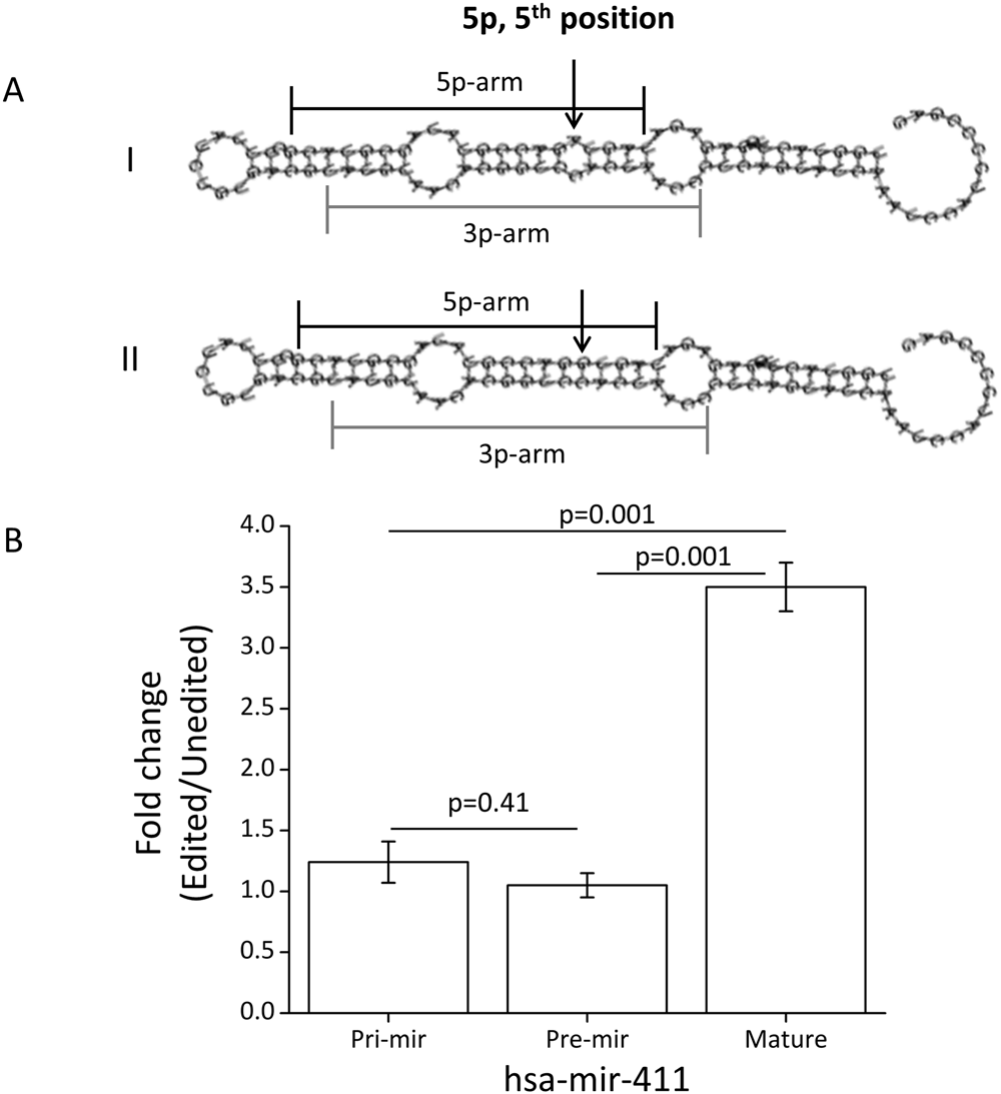
Increased expression of mature edited form of hsa-miR-411. (**A**) The figure shows the predicted secondary structures for the unedited (I) and edited (II) versions of pre-mir-411 using RNAfold Webserver. The arrow shows the position of editing found experimentally in mature miR-411–5p. The edited version (A changed to G) was predicted to have a more stable structure (ΔΔG = −6.6 kcal/mol). (**B**) qRT-PCR data shows altered expression upon editing. A significant (two-tailed t-test, p = 0.001) increase in expression (3.5 fold, average of three replicates) was observed only for the mature form of miR-411. No significant (two-tailed t-test, p = 0.41) change was observed at the level of pri- and pre-mirna.

We investigated differentially expressed miRNAs in GBM compared to FC samples from the miRnome-wide data and identified 263 and 292 up- and downregulated miRNAs, respectively (303 and 268 up- and downregulated miRNAs when compared with CC, Supplemental Table S6A and B). When checked for the altered expression of the hypoedited miRNAs, we found 45.45% (10/22) to be downregulated in GBM (Table 1 and Supplemental Table S6A). Downregulation of four miRNAs found to be hypoedited and downregulated in GBM compared to both FC and CC samples were further validated by qRT-PCR (Fig. 7B and Supplemental Table S7). Seven pre-miRNAs out of these 10 were predicted to have increased structural stability upon editing (ΔΔG values < −6.0 Kcal/mole) and all of them had an A-C mismatch in the pre-miRNA hairpin structure (Supplemental Table S5A). To test the hypothesis that such a gain in stability can positively influence pri-miRNA processing – we have selected miR-411 as a representative miRNA, based on its editing across all tissues studied, an A-C mismatch at the editing site, seed sequence editing and its being one of the miRNAs showing downregulation and hypoediting in GBM. In cell based assays it was found that the expression level of mature miR-411 (edited) was 3.5 fold higher than the un-edited version (Fig. 8). The results were in agreement when compared with the empty vector construct (Supplemental Figure S10).

We found miRnome-wide hypoediting also for C-to-U editing events in GBM compared to FC and CC samples (Supplemental Figure S11) and identified 10 novel hypoediting events (Supplemental Table S8) in GBM.

## Discussion

A-to-I editing in miRNA, or its absence, has been shown to have major consequences for cellular outcomes^27, 36^. We embarked on a large-scale analysis of A-to-I editing in mature miRNAs from small RNA sequencing data across 13 different human tissues. Overall we observed that adenosines flanked by a uracil (5′) and guanine (3′) were more prone to get edited (Supplemental Figure S12), which is in agreement with recent reports^29, 34, 37, 38^. MiRNAs where the edited adenosine was part of the UAG motif showed significantly higher levels of editing than those, which did not have this sequence context (average values 10.21% vs. 3.05%, one-tailed t-test, p = 0.02). Notably, in almost all possible motifs where the adenine residue was preceded by a guanine – it was not edited (Supplemental Figure S12D).

The ADAR mediated deamination requires the substrate to be double stranded^9^. We analysed the base-pairing context of the edited adenosines in the hair-pin structure of the pre-miRNA – which revealed a bias for a mis-pair with cytosine (Fig. 4B). An editing at these sites will create a better base-pairing of cytosine with guanine (inosine), predictably enhancing the stability of the dsRNA – also supported by other studies^29, 39^. This enhanced stability was observed at the level of both 2D and 3D structures of the precursor miRNAs (Figs 4A and 5). Overall, the precursors that gained maximum stability upon editing (ΔΔG ≤ −6.0 Kcal/mole) showed higher levels of editing (average editing 6.94%) compared to those, which did not have any gain in free energy (ΔΔG ≥ 0, average editing 2.55%, one-tailed t-test; p = 0.03). We have also shown for miR-411 in cell based assays that the edited form was significantly more expressed than the unedited version (Fig. 8 and Supplemental Figure S10). Recently, similar observations have been made where edited miR-497* (also with A-C mismatch) showed increased processing by Drosha and was found to be the most downregulated in ADAR2 deficient mice^33^. It is reported that for a pre-miRNA secondary structure a perfect hairpin might be a barrier for efficient maturation process and/or a promiscuous editing^17, 40^. However, our study along with others^33^ indicates that a local mismatch (A-C), which can be removed through editing (I/G-to-C), can actually lead to a better processing for specific miRNAs. The functional link between ADAR group of enzymes and editing vis-à-vis processing of miRNA needs to be studied in greater depth. A recent paper has shown ADAR2 overexpression alters expression of large number of miRNAs^35^. The miRNAs showing reduced expression were mostly onco-miRs, where an editing event can be protective by negatively affecting processing to mature miRNAs^35^. Similarly, it will be important to identify tumor suppressor miRNAs where an editing event can lead to a natural protection by positively affecting the miRNA maturation. Interestingly, in our study, miR-411 is a potential tumor suppressor miRNA^41^ where an editing event leads to increased processing – indicating such possibilities in a larger scale.

ADAR family members, especially ADAR1 and ADAR2 have been shown to have both overlapping and unique editing targets for mRNAs^42^ and miRNAs^27, 42, 43^. It has been recently shown that ADAR2 expression correlated more with the mRNA editing levels in cerebellum and prefrontal cortex than it did with ADAR1^44^ – which is also supported in case of miRNA by others^29, 33^ and by this study. We observed significantly higher expression of ADAR2 in FC compared to the corresponding CC samples (not observed for ADAR1), hinting that one of the many possible reasons for higher editing in FC can be due to higher expression of ADAR2. It remains to be seen whether functional requirement of higher editing levels in post-mitotic cells of the adult brain (e.g. neurons) induces increased expression of the editing enzyme.

We have studied glioblastoma multiforme (GBM) as a representative disease of the brain to understand the possible dysregulation of miRNA editing in disease. Small RNA sequencing of five GBM samples revealed overall hypoediting of miRNA compared to six FC and six CC samples and identified 22 (11 novel) hypoediting events in GBM. Interestingly, eight of these 22 hypoedited miRNAs belonged to the miR-379/miR-656 cluster. It has been suggested that miRNAs in C14MC results from a large polycistron^45^ and it possibly functions as a single tumor suppressor gene^41^. Whether an increased editing adds to this cluster’s functional diversity will be revealed by further studies. We also observed downregulation of ADAR2 in GBM samples both in meta-data (TCGA dataset, Supplemental Figure S8) and qRT-PCR based validation (Fig. 7B) in our samples.

Through this study we have been able to show that in both healthy and diseased state, miRNA editing is an important layer of information with specific sequence and structural preferences – especially in the human brain. Such varied layers of regulatory parameters in the biological systems makes them inherently complex and intricately interconnected – achieving remarkable plasticity that is required to dynamically evolve in situations hitherto unknown.

## Materials and Methods

### Samples

Portions of Frontal cortex (Grey matter) and Corpus Callosum (White matter) were obtained from post-mortem samples of road accident victims. The samples were obtained from NIMHANS Brain Bank, Bangalore, India. GBM samples were obtained from AIIMS, New Delhi, India. The samples were collected according to the Helsinki Declaration and the ethical review board of All India Institute of Medical Sciences, Delhi, India approved the project. Sample collection, characterization and storage were done as described previously^41^. Diagnosis and grading of tumor samples were done as per 2007 WHO classification.

The details of the samples used in the study are provided in Supplemental Table S1.

### RNA isolation, library preparation and sequencing

Total RNA was isolated from Frontal Cortex, Corpus Callosum and GBM samples using miRvana miRNA isolation kit (Ambion, USA) as per the manufacturer’s instructions. Libraries were prepared using Illumina’s TruSeq Small RNA Sample prep Kit following the manufacture’s protocol. Cluster generation and sequencing was done on Illumina HiSeq2000 using standard Illumina sequencing workflow with the multiplexing option. Two samples were loaded in one lane and 50 bases single-end sequencing was done. The in-house small RNA sequencing data is deposited at the sequence read archive (SRA ID: SRP063390).

### Data analysis

Data analysis was done using the published pipeline^29^ with the default parameters. Briefly, the 3′ adapters were removed from the sequencing reads. Reads shorter than 15 bases and longer than 28 bases were discarded. The trimmed reads were aligned against the genome using Bowtie 0.12.7 allowing 1 mismatch. Reads were mapped to pre-miRNA sequences (from miRBase 20) and binomial statistics was used to filter-out sequencing errors. Only significant (p-value < 0.05, post Bonferroni correction) modifications were considered. miRNAs with a minimum read-count 10 was considered. The software and detail description is available at <tau.ac.il/~elieis/miR_editing>.

dbSNP build 142 and exome sequencing data was used to filter out variations at the DNA level.

Percentage of A-to-I editing was calculated in the following way:

Percentage A-to-I editing = (Number of edited miRNAs/Total miRNAs expressed with greater than equal to 10 read counts) * 100. The same was followed for C-to-U editing.

### Publicly available small RNA sequencing data

The detailed list of the public domain sequencing data is provided in Supplemental Table S1. The same pipeline was used for both in-house and publicly available dataset for calling miRNA editing events.

### DNA isolation and Exome Sequencing

DNA was isolated from brain tissues using Omniprep Genomic DNA isolation kit (G-Biosciences, USA) as per manufacturer’s protocol. Exome capture was done using Illumina TruSeq Exome capture kit. 100 base pair paired end sequencing was done using Illumina HiSeq 2000 (Ilumina, USA). The exome sequence data is publicly available at the sequence read archive (SRA ID: SRP045655).

### Exome sequencing data analysis

Raw data was checked for per base quality score and reads having 80% bases with phred quality score 30 and greater were carried forward for downstream analysis and rest were discarded.

#### Alignment

Reads were aligned to the reference genome (hg19) using BWA (version 0.6.1)^46^ allowing for 2 mismatches. More than 98% percent of the data was aligned to reference for each sample. Data was also checked for PCR duplicates and the same were removed.

#### Variation Calling

Genome Analysis Toolkit version 1.5^47^ along with Samtools (version 0.1.18)^48^ was used to call variations from all the paired samples (CC vs. FC). Variants with >90% strand bias were removed, minimum base quality score was kept at 20 and minimum mapping quality of a read was kept at 40.

### Target prediction

The target prediction for unedited and edited miRNAs was done using TargetScanHuman 5.2 Custom (http://www.targetscan.org/vert_50/seedmatch.html) with the 7-mer seed sequence from 2 to 8 nucleotides as an input^49^.

### Motif analysis

Ten bases up-stream and down-stream of the edited adenosine was taken for the motif analysis using WebLogo (http://weblogo.berkeley.edu/logo.cgi)^50^ and the frequency of bases at each position was calculated. The motif analysis was done for all the 60 miRNAs found to be edited in our study.

To look for tri-nucleotide sequence enrichment within pre-miRNAs, 1872 human pre-miRNAs was downloaded from miRBase v20. Three bases sliding window was taken to analyse enriched tri-nucleotides within pre-miRNAs using an in-house perl program.

### Two dimensional (2D) and three dimensional (3D) structural analyses

Minimum free energy (MFE; ΔG) for the unedited and edited miRNAs was predicted at the 2D and 3D level. For secondary structure analysis RNAfold WebServer (http://rna.tbi.univie.ac.at/cgi-bin/RNAfold.cgi)^51^ was used to calculate the partition function and base pairing probability matrix in addition to the MFE structure. MFE is calculated using a loop-based energy model (using Turner model for Energy Parameters) and McCaskill’s algorithm^52^ for the secondary structures contributing towards the minimum free energy in the RNA by summing the contributing free energies from the loops at 37 °C. The unedited and the edited pre-miRNAs were given as inputs for ΔG prediction. ΔΔG was calculated by subtracting ΔG of unedited from ΔG of edited pre-miRNAs.

For 3D structural analysis, 18 candidate miRNAs were chosen (Supplemental Table S5A) and the optimal secondary structure in dot-bracket notation (from RNAfold WebServer) was used as structural constrains in the MC-Fold | MC-Sym pipeline^53^. Structural constrains forces certain nucleotides to be either paired or unpaired and will restrict the conformational search space. The advantage of using a dual approach is that it shall use the best secondary model from the first method and feed it as a template to guide and further predict the new secondary structure using the mcfold algorithm. The final tertiary structure of the pre-miRNA was predicted using mc-fold mc-sym pipeline. The energy-minimized model was obtained using the method’s scoring function, which calculates the base pairing energy contribution by reducing the nucleotides into cyclic motifs ΔΔG of the tertiary structure was calculated in the same way as that of the secondary structure.

### Expression analysis of ADAR1 and ADAR2

1 micro-gram of RNA was converted into cDNA using High-Capacity cDNA Reverse transcription kit (Thermo Fischer Scientific) as per manufacturer’s protocol in a reaction volume of 20 μl. qRT-PCR (in duplicates) of ADAR1 and ADAR2 was done in five pairs of FC and CC samples. qRT-PCR was not performed in one pair due to limited amount of sample. The expression level of B2M was used for normalization. Statistical significance was calculated on the basis of two-tailed paired t-test.

We have downloaded the mRNA expression data of 593 GBM patients and 10 controls (Agilent G4502A array) from TCGA data portal and analyzed expression, which was lowess normalized data (level3). Differential expression of mRNA between patients and controls was determined using a non-parametric two-tailed wilcoxon test. A Bonferroni correction was done to find out the list of significant differentially expressed mRNAs (p < 0.05, post-correction). The significant (two-tailed paired t-test) down regulation of ADAR2 was validated in five GBM samples compared to five FC and five CC samples by qRT-PCR.

### Differential miRNA editing and expression in GBM

Small RNA sequencing was done for five GBM samples. The status of miRNAs found to be edited in at least three FC and CC samples (29 non-redundant events) were compared with the GBM samples and differentially edited miRNAs were identified using two-tailed Wilcoxon test (p < 0.05).

DESeq2 package in R^54^ was used to identify differential expressed miRNAs in GBM compared to FC and CC samples. Two-step filtering was done to reduce false positives; an adjusted p-value (p < 0.1) cut-off followed by a fold change value of ±1.5 was used for further analysis. The data for DESeq2 (Supplemental Table S6A and B) represents the fold change in log_2_ scale (≥0.60 for upregulation and ≤−0.60 for downregulation).

### Hypoedited and downregulated miRNAs in GBM

Hypoedited miRNAs were validated using SnaPshot reaction. Downregulation of miRNAs were validated using qRT-PCR. Briefly 0.4 micro-gram of RNA was converted into cDNA using QuantiMir kit (System Biosciences, USA) as per manufacturer’s protocol in a reaction volume of 10 μl. qRT-PCR (in duplicates) of hypoedited miRNAs were done in five GBM and five CC samples. U6 snRNA expression level was used for normalization.

### Overexpression in HEK293T cell line

A genomic region encompassing mir-411 was cloned in a modified pRIP vector (673 base-pairs). Site-directed mutagenesis was performed using QuikChange Site-Directed Mutagenesis Kit (Agilent) following manufacturer’s protocol. The unedited and edited constructs were separately transfected for overexpression in HEK293T cell line in three biological replicates. Cells were harvested 48 hours post-transfection and mirVana miRNA Isolation Kit (Thermo Fisher Scientific) was used to isolate total RNA. 1 micro-gram of total RNA was treated with DNase I, RNase free (Thermo Fisher Scientific) to get rid of genomic DNA contamination. DNase I treated RNA was used to make cDNA using High-Capacity cDNA Reverse Transcription Kit (Thermo Fisher Scientific) and QuantiMir Kit (System Biosciences) following manufacturer’s protocol. qRT-PCR was done to quantify the levels of Pri-, Pre- and mature mir-411. Relative fold change of edited miR-411 over unedited was calculated using 2^−ΔΔCt^ method.

## Electronic supplementary material

Supplemental data
