# Supplementary material for: A-to-I editing in human miRNAs is enriched in seed sequence, influenced by sequence contexts and significantly hypoedited in glioblastoma multiforme
Source: Sci Rep. 2017 May 26;7:2466. doi: 10.1038/s41598-017-02397-6 (PMC5446428; doi:10.1038/s41598-017-02397-6)
Supplement: Supplementary file 1 — Supplemental data [file 41598_2017_2397_MOESM1_ESM.pdf]

A-to-I editing in human miRNAs is enriched in seed sequence, influenced by sequence contexts and significantly hypoedited in glioblastoma multiforme

Deepanjan Paul[1,2], Ashis Narayan Sinha[1], Arjun Ray[1,2], Megha Lal[1,2], Subhashree Nayak[3], Anchal Sharma[1,2], Bharati Mehani[1,2], Debasish Mukherjee[1], Saurabh V. Laddha[1], Ashish Suri[4], Chitra Sarkar[3], Arijit Mukhopadhyay[1,2,5*]

[1]Genomics & Molecular Medicine Unit, CSIR-Institute of Genomics & Integrative Biology, Delhi, India

[2]Academy of Scientific and Innovative Research (AcSIR), Delhi, India

[3]Department of Pathology, All India Institute of Medical Sciences, Delhi, India

[4]Department of Neurosurgery, All India Institute of Medical Sciences, Delhi, India

[5]School of Environment and Life Sciences, University of Salford, United Kingdom

Short Title: MiRNA editing in human brain

Key words: MiRNA, editing, expression, brain, Glioblastoma Multiforme (GBM)

*Corresponding author: Arijit Mukhopadhyay (a.mukhopadhyay@salford.ac.uk or arijit.mukhopadhyay@gmail.com)

Supplemental data: This document contains all the supplemental data (figures and tables). The supplemental figures are provided first followed by supplemental tables in a sequential manner in which they are mentioned in the manuscript.

Supplemental Figure S1

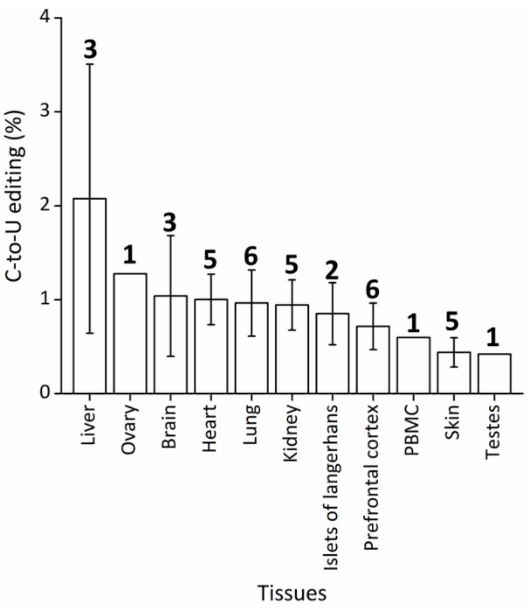

S1: **Distribution of C-to-U editing in different healthy human tissues**. The percentage miRNA editing was calculated by dividing the number of edited miRNAs by the total number of miRNAs expressed with a read count greater than equal to 10. Liver showed the maximum number of editing in mature miRNAs, with testes showing the least amount of C-to-U editing. The numbers above the bars represent the number of different individuals analysed.

Supplemental Figure S2

(A) (B) (C)

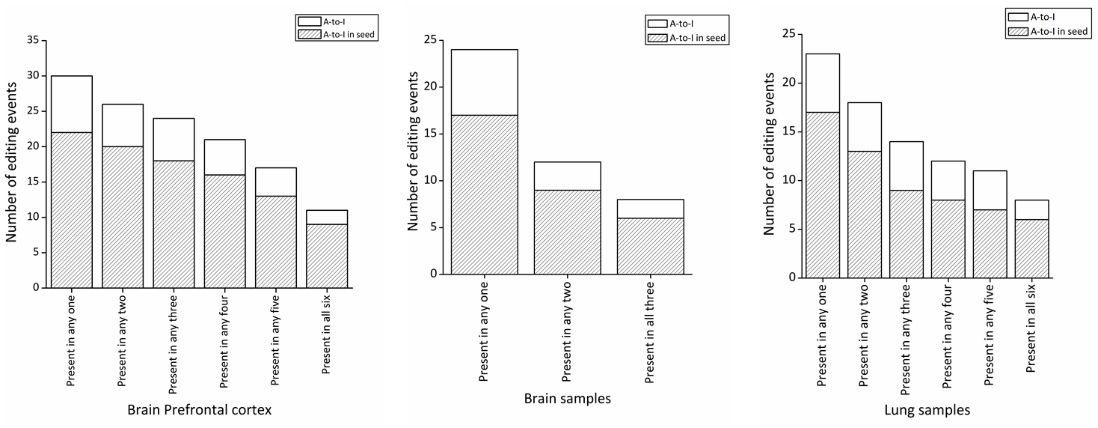

S2: **Recurrence of editing and seed editing events across samples increases the confidence of called sites**. (A). 11 (nine in seed) A-to-I editing events out of 30 non-redundant (22 in seed) are present in all six prefrontal cortex samples. (B). Eight (six in seed) A-to-I editing events out of 24 non-redundant (18 in seed) events are present in all three brain samples (C). Eight (six in seed) editing events out of 23 non-redundant (17 in seed) events are present in all six lung samples.

Supplemental Figure S3

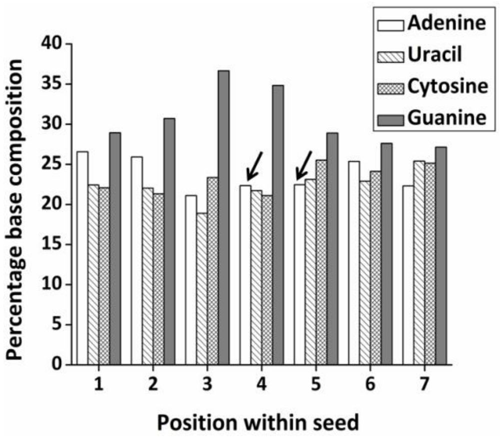

S3: **Distribution of bases in the seed sequence of mature miRNAs**. Percentage base composition for 2578 mature human miRNAs (miRBase 20) do not show enrichment of adenine at the 4th and 5th (marked by black arrow) position of the seed.

Supplemental Figure S4

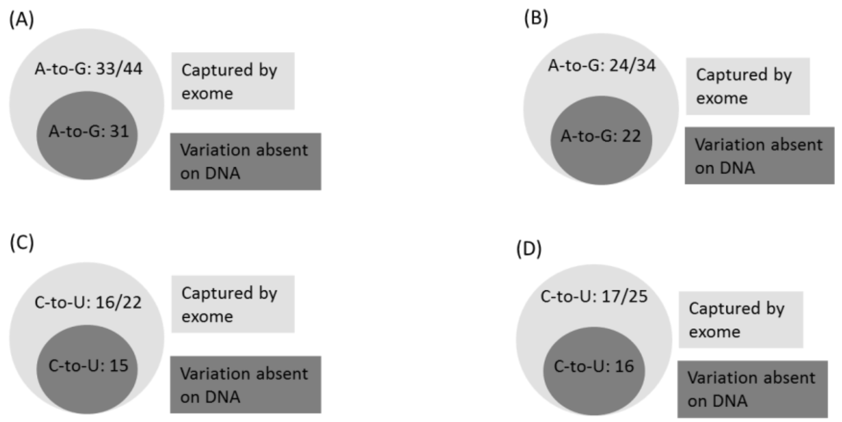

S4: Exome sequencing was used to rule out variation at the DNA level. For A-to-G, 75% (33/44) and 70.58% (24/34) of the modifications were captured by exome sequencing in FC (A) and CC (B), respectively. 31 out of 33 and 22 out of 24 did not show variation at the DNA level.

For C-to-U, 72.72% (16/22) and 68% (17/25) of the modifications were captured by exome sequencing in FC (C) and CC (D), respectively. 15 out of 16 and 16 out of 17 did not show variation at the DNA level.

Supplemental Figure S5

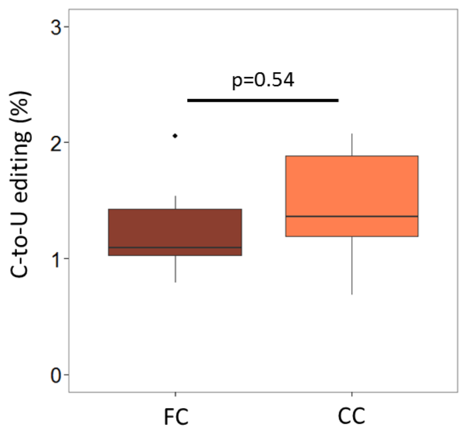

S5: **C-to-U editing in FC and CC samples**. FC and CC samples did not show any significant (two-tailed t-test, p=0.54) difference in the extent of C-to-U editing in mature miRNAs.

Supplemental Figure S6

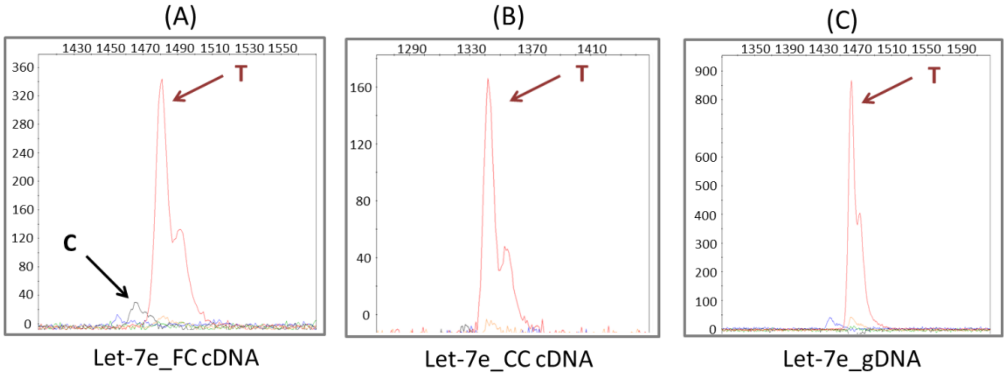

S6: SnaPshot valdiation of Let-7e seed A to I (G) editing event. The X- and Y-axes represents the relative size of the SnaPshot product and relative fluorescence unit (RFU), respectively. An editing event (marked by C) is observed specific to frontal cortex RNA (panel A) which was absent in the corpus callosum RNA (panel B) and the genomic DNA of the same individual (panel C). The SnaPshot specific primer was chosen from the negative strand, hence A to I (G) editing is depicted as T and C peaks. The low peak intensity of the edited site in panel A is commensurate with the low level of editing detected in FC RNA (upto 4.76%).

Supplemental Figure S7

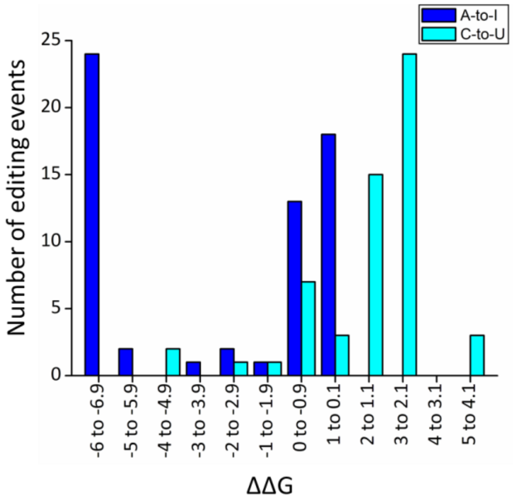

S7: Two-dimensional RNA structure prediction for A-to-I and C-to-U edited miRNAs. 55% (33/60) A-to-I sites on pre-mirna showed an increase in stability (ΔΔG< zero) compared to 10.71% (6/56) for C-to-U sites.

Supplemental Figure S8

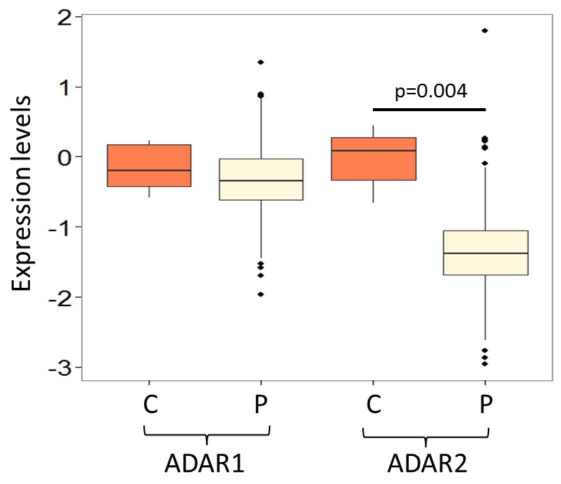

S8: The Cancer Genome Atlas (TCGA) data showing ADAR1 and ADAR2 expression in GBM compared to controls. Analysis of 593 GBM patients (represented as "P") and 10 controls (represented as "C") showed significant (two-tailed wilcoxon test, followed by Bonferroni correction) down regulation for ADAR2 (p=0.004) and not ADAR1 (p=0.2).

Supplemental Figure S9

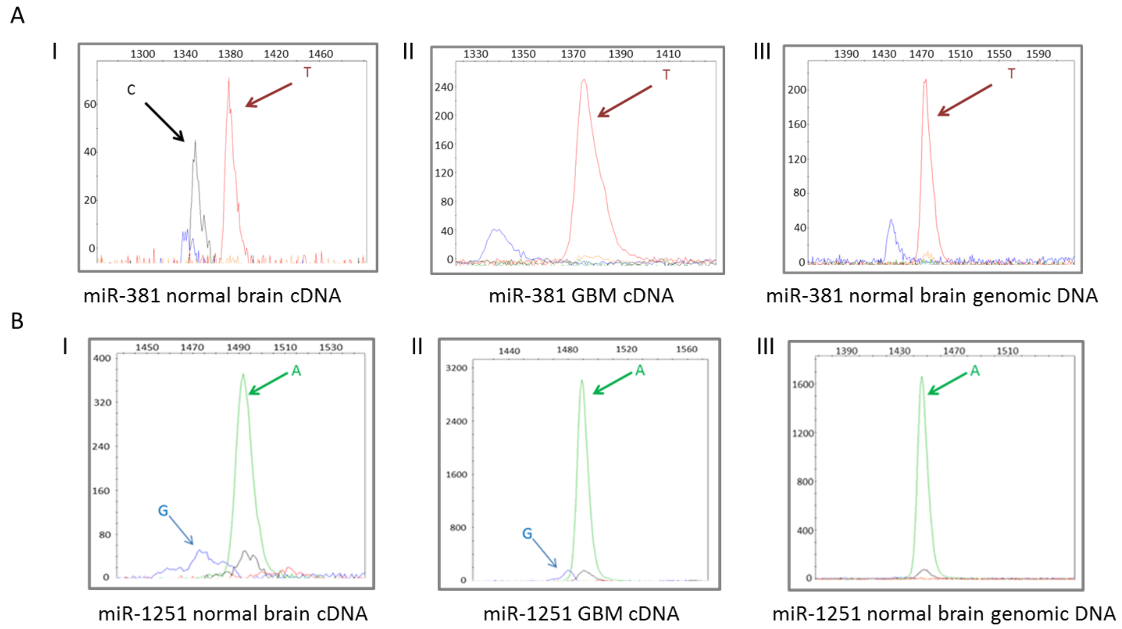

S9: **SnaPshot valdiation of hypoediting in GBM**. The X- and Y-axes represents the relative size of the SnaPshot product and relative fluorescence unit (RFU) respectively. Validation of two hypo-editing events is shown here for miR-381 (panel A) and miR-1251 (Panel B). In both cases the editing event observed in normal brain RNA (I) is either absent or decreased in the GBM tumor RNA (II) and absent in the normal genomic DNA (III). The SnaPshot specific primer for miR-381 was chosen from the negative strand, hence A to I (G) editing is depicted as T and C peaks. For miR-1251, forward primer was used and depicted as A and G peaks.

Supplemental Figure S10

A

	Pri-mir-411			Pre-mir-411			hsa-miR-411		
	Empty vector	Unedited	Edited	Empty vector	Unedited	Edited	Empty vector	Unedited	Edited
Rep1	8.26	-3.99	-4.59	9.94	-3.77	-4.06	6.89	2.48	0.53
Rep2	7.48	-4.89	-5.23	9.59	-4.64	-4.72	7.13	1.58	-0.09
Rep3	6.45	-4.86	-4.75	8.06	-4.54	-4.33	6.79	1.92	0.13

B

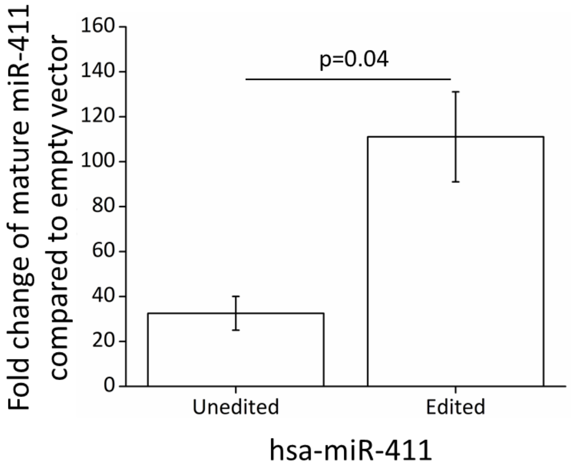

S10: **Mir-411 over expression in HEK293T cells.** (A) The delta Ct values of empty vector, unedited and edited mir-411 construct for Pri-, Pre- and mature mir-411. (B) Edited mir-411 in the mature form is significantly (two-tailed t-test on fold change, p=0.04) over expressed than its unedited version when compared with empty vector control.

Supplemental Figure S11

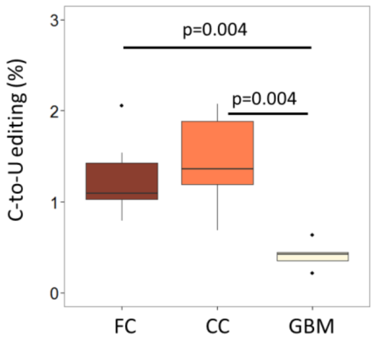

S11: C-to-U editing in mature miRNAs in FC, CC and GBM samples. Small RNA sequencing reveal significant (two-tailed t-test) genome-wide C-to-U hypo-editing in five glioblastoma multiforme compared to six FC (p=0.004) and six CC samples (p=0.004).

Supplemental Figure S12

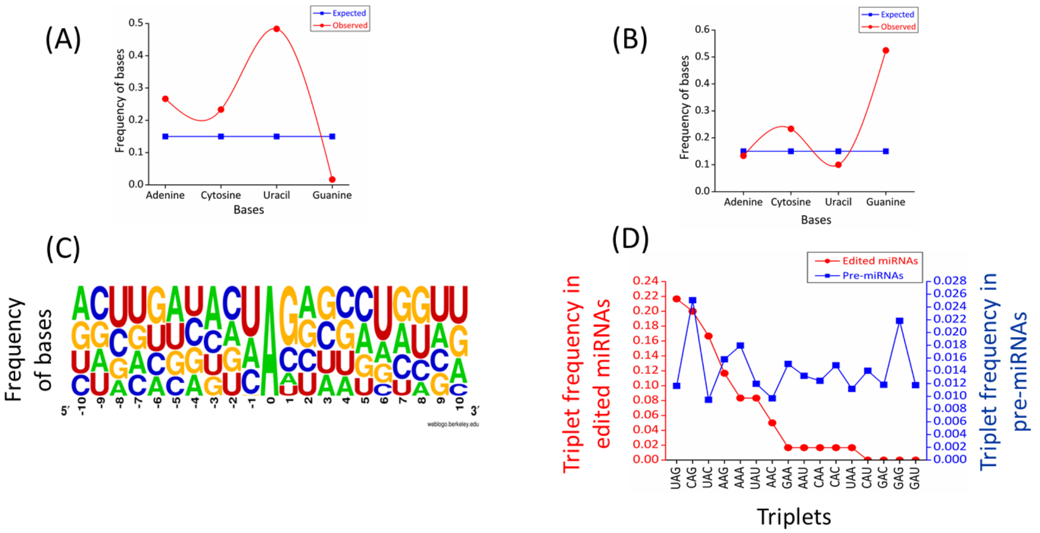

S12: A-to-I editing sites have a distinct sequence motif. (A) And (B) The Y-axis shows the frequency of bases one base upstream (A) and downstream (B), respectively, of the edited adenosine. There is a preference for “U” (and aversion for “G”) and preference for “G” in the upstream and downstream, respectively, of the edited adenosine. (C) Motif analysis 10 bases upstream and downstream of the edited adenosine revealed “UAG” as the preferred motif for ADAR family members. (D) The primary and the secondary y-axis represent triplet frequencies in edited and pre-miRNAs, respectively. X-axis shows the 16 possible triplets with “A” in the middle. The figure shows that UAG is not enriched in pre-miRNAs and that “UAG” is indeed a preferred motif for ADAR family members.

Supplemental Table S1: Samples/Data used in the study

Tissues	Sample state	Number_of_samples_analyzed	Data source	Accession Id
Prefrontal cortex	Healthy	6	Publicly available	SRP052236
Frontal cortex (FC)	Healthy	6	In-house	SRP063390
Corpus callosum (CC)	Healthy	6	In-house	SRP063390
Brain	Healthy	3	2 In-house and 1 publicly available	SRP063390 and SRR950878
Lung	Healthy	6	Publicly available	SRA052320
Islet of Langerhans	Healthy	2	Publicly available	SRR871609, SRR871652
Skin	Healthy	5	Publicly available	SRA044989
Kidney	Healthy	5	Publicly available	SRA025095
Ovary	Healthy	1	In-house	SRP063390
Testes	Healthy	1	In-house	SRP063390
Heart	Healthy	5	Publicly available	SRP021193
Peripheral blood mononuclear cells (PBMC)	Healthy	1	Publicly available	SRR353657
Liver	Healthy	3	Publicly available	SRP002272
Glioblastoma multiforme (GBM)	Diseased	5	In-house	SRP063390

Details of in-house samples

Samples	Age (in years)	Gender	Time elapsed after death	Tissue collected
Individual 1	55	Male	7 hours	FC, CC
Individual 2	35	Male	6 hours 20 minutes	FC, CC
Individual 3	27	Male	6 hours 30 minutes	FC, CC
Individual 4	45	Female	4 hours	FC, CC
Individual 5	40	Male	7 hours	FC, CC
Individual 6	23	Male	6 hours 20 minutes	FC, CC

Commercial RNA
Clonetech Brain total RNA (Cat No.- 636530)
Stratagene Brain total RNA (Cat No.- 540005)
Stratagene Testes total RNA (Cat No.- 540049)
Stratagene Ovary total RNA (Cat No.- 540071)

GBM Samples	Age (in Years)	Gender
GBM 1	48	Male
GBM 2	40	Male
GBM 3	40	Male
GBM 4	50	Male
GBM 5	67	Male

Supplemental Table S2: Non-redundant canonical miRNA editing events

A-to-I editing events (Total = 60)

#CHROM	START	END	STRAND	miRNA	Editing position in pre-miRNA	Seed
chr9	96941180	96941181	+	hsa-let-7d-3p	66	Yes
chr19	52196094	52196095	+	hsa-let-7e-3p	57	Yes
chr11	122023003	122023004	-	hsa-miR-100-5p	13	No
chr12	97885695	97885696	+	hsa-miR-1251-5p	10	Yes
chr2	25551538	25551539	-	hsa-miR-1301-3p	52	Yes
chr11	57408725	57408726	+	hsa-miR-130a-3p	56	Yes
chr16	69967020	69967021	+	hsa-miR-140-5p	38	No
chr12	54731035	54731036	+	hsa-miR-148b-5p	37	No

chr8	141742703	141742704	-	hsa-miR-151a-3p	49	Yes
chr17	6920962	6920963	-	hsa-miR-195-3p	58	Yes
chr1	1102543	1102544	+	hsa-miR-200b-3p	61	Yes
chr14	104583776	104583777	-	hsa-miR-203b-3p	64	No
chr17	1953601	1953602	-	hsa-miR-212-3p	73	Yes
chr1	172107971	172107972	-	hsa-miR-214-3p	76	Yes
chr19	13947155	13947156	-	hsa-miR-24-2-5p	18	Yes
chr19	13947267	13947268	-	hsa-miR-27a-3p	64	No
chr19	13947275	13947276	-	hsa-miR-27a-3p	56	Yes
chr19	13947321	13947322	-	hsa-miR-27a-3p	10	No
chr9	97847791	97847792	+	hsa-miR-27b-3p	66	Yes
chr9	97847789	97847790	+	hsa-miR-27b-3p	64	Yes
chr22	22007331	22007332	+	hsa-miR-301b-3p	63	No
chr10	97824086	97824087	-	hsa-miR-3157-3p	70	No
chr16	593349	593350	+	hsa-miR-3176	74	No
chr14	101340894	101340895	+	hsa-miR-337-3p	66	Yes
chr5	179442327	179442328	-	hsa-miR-340-3p	70	No
chr8	27559221	27559222	+	hsa-miR-3622a-5p	29	No
chr2	12339264	12339265	+	hsa-miR-3681-5p	10	Yes
chr21	9826243	9826244	+	hsa-miR-3687	42	Yes
chr14	101531981	101531982	+	hsa-miR-369-3p	48	Yes
chrX	73438406	73438407	-	hsa-miR-374b-3p	47	Yes
chr14	101507126	101507127	+	hsa-miR-376a-1-5p	9	Yes
chr14	101506419	101506420	+	hsa-miR-376a-2-5p	15	Yes

chr14	101506073	101506074	+	hsa-miR-376c-3p	48	Yes
chr14	101528439	101528440	+	hsa-miR-377-3p	54	No
chr14	101488411	101488412	+	hsa-miR-379-5p	10	Yes
chr14	101512307	101512308	+	hsa-miR-381-3p	52	Yes
chr14	101489680	101489681	+	hsa-miR-411-5p	20	Yes
chrX	73438242	73438243	-	hsa-miR-421	54	Yes
chr9	116971744	116971745	+	hsa-miR-455-5p	32	No
chr8	125834233	125834234	+	hsa-miR-4662a-5p	8	Yes
chr17	15154965	15154966	-	hsa-miR-4731-3p	48	Yes
chr1	176998525	176998526	-	hsa-miR-488-3p	56	Yes
chr17	6921316	6921317	-	hsa-miR-497-5p	25	Yes
chrX	133680421	133680422	-	hsa-miR-503-5p	7	Yes
chrX	146271285	146271286	-	hsa-miR-513c-5p	20	Yes
chr14	101513674	101513675	+	hsa-miR-539-5p	18	No
chr7	5535482	5535483	-	hsa-miR-589-3p	66	Yes
chr7	5535483	5535484	-	hsa-miR-589-3p	65	Yes
chr8	10892750	10892751	-	hsa-miR-598-3p	62	Yes
chr10	53059385	53059386	+	hsa-miR-605-3p	54	Yes
chr10	53059349	53059350	+	hsa-miR-605-5p	18	Yes
chr14	31483925	31483926	-	hsa-miR-624-5p	23	Yes
chr19	40788530	40788531	-	hsa-miR-641-5p	18	Yes
chr19	40788531	40788532	-	hsa-miR-641-5p	17	Yes
chr11	59976570	59976571	-	hsa-miR-6503-3p	59	Yes
chr1	220373943	220373944	-	hsa-miR-664a-5p	18	Yes

chr14	101514298	101514299	+	hsa-miR-889-3p	62	No
chr3	189547768	189547769	+	hsa-miR-944	59	Yes
chr21	17911420	17911421	+	hsa-miR-99a-5p	13	No
chr19	52195910	52195911	+	hsa-miR-99b-3p	47	Yes

C-to-U editing events (Total=56)

#CHROM	START	END	STRAND	miRNA	Editing position in pre-miRNA	Seed
chr11	122022991	122022992	-	hsa-mir-100-5p	25	No
chr11	122022999	122023000	-	hsa-mir-100-5p	17	Yes
chr7	99691669	99691670	-	hsa-mir-106b-5p	28	No
chr18	56118329	56118330	+	hsa-mir-122-5p	25	No
chr19	52196530	52196531	+	hsa-mir-125a-5p	25	No
chr1	171070890	171070891	-	hsa-mir-1295a	57	No
chr10	65132734	65132735	-	hsa-mir-1296-3p	74	No
chr10	105154102	105154103	-	hsa-mir-1307-5p	56	No
chr3	44155727	44155728	+	hsa-mir-138-1-5p	25	Yes
chr5	148808552	148808553	+	hsa-mir-143-3p	73	No
chr17	46114558	46114559	-	hsa-mir-152-3p	55	Yes
chr19	13985586	13985587	+	hsa-mir-181c-3p	75	No
chr19	13985547	13985548	+	hsa-mir-181c-5p	36	No
chr3	49058117	49058118	-	hsa-mir-191-5p	25	No
chr13	92003206	92003207	+	hsa-mir-19a-3p	63	No

chr12	7072912	7072913	+	hsa-mir-200c-3p	52	No
chr9	73424957	73424958	-	hsa-mir-204-5p	43	No
chr14	23857821	23857822	-	hsa-mir-208a-3p	54	No
chr14	23887216	23887217	-	hsa-mir-208b-3p	56	No
chr17	57918645	57918646	+	hsa-mir-21-5p	20	No
chr9	131154960	131154961	-	hsa-mir-219a-2-5p	33	No
chr17	1617221	1617222	-	hsa-mir-22-3p	60	Yes
chr19	13947426	13947427	-	hsa-mir-23a-3p	47	Yes
chr9	97847548	97847549	+	hsa-mir-23b-3p	60	Yes
chr9	97847555	97847556	+	hsa-mir-23b-3p	67	No
chr7	130561521	130561522	-	hsa-mir-29a-3p	48	Yes
chr6	72113309	72113310	-	hsa-mir-30a-5p	15	No
chr6	72113308	72113309	-	hsa-mir-30a-5p	16	No
chr8	135817172	135817173	-	hsa-mir-30d-5p	16	No
chr1	41220052	41220053	+	hsa-mir-30e-5p	27	No
chr1	41220051	41220052	+	hsa-mir-30e-5p	26	No
chr14	101522614	101522615	+	hsa-mir-323b-3p	60	No
chr19	46142271	46142272	-	hsa-mir-330-3p	74	No
chr22	42296966	42296967	+	hsa-mir-33a-5p	20	No
chr14	100576067	100576068	+	hsa-mir-342-3p	77	No
chr1	9211800	9211801	-	hsa-mir-34a-5p	36	No
chrX	73438431	73438432	-	hsa-mir-374b-5p	22	No
chr2	219866380	219866381	-	hsa-mir-375	50	No
chr14	101531658	101531659	+	hsa-mir-409-5p	23	No

chrX	73438246	73438247	-	hsa-mir-421	50	Yes
chr17	28444165	28444166	+	hsa-mir-423-3p	70	No
chr3	49057642	49057643	-	hsa-mir-425-5p	25	No
chr3	49057646	49057647	-	hsa-mir-425-5p	21	Yes
chr17	27188432	27188433	-	hsa-mir-451a	26	No
chr17	27188431	27188432	-	hsa-mir-451a	27	No
chr9	20716128	20716129	+	hsa-mir-491-5p	26	No
chr17	40666235	40666236	+	hsa-mir-5010-5p	31	No
chr8	10892737	10892738	-	hsa-mir-598-3p	75	No
chr8	10892779	10892780	-	hsa-mir-598-5p	33	No
chr19	46522226	46522227	+	hsa-mir-769-5p	38	No
chr21	17911432	17911433	+	hsa-mir-99a-5p	25	No
chr21	17911424	17911425	+	hsa-mir-99a-5p	17	Yes
chr21	17911436	17911437	+	hsa-mir-99a-5p	29	No
chr21	17911431	17911432	+	hsa-mir-99a-5p	24	No
chr19	52195882	52195883	+	hsa-mir-99b-5p	19	No
chr2	219267390	219267391	+	hsa-mir-26b-5p	23	No

Tissue wise distribution of A-to-I and C-to-U events

1. **Prefrontal cortex**

			Alternate/Total reads						
miRNA	Editing position in pre-miRNA	Mismatch	SRR1759212	SRR1759213	SRR1759214	SRR1759215	SRR1759216	SRR1759217	Presence in seed
hsa-mir-411-5p	20	AG	3747/16488	5544/24437	4475/20905	2966/12445	5461/23792	1805/5344	Yes
hsa-mir-381-3p	52	AG	574/7974	679/9973	512/9594	332/5177	573/8153	248/2185	Yes
hsa-mir-99a-5p	13	AG	1265/33302	1684/43984	1072/28015	917/26399	1172/28912	1278/34929	No
hsa-mir-1301-3p	52	AG	166/1217	279/1773	157/1352	180/1044	228/1415	131/828	Yes
hsa-mir-27b-3p	64	AG	300/61976	385/105482	293/90232	225/63542	409/89414	298/48744	Yes
hsa-mir-151a-3p	49	AG	61/1682	76/2637	48/2354	48/1578	78/2238	40/1080	Yes
hsa-mir-1251-5p	10	AG	30/201	19/126	15/139	15/124	12/112	17/192	Yes
hsa-mir-376c-3p	48	AG	22/341	29/446	16/294	14/241	17/211	76/1049	Yes
hsa-mir-130a-3p	56	AG	38/3038	29/3484	23/2923	27/1975	32/2937	32/2206	Yes
hsa-mir-589-3p	66	AG	10/29	11/37	11/29	7/15	12/20	-	Yes
hsa-mir-376a-1-5p	9	AG	22/1060	23/1219	12/974	17/742	15/905	26/572	Yes
hsa-mir-889-3p	62	AG	26/2576	17/2175	13/1649	13/1247	16/2667	9/790	No
hsa-mir-200b-3p	61	AG	7/59	10/111	-	-	15/105	4/38	Yes
hsa-mir-99b-3p	47	AG	10/348	9/423	-	13/281	10/307	14/397	Yes
hsa-mir-598-3p	62	AG	26/5131	21/3710	-	-	16/2720	26/3284	Yes

hsa-mir-421	54	AG	15/1492	-	16/1785	15/1123	18/1853	10/608	Yes
hsa-mir-340-3p	70	AG	11/732	13/952	10/1018	-	-	-	No
hsa-mir-148b-5p	37	AG	9/397	-	11/525	7/417	10/504	7/357	No
hsa-mir-301b-3p	63	AG	8/311	12/455	9/424	-	7/365	-	No
hsa-mir-27a-3p	56	AG	12/1322	14/1974	-	-	11/1421	13/1302	Yes
hsa-mir-664a-5p	18	AG	5/98	12/123	7/71	-	10/131	8/65	Yes
hsa-mir-539-5p	18	AG	-	12/289	6/208	6/171	9/263	10/211	No
hsa-let-7e-3p	57	AG	-	8/296	-	-	7/237	-	Yes
hsa-mir-337-3p	66	AG	-	5/64	6/38	5/44	-	-	Yes
hsa-mir-455-5p	32	AG	-	6/233	-	-	-	-	No
hsa-mir-641-5p	17	AG	-	5/126	-	-	8/91	-	Yes
hsa-let-7d-3p	66	AG	-	-	19/1694	17/1542	11/1449	-	Yes
hsa-mir-641-5p	18	AG	-	-	5/131	-	-	-	Yes
hsa-mir-3681-5p	10	AG	-	-	-	-	7/19	-	Yes
hsa-mir-377-3p	54	AG	-	-	-	-	-	5/41	No

			Alternate/Total reads						
miRNA	Editing position in pre-miRNA	Mismatch	SRR1759212	SRR1759213	SRR1759214	SRR1759215	SRR1759216	SRR1759217	Presence in seed
hsa-mir-99a-5p	25	CU	441/33767	719/44541	439/28506	435/26881	431/29429	633/35547	No
hsa-mir-100-5p	25	CU	318/128781	594/215457	510/166904	388/142218	439/153969	413/134217	No
hsa-mir-29a-3p	48	CU	-	166/94709	124/68188	117/54417	128/69832	-	Yes
hsa-mir-33a-5p	20	CU	-	9/707	-	-	-	-	No

hsa-mir-26b-5p	23	CU	-	56/26954	-	-	-	-	No
hsa-mir-425-5p	25	CU	-	-	-	3/13	3/15	-	No

2. Brain total RNA

			Alternate/Total reads			
miRNA	Editing position in pre-miRNA	Mismatch	Clonetech brain RNA	Stratagene brain RNA	SRR950878	Presence in seed
hsa-let-7d-3p	66	AG	24/6026	20/4483	11/1126	Yes
hsa-let-7e-3p	57	AG	-	7/440	-	Yes
hsa-miR-1251-5p	10	AG	-	10/186	-	Yes
hsa-miR-1301-3p	52	AG	14/489	-	46/291	Yes
hsa-miR-148b-5p	37	AG	-	11/842	-	No
hsa-miR-151a-3p	49	AG	6/193	115/5878	49/2104	Yes
hsa-miR-200b-3p	61	AG	-	19/2224	-	Yes
hsa-miR-27a-3p	56	AG	-	-	10/825	Yes
hsa-miR-27b-3p	64	AG	77/11341	235/134843	121/72252	Yes
hsa-miR-340-3p	70	AG	-	10/982	-	No
hsa-miR-369-3p	48	AG	-	20/4707	-	Yes
hsa-miR-376a-1-5p	9	AG	-	17/3561	7/49	Yes
hsa-miR-377-3p	54	AG	-	4/62	-	No
hsa-miR-381-3p	52	AG	12/275	61/3150	176/2939	Yes
hsa-miR-411-5p	20	AG	32/174	7266/9284	2868/13729	Yes
hsa-miR-421	54	AG	-	-	14/1021	Yes

hsa-miR-539-5p	18	AG	5/51	17/408	7/171	No
hsa-miR-589-3p	66	AG	4/13	28/103	7/10	Yes
hsa-miR-598-3p	62	AG	-	64/15248	-	Yes
hsa-miR-641-5p	18	AG	-	14/271	-	Yes
hsa-miR-664a-5p	18	AG	-	9/185	-	Yes
hsa-miR-889-3p	62	AG	-	67/12369	13/1847	No
hsa-miR-99a-5p	13	AG	15/391	238/3751	372/9344	No
hsa-miR-99b-3p	47	AG	16/588	-	8/171	Yes

			Alternate/Total reads			
miRNA	Editing position in pre-miRNA	Mismatch	Clonetech brain RNA	Stratagene brain RNA	SRR950878	Presence in seed
hsa-miR-100-5p	25	CU	110/1968	2227/60919	1045/114686	No
hsa-miR-99a-5p	25	CU	14/423	43/4212	300/9825	No
hsa-miR-100-5p	17	CU	-	253/58785	-	Yes
hsa-miR-30a-5p	15	CU	-	129/23913	-	No
hsa-miR-181c-5p	36	CU	-	115/23469	-	No
hsa-miR-342-3p	77	CU	-	62/18105	-	No
hsa-miR-99b-5p	19	CU	-	71/26500	-	No
hsa-miR-99a-5p	17	CU	-	19/3819	-	Yes
hsa-miR-330-3p	74	CU	-	9/767	-	No
hsa-miR-125a-5p	25	CU	-	84/45083	-	No
hsa-miR-23b-3p	60	CU	-	7/448	-	Yes
hsa-miR-29a-3p	48	CU	-	-	34/10380	Yes

3. Lung

			Alternate/Total reads						
miRNA	Editing position in pre-miRNA	Mismatch	SRR493961	SRR493963	SRR493965	SRR493967	SRR493969	SRR493971	Presence in Seed
hsa-let-7d-3p	66	AG	-	-	13/1701	-	-	14/1261	Yes
hsa-miR-100-5p	13	AG	-	126/68530	235/62799	209/113521	258/86109	183/74051	No
hsa-miR-140-5p	38	AG	-	74/21140	-	-	-	-	No
hsa-miR-148b-5p	37	AG	-	20/2733	-	13/1499	13/1443	-	No
hsa-miR-151a-3p	49	AG	-	30/4879	40/6955	59/8642	46/8192	30/6753	Yes
hsa-miR-200b-3p	61	AG	87/12905	110/14617	79/11047	118/13033	105/15180	119/13163	Yes
hsa-miR-24-2-5p	18	AG	-	14/1097	13/542	-	8/481	-	Yes
hsa-miR-27a-3p	56	AG	125/45339	213/86678	223/62142	287/107320	205/68832	205/63884	Yes
hsa-miR-27a-5p	10	AG	-	23/2545	47/3546	26/2582	67/3444	48/6172	No
hsa-miR-376a-1-5p	9	AG	7/124	7/144	-	-	-	-	Yes
hsa-miR-376c-3p	48	AG	-	10/602	-	-	-	-	Yes
hsa-miR-379-5p	10	AG	15/1035	38/1601	68/1716	44/3872	73/2298	56/1834	Yes
hsa-miR-381-3p	52	AG	119/890	274/2426	193/1038	773/2974	110/467	236/1160	Yes
hsa-miR-411-5p	20	AG	377/946	455/963	449/640	863/1428	545/753	529/759	Yes
hsa-miR-455-5p	32	AG	19/921	120/2248	56/1100	53/1723	65/1178	49/1041	No
hsa-miR-4662a-5p	8	AG	-	-	-	-	-	4/35	Yes

hsa-miR-497-5p	25	AG	18/2484	76/5950	55/2391	26/3222	61/2567	43/2026	Yes
hsa-miR-503-5p	7	AG	-	6/271	-	-	-	-	Yes
hsa-miR-589-3p	66	AG	-	3/13	-	3/19	-	-	Yes
hsa-miR-6503-3p	59	AG	-	3/11	-	9/24	5/17	8/17	Yes
hsa-miR-664a-5p	18	AG	-	6/289	9/239	-	-	-	Yes
hsa-miR-944	59	AG	-	-	-	-	-	6/77	Yes
hsa-miR-99a-5p	13	AG	433/11899	2913/27073	1595/10175	1372/17142	1509/11495	1026/10386	No

			Alternate/Total reads						
miRNA	Editing position in pre-miRNA	Mismatch	SRR493961	SRR493963	SRR493965	SRR493967	SRR493969	SRR493971	Presence in Seed
hsa-miR-451a	26	CU	2168/1487393	-	-	267/166897	868/675672	-/-	No
hsa-miR-19a-3p	63	CU	21/3608	-	13/1086	12/1102	14/842	20/850	No
hsa-miR-425-5p	25	CU	5/80	-	-	-	-	-/-	No
hsa-miR-30a-5p	16	CU	296/219833	974/635825	1265/889564	-	1963/1137995	971/665914	No
hsa-miR-29a-3p	48	CU	-	148/88125	-	-	108/64202	-/-	Yes
hsa-miR-191-5p	25	CU	-	45/18430	-	-	-	-/-	No
hsa-miR-30e-5p	26	CU	-	-	294/198560	-	359/233400	273/181024	No
hsa-miR-23a-3p	47	CU	-	-	5/116	-	-	-/-	Yes
hsa-miR-1295a	57	CU	-	-	3/15	-	-	-/-	No
hsa-miR-200c-3p	52	CU	-	-	-	150/62867	155/76255	-/-	No

hsa-miR-106b-5p	28	CU	-	-	-	-	38/12601	38/14595	No

4. Heart

			Alternate/Total reads					
miRNA	Editing position in pre-miRNA	Mismatch	SRR831005	SRR831007	SRR831008	SRR831009	SRR831010	Presence in Seed
hsa-miR-376c-3p	48	AG	-	4/69	-	-	-	Yes
hsa-miR-381-3p	52	AG	34/1397	57/2046	50/2157	55/1942	42/1847	Yes
hsa-miR-411-5p	20	AG	39/1328	137/2820	111/3160	142/3191	119/2690	Yes
hsa-miR-589-3p	66	AG	-	5/16	-	-	-	Yes
hsa-miR-605-3p	54	AG	9/19	10/31	11/34	8/35	8/29	Yes
hsa-miR-6503-3p	59	AG	-	5/10	-	11/25	7/20	Yes
hsa-miR-99a-5p	13	AG	87/34247	184/56903	136/39498	130/40037	132/39118	No

			Alternate/Total reads					
miRNA	Editing position in pre-miRNA	Mismatch	SRR831005	SRR831007	SRR831008	SRR831009	SRR831010	Presence in Seed
hsa-miR-99a-5p	25	CU	659/34633	888/59960	580/41610	694/42259	740/41262	No

hsa-miR-23b-3p	67	CU	54/17799	122/27903	83/22743	-	86/29212	No
hsa-miR-100-5p	25	CU	116/59602	226/96834	-	441/203378	223/94572	No
hsa-miR-208b-3p	56	CU	-	373/218889	392/214754	396/221581	495/272680	No
hsa-miR-30a-5p	15	CU	-	294/184197	206/136705	-	-	No
hsa-miR-208a-3p	54	CU	-	12/1633	-	-	-	No

5. Kidney

			Alternate/Total reads					
miRNA	Editing position in pre-miRNA	Mismatch	SRR070240	SRR070242	SRR070244	SRR070246	SRR070248	Presence in Seed
hsa-miR-151a-3p	49	AG	39/5985	29/5099	-	27/5084	-	Yes
hsa-miR-200b-3p	61	AG	333/14414	349/19524	140/12545	200/19129	51/6587	Yes
hsa-miR-27a-3p	56	AG	-	-	18/3992	-	-	Yes
hsa-miR-27a-5p	10	AG	-	-	-	-	7/454	No
hsa-miR-376a-1-5p	9	AG	4/67	-	-	-	-	Yes
hsa-miR-379-5p	10	AG	20/1500	17/935	-	-	-	Yes
hsa-miR-381-3p	52	AG	179/578	110/501	142/498	151/684	38/232	Yes
hsa-miR-411-5p	20	AG	127/233	196/600	202/568	351/1108	48/131	Yes
hsa-miR-421	54	AG	8/61	8/76	-	-	-	Yes
hsa-miR-605-3p	54	AG	-	6/15	-	-	5/31	Yes
hsa-miR-664a-5p	18	AG	18/821	-	-	-	-	Yes

hsa-miR-99a-5p	13	AG	99/9838	143/14577	78/8301	139/15913	62/8363	No

			Alternate/Total reads					
miRNA	Editing position in pre-miRNA	Mismatch	SRR070240	SRR070242	SRR070244	SRR070246	SRR070248	Presence in Seed
hsa-miR-30a-5p	16	CU	1651/1242033	1342/1028060	700/510147	914/580062	571/311770	No
hsa-miR-30e-5p	27	CU	121/53109	-	-	-	-	No
hsa-miR-100-5p	25	CU	102/57370	-	-	-	147/92378	No
hsa-miR-99a-5p	25	CU	-	38/14614	-	-	-	No
hsa-miR-22-3p	60	CU	-	23/6379	-	23/5749	-	Yes
hsa-miR-30a-5p	15	CU	-	-	742/510187	-	406/311791	No
hsa-miR-29a-3p	48	CU	-	-	47/19894	74/30978	-	Yes
hsa-miR-152-3p	55	CU	-	-	4/55	-	-	Yes
hsa-miR-19a-3p	63	CU	-	-	-	-	9/191	No

6. Skin

			Alternate/Total reads					
miRNA	Editing position in pre-miRNA	Mismatch	SRR330915	SRR330919	SRR330921	SRR330922	SRR330923	Presence in Seed
hsa-miR-200b-3p	61	AG	203/22691	149/20546	241/27465	215/26167	232/27117	Yes
hsa-miR-27a-3p	56	AG	562/131991	325/131615	373/160373	312/134740	409/144912	Yes
hsa-miR-27a-5p	10	AG	31/1947	-	14/1678	-	24/1747	No
hsa-miR-376a-1-5p	9	AG	6/139	-	-	-	-	Yes
hsa-miR-376c-3p	48	AG	-	11/836	16/749	18/1140	-	Yes
hsa-miR-379-5p	10	AG	-	58/6559	56/6941	89/11064	82/7823	Yes
hsa-miR-381-3p	52	AG	723/2728	389/2425	855/4448	1185/4979	890/3136	Yes
hsa-miR-411-5p	20	AG	720/2037	962/3436	1076/3573	1005/2856	793/2379	Yes
hsa-miR-6503-3p	59	AG	9/16	3/17	9/25	13/33	4/25	Yes
hsa-miR-664a-5p	18	AG	-	-	9/500	7/431	-	Yes
hsa-miR-944	59	AG	12/1240	-	12/1870	16/1570	13/1523	Yes
hsa-miR-99a-5p	13	AG	628/47478	273/34621	743/56807	759/64519	722/51078	No

			Alternate/Total reads					
miRNA	Editing position in pre-miRNA	Mismatch	SRR330915	SRR330919	SRR330921	SRR330922	SRR330923	Presence in Seed
hsa-miR-19a-3p	63	CU	10/266	15/375	-	6/181	-	No
hsa-miR-23b-3p	60	CU	9/426	7/247	6/238	-	-	Yes

hsa-miR-23a-3p	47	CU	-	-	-	11/935	-	Yes
hsa-miR-425-5p	21	CU	-	-	-	-	4/17	Yes
hsa-miR-22-3p	60	CU	-	-	-	-	31/10850	Yes

7. Liver

			Alternate/Total reads			
miRNA	Editing position in pre-miRNA	Mismatch	SRR039611	SRR039612	SRR039613	Presence in Seed
hsa-miR-6503-3p	59	AG	26/34	100/123	33/49	Yes
hsa-miR-411-5p	20	AG	21/493	33/2521	31/1718	Yes
hsa-miR-214-3p	76	AG	21/1432	-	-	Yes
hsa-miR-664a-5p	18	AG	9/334	-	-	Yes
hsa-miR-200b-3p	61	AG	7/442	-	-	Yes

			Alternate/Total reads			
miRNA	Editing position in pre-miRNA	Mismatch	SRR039611	SRR039612	SRR039613	Presence in Seed
hsa-miR-122-5p	25	CU	3061/1630995	-	-	No

hsa-miR-30d-5p	16	CU	385/59604	204/62803	132/44398	No
hsa-miR-99a-5p	29	CU	517/151934	-	-	No
hsa-miR-30a-5p	15	CU	218/38955	144/63296	60/29445	No
hsa-miR-30e-5p	26	CU	67/5483	52/9465	-	No
hsa-miR-30e-5p	27	CU	42/5491	62/9464	41/6011	No
hsa-miR-143-3p	73	CU	60/13247	44/17800	-	No
hsa-miR-21-5p	20	CU	92/31075	-	-	No
hsa-miR-30a-5p	16	CU	99/39030	-	-	No
hsa-miR-191-5p	25	CU	44/10962	-	-	No
hsa-miR-99a-5p	25	CU	246/152163	-	-	No
hsa-miR-423-3p	70	CU	9/437	-	-	No
hsa-miR-23b-3p	60	CU	4/29	11/95	-	Yes
hsa-miR-99a-5p	24	CU	221/152881	-	-	No

8. Islets of Langerhans

			Alternate/Total reads		
miRNA	Editing position in pre-miRNA	Mismatch	SRR871609	SRR871652	Presence in Seed
hsa-miR-200b-3p	61	AG	1558/24252	20/255	Yes
hsa-miR-381-3p	52	AG	15440/32955	42/88	Yes
hsa-miR-151a-3p	49	AG	496/19569	-	Yes
hsa-miR-411-5p	20	AG	14610/25540	62/151	Yes

hsa-miR-379-5p	10	AG	960/81163	10/998	Yes
hsa-miR-27a-3p	56	AG	290/25298	-	Yes
hsa-miR-421	54	AG	60/90	4/11	Yes
hsa-miR-376c-3p	48	AG	50/558	-	Yes
hsa-miR-99a-5p	13	AG	53/5313	-	No
hsa-miR-148b-5p	37	AG	37/4111	-	No
hsa-miR-27a-3p	64	AG	57/25286	-	No
hsa-miR-589-3p	66	AG	3/10	-	Yes
hsa-miR-664a-5p	18	AG	-	5/47	Yes

			Alternate/Total reads		
miRNA	Editing position in pre-miRNA	Mismatch	SRR871609	SRR871652	Presence in Seed
hsa-miR-200c-3p	52	CU	459/249969	-	No
hsa-miR-30a-5p	16	CU	584/430597	-	No
hsa-miR-409-5p	23	CU	11/475	-	No
hsa-miR-106b-5p	28	CU	16/2175	-	No
hsa-miR-100-5p	25	CU	-	6/207	No

9. Testes

			Alternate/Total reads	
miRNA	Editing position in pre-miRNA	Mismatch	Stratagene Testes RNA	Presence in Seed

hsa-miR-411-5p	20	AG	518/9697	Yes
hsa-miR-381-3p	52	AG	79/1424	Yes
hsa-miR-200b-3p	61	AG	60/4171	Yes
hsa-miR-99a-5p	13	AG	75/12839	No
hsa-miR-6503-3p	59	AG	8/10	Yes
hsa-miR-151a-3p	49	AG	14/2479	Yes

			Alternate/Total reads	
miRNA	Editing position in pre-miRNA	Mismatch	Stratagene Testes RNA	Presence in Seed
hsa-miR-99a-5p	25	CU	338/12978	No
hsa-miR-100-5p	25	CU	263/87795	No

10. Ovary

			Alternate/Total reads	
miRNA	Editing position in pre-miRNA	Mismatch	Stratagene Ovary RNA	Presence in Seed
hsa-miR-411-5p	20	AG	1372/29528	Yes
hsa-miR-381-3p	52	AG	297/10871	Yes
hsa-miR-605-3p	54	AG	8/11	Yes
hsa-miR-99a-5p	13	AG	236/123147	No

hsa-miR-503-5p	7	AG	15/474	Yes
hsa-miR-664a-5p	18	AG	6/186	Yes

			Alternate/Total reads	
miRNA	Editing position in pre-miRNA	Mismatch	Stratagene Ovary RNA	Presence in Seed
hsa-miR-99a-5p	25	CU	4113/124265	No
hsa-miR-100-5p	25	CU	2516/615128	No
hsa-miR-29a-3p	48	CU	158/45443	Yes
hsa-miR-33a-5p	20	CU	12/392	No
hsa-miR-451a	27	CU	101/55993	No
hsa-miR-342-3p	77	CU	18/4210	No

11. Peripheral Blood Mononuclear Cell (PBMC)

			Alternate/Total reads	
miRNA	Editing position in pre-miRNA	Mismatch	SRR353657	Presence in Seed
hsa-miR-605-3p	54	AG	75/483	Yes
hsa-miR-381-3p	52	AG	36/469	Yes
hsa-miR-6503-3p	59	AG	23/96	Yes
hsa-miR-411-5p	20	AG	17/1451	Yes
hsa-miR-421	54	AG	9/840	Yes

			Alternate/Total reads	
miRNA	Editing position in pre-miRNA	Mismatch	SRR353657	Presence in Seed
hsa-miR-29a-3p	48	CU	218/108375	Yes
hsa-miR-425-5p	21	CU	6/90	Yes
hsa-miR-19a-3p	63	CU	28/6870	No

12. Frontal cortex (FC)

			Alternate/Total reads						
miRNA	Editing position in pre-miRNA	Mismatch	FC1	FC2	FC3	FC4	FC5	FC6	Presence in Seed
hsa-let-7d-3p	66	AG	22/2325	22/2520	19/2308	53/9316	93/16377	16/3010	Yes
hsa-let-7e-3p	57	AG	-	7/264	12/252	21/1221	38/1550	-	Yes
hsa-miR-1251-5p	10	AG	29/167	18/150	-	47/299	22/184	-	Yes
hsa-miR-1301-3p	52	AG	82/1138	90/862	60/726	150/1959	235/3126	21/330	Yes
hsa-miR-130a-3p	56	AG	52/5612	53/5130	19/3146	80/14544	115/12582	-	Yes
hsa-miR-148b-5p	37	AG	-	15/641	10/384	14/931	22/2179	-	No
hsa-miR-151a-3p	49	AG	347/5326	251/5594	152/5209	365/12914	643/25618	104/7535	Yes

hsa-miR-195-3p	58	AG	-	-	-	7/335	-	-	Yes
hsa-miR-200b-3p	61	AG	7/104	-	5/80	12/206	28/470	10/222	Yes
hsa-miR-203b-3p	64	AG	-	-	-	23/722	13/336	-	No
hsa-miR-212-3p	73	AG	-	-	-	-	30/6245	-	Yes
hsa-miR-27a-3p	56	AG	44/6413	-	-	63/12077	209/18890	-	Yes
hsa-miR-27b-3p	64	AG	640/125789	467/116181	271/124561	689/248003	1611/695433	-	Yes
hsa-miR-27b-3p	66	AG	209/125682	-	-	-	-	-	Yes
hsa-miR-301b-3p	63	AG	16/1155	29/769	8/517	12/1552	47/4257	-	No
hsa-miR-3157-3p	70	AG	-	-	-	3/15	15/93	-	No
hsa-miR-3176	74	AG	-	-	-	-	5/84	-	No
hsa-miR-337-3p	66	AG	12/103	-	5/44	8/210	11/239	-	Yes
hsa-miR-340-3p	70	AG	15/1151	-	11/918	19/1122	54/4607	-	No
hsa-miR-3622a-5p	29	AG	-	-	-	-	6/126	-	No
hsa-miR-3681-5p	10	AG	15/68	11/89	7/29	19/114	13/157	-	Yes
hsa-miR-374b-3p	47	AG	-	-	-	10/839	-	-	Yes
hsa-miR-376a-1-5p	9	AG	53/488	49/469	32/269	42/627	146/1096	18/155	Yes
hsa-miR-376a-2-5p	15	AG	-	-	-	5/16	-	-	Yes
hsa-miR-376c-3p	48	AG	22/735	24/410	8/159	36/1274	41/1557	6/135	Yes
hsa-miR-377-3p	54	AG	9/85	11/81	-	5/89	31/292	-	No
hsa-miR-381-3p	52	AG	1426/16545	1552/22201	429/11966	3440/50981	3655/45413	399/5964	Yes
hsa-miR-411-5p	20	AG	15466/56575	19457/59822	6583/25591	28272/110696	52809/189959	2715/7961	Yes
hsa-miR-421	54	AG	60/2387	65/2432	28/2221	60/3875	161/14868	34/4657	Yes
hsa-miR-455-5p	32	AG	-	10/497	-	-	16/1339	-	No
hsa-miR-488-3p	56	AG	23/4916	-	-	24/6104	-	-	Yes

hsa-miR-497-5p	25	AG	-	-	-	-	27/7777	-	Yes
hsa-miR-539-5p	18	AG	160/7513	95/4580	67/2821	58/2574	221/9138	16/728	No
hsa-miR-589-3p	66	AG	13/36	15/27	8/15	10/28	31/88	-	Yes
hsa-miR-598-3p	62	AG	32/8449	58/11524	39/7137	162/37718	168/35763	19/2900	Yes
hsa-miR-605-5p	18	AG	-	-	-	3/16	-	-	Yes
hsa-miR-641-5p	18	AG	4/69	6/130	5/92	23/368	60/761	8/158	Yes
hsa-miR-641-5p	17	AG	-	-	-	11/368	-	-	Yes
hsa-miR-889-3p	62	AG	79/9686	95/15131	68/11265	91/20109	267/37721	-	No
hsa-miR-99a-5p	13	AG	2197/54508	2639/56674	1095/25304	3473/125115	3484/152194	899/45172	No
hsa-miR-99b-3p	47	AG	30/734	51/1040	32/640	64/2076	105/3359	10/666	Yes

			Alternate/Total reads						
miRNA	Editing position in pre-miRNA	Mismatch	FC1	FC2	FC3	FC4	FC5	FC6	Presence in Seed
hsa-miR-100-5p	25	CU	1560/127059	2057/191200	1521/93939	8077/688000	13794/766449	3514/624184	No
hsa-miR-99a-5p	25	CU	1396/56463	1371/59728	761/26855	3504/132237	5547/163318	1223/46977	No
hsa-miR-125a-5p	25	CU	212/105200	251/121339	342/136172	367/219997	709/379422	-	No
hsa-miR-99b-5p	19	CU	440/285723	-	-	-	2147/1236408	-	No

hsa-miR-374b-5p	22	CU	29/4098	17/3760	-	33/9652	76/25402	-	No
hsa-miR-409-5p	23	CU	8/440	-	-	-	21/828	-	No
hsa-miR-33a-5p	20	CU	-	9/759	7/300	-	25/2243	20/1088	No
hsa-miR-29a-3p	48	CU	-	94/53669	-	221/107639	396/230107	80/38306	Yes
hsa-miR-106b-5p	28	CU	-	-	9/718	-	-	-	No
hsa-miR-1296-3p	74	CU	-	-	-	8/78	7/117	-	No
hsa-miR-330-3p	74	CU	-	-	-	15/1392	27/3792	-	No
hsa-miR-769-5p	38	CU	-	-	-	271/177920	-	-	No
hsa-miR-181c-3p	75	CU	-	-	-	50/18463	-	-	No
hsa-miR-598-3p	75	CU	-	-	-	80/37689	-	-	No
hsa-miR-204-5p	43	CU	-	-	-	-	360/196734	-	No
hsa-miR-34a-5p	36	CU	-	-	-	-	106/43297	-	No
hsa-miR-491-5p	26	CU	-	-	-	-	18/1478	-	No
hsa-miR-219a-2-5p	33	CU	-	-	-	-	12/780	-	No
hsa-miR-375	50	CU	-	-	-	-	10/819	-	No

13. **Corpus Callosum (CC)**

			Alternate/Total reads						
miRNA	Editing position in pre-miRNA	Mismatch	CC1	CC2	CC3	CC4	CC5	CC6	Presence in Seed
hsa-let-7d-3p	66	AG	-	38/11774	-	34/6945	63/20738	27/5973	Yes

hsa-miR-1251-5p	10	AG	13/73	25/235	-	-	10/76	11/84	Yes
hsa-miR-1301-3p	52	AG	15/414	93/1507	15/324	33/903	72/2286	28/663	Yes
hsa-miR-130a-3p	56	AG	22/2003	58/5995	-	22/4781	39/6805	33/2618	Yes
hsa-miR-148b-5p	37	AG	-	15/1824	-	11/1142	17/3241	-	No
hsa-miR-151a-3p	49	AG	95/7157	339/23583	48/6195	238/16990	287/27145	158/13143	Yes
hsa-miR-200b-3p	61	AG	-	12/551	15/148	11/142	34/605	-	Yes
hsa-miR-27a-3p	56	AG	68/7367	-	-	55/7810	152/13524	-	Yes
hsa-miR-301b-3p	63	AG	-	21/2276	-	17/1678	-	-	No
hsa-miR-3157-3p	70	AG	-	9/63	5/21	-	9/195	-	No
hsa-miR-337-3p	66	AG	-	-	-	5/127	-	-	Yes
hsa-miR-3622a-5p	29	AG	-	-	-	5/104	-	-	No
hsa-miR-3681-5p	10	AG	-	7/31	-	41/112	-	-	Yes
hsa-miR-3687	42	AG	-	-	-	-	4/64	-	Yes
hsa-miR-376a-1-5p	9	AG	5/94	28/457	4/29	30/377	36/263	17/191	Yes
hsa-miR-376c-3p	48	AG	12/186	15/690	-	12/735	8/490	12/216	Yes
hsa-miR-377-3p	54	AG	-	9/94	-	7/73	8/87	-	No
hsa-miR-381-3p	52	AG	389/3881	1477/19133	100/2269	2196/48928	1199/17047	710/9762	Yes
hsa-miR-411-5p	20	AG	2806/8095	11715/37208	1065/3525	17374/64764	16588/58892	3633/9916	Yes
hsa-miR-421	54	AG	42/4340	102/14963	23/4276	77/10098	180/34945	37/7013	Yes
hsa-miR-4731-3p	48	AG	-	9/45	-	6/29	11/47	4/17	Yes
hsa-miR-513c-5p	20	AG	-	-	-	-	7/78	-	Yes
hsa-miR-539-5p	18	AG	7/454	60/4119	8/400	27/2073	102/5671	27/1589	No
hsa-miR-589-3p	66	AG	7/35	31/82	6/17	3/13	33/122	-	Yes

hsa-miR-589-3p	65	AG	-	-	-	-	6/122	-	Yes
hsa-miR-598-3p	62	AG	21/3692	43/10362	-	51/21976	43/14463	21/5618	Yes
hsa-miR-624-5p	23	AG	-	-	-	-	6/47	-	Yes
hsa-miR-641-5p	18	AG	9/122	41/597	7/123	34/410	60/1079	14/192	Yes
hsa-miR-641-5p	17	AG	-	11/596	-	-	-	-	Yes
hsa-miR-889-3p	62	AG	14/1779	58/9392	-	43/16870	48/11670	21/5066	No
hsa-miR-99a-5p	13	AG	570/48331	3278/166988	449/33216	1204/78510	1992/201499	1319/83355	No
hsa-miR-99b-3p	47	AG	10/704	53/2267	7/408	39/1929	39/2467	20/1395	Yes

			Alternate/Total reads						
miRNA	Editing position in pre-miRNA	Mismatch	CC1	CC2	CC3	CC4	CC5	CC6	Presence in Seed
hsa-miR-99a-5p	25	CU	2224/50196	4470/174154	1389/34018	3354/83329	11307/214715	2913/86727	No
hsa-miR-100-5p	25	CU	5220/346229	20706/2449487	6425/777728	16732/634301	40802/3215205	7179/1508761	No
hsa-miR-204-5p	43	CU	120/40768	228/105639	-	234/128599	301/157732	92/43706	No
hsa-miR-99b-5p	19	CU	441/283342	-	-	1070/608728	4243/2688957	-/-	No
hsa-miR-374b-5p	22	CU	36/10178	61/25436	-	36/12545	74/29404	40/13524	No
hsa-miR-33a-5p	20	CU	10/889	30/3379	-	-	42/1369	20/1911	No
hsa-miR-29a-3p	48	CU	-	162/101083	38/15474	131/57708	313/154702	71/36329	Yes
hsa-miR-23b-3p	60	CU	-	10/977	-	9/374	-	8/352	Yes

hsa-miR-219a-2-5p	33	CU	-	15/2756	-	-	19/1417	10/1143	No
hsa-miR-421	50	CU	-	4/53	-	-	-	-	Yes
hsa-miR-125a-5p	25	CU	-	-	-	354/116818	612/488619	-	No
hsa-miR-100-5p	17	CU	-	-	-	855/618595	-	-	Yes
hsa-miR-598-5p	33	CU	-	-	-	4/21	-	-	No
hsa-miR-106b-5p	28	CU	-	-	-	18/3256	31/7834	-	No
hsa-miR-181c-3p	75	CU	-	-	-	33/10436	-	-	No
hsa-miR-19a-3p	63	CU	-	-	-	18/3410	-	-	No
hsa-miR-1307-5p	56	CU	-	-	-	40/15608	-	-	No
hsa-miR-425-5p	25	CU	-	-	-	-	9/33	-	No
hsa-miR-323b-3p	60	CU	-	-	-	-	16/2040	-	No
hsa-miR-598-3p	75	CU	-	-	-	-	43/14518	-	No
hsa-miR-138-1-5p	25	CU	-	-	-	-	7/237	-	Yes
hsa-miR-5010-5p	31	CU	-	-	-	-	4/23	-	No

Supplemental Table S3: Novel A-to-I editing events identified in our study

miRNA	Position in precursor	Position in seed
hsa-mir-148b-5p	37	No
hsa-mir-203b-3p	64	No
hsa-mir-3622a-5p	29	No
hsa-mir-4731-3p	48	Seed
hsa-mir-605-3p	54	Seed
hsa-mir-944	59	Seed

Supplemental Table S4: Intra-individual variation (FC vs CC) for A-to-I editing

hsa-let-7e edited in four FC but none of the CC samples

			Alternate/Total reads					
miRNA	Location inside pre-miRNA	Seed	FC1	FC2	FC3	FC4	FC5	FC6
hsa-let-7e	57	Yes	-	7/264	12/252	21/1221	38/1550	-

Target prediction done by Target Scan Custom

Targets of unedited let-7e	Targets of edited let-7e
PHOX2B	FOXG1
EDEM3	GATA2
PURB	PICK1
BSN	
NFASC	

miRNAs edited in FC and not in CC samples (present in at least 2 FC samples)

			Alternate/Total reads					
miRNA	Location inside pre-miRNA	Seed	FC1	FC2	FC3	FC4	FC5	FC6
hsa-miR-203b	64	No	-	-	-	23/722	13/336	-
hsa-miR-27b	64	Yes	640/125789	467/116181	271/124561	689/248003	1611/695433	-
hsa-miR-340	70	No	15/1151		11/918	19/1122	54/4607	-
hsa-miR-455	32	No	-	10/497	-	-	-	-
hsa-miR-488	56	Yes	23/4916	-	-	24/6104	-	-

hsa-miR-4731 edited in four CC but none of the FC samples

			Alternate/Total reads					
miRNA	Location inside pre-miRNA	Seed	CC1	CC2	CC3	CC4	CC5	CC6
hsa-miR-4731	48	Yes	-	9/45	-	6/29	11/47	4/17

Supplemental Table S5A: Two- and three dimensional structural prediction analysis for pre-miRNAs in the unedited and edited (A-to-I) forms

pre-mirna	Edited position in pre-mirna	Unedited (MFE in kcal/mol)	Edited (MFE in kcal/mol)	2D-ΔΔG	Unedited (MFE in kcal/mol)	Edited (MFE in kcal/mol)	3D-ΔΔG	Edited miRNA motif	Motif in the fold-back structure
pre-mir-200b	61	-42.5	-49.2	-6.7				UAC	GCA
pre-mir-3176	74	-51.5	-58.2	-6.7				UAC	GCA
pre-mir-589	66	-42	-48.7	-6.7	-67.11	-64.21	2.9	AAC	GCU

pre-mir-148b	37	-30.7	-37.4	-6.7				UAC	GCA
pre-mir-889	62	-25.2	-31.9	-6.7				AAC	GCU
pre-let-7d	66	-42.7	-49.4	-6.7	-76.19	-78.42	-2.23	UAC	GCA
pre-mir-455	32	-41.6	-48.3	-6.7				UAC	GCA
pre-mir-3157	70	-51.5	-58.1	-6.6	-81.65	-81.76	-0.11	UAG	CCA
pre-mir-376a-1	9	-18.5	-25.1	-6.6	-48.36	-49.23	-0.87	UAG	CCA
pre-mir-376a-2	15	-29	-35.6	-6.6				UAG	CCA
pre-mir-377	54	-27.5	-34.1	-6.6	-60.78	-62.17	-1.39	AAG	CCU
pre-mir-411	20	-28.6	-35.2	-6.6	-68.87	-71.41	-2.54	UAG	CCA
pre-mir-1251	10	-20.8	-27.4	-6.6	-49.46	-53	-3.54	UAG	CCA
pre-mir-664a	18	-23.8	-30.4	-6.6				UAG	CCA
pre-mir-379	10	-26.3	-32.9	-6.6				UAG	CCA
pre-mir-503	7	-42.2	-48.8	-6.6				UAG	CCA
pre-mir-1301	52	-45.4	-51.9	-6.5	-58.17	-71.26	-13.09	CAG	CCG
pre-mir-99a	13	-39.3	-45.7	-6.4	-73.94	-75.74	-1.8	AAA	UCU
pre-mir-488	56	-32.2	-38.6	-6.4				AAA	UCU
pre-mir-337	66	-40.5	-46.7	-6.2	-65.75	-66.88	-1.13	UAU	ACA
pre-mir-539	18	-27.9	-34.1	-6.2				UAU	ACA

pre-mir-27a	10	-37.1	-43.3	-6.2				CAG	CCG
pre-mir-140	38	-54.6	-60.8	-6.2				UAU	ACA
pre-mir-340	70	-36.1	-42.2	-6.1				UAC	
pre-mir-513c	20	-34.3	-39.8	-5.5				AAG	UCU
pre-mir-24-2	18	-26	-31.3	-5.3				UAC	GUU
pre-mir-99b	47	-27.6	-30.9	-3.3				AAG	CUU
pre-mir-497	25	-54.1	-56.9	-2.8				CAG	UUA
pre-mir-212	73	-50.1	-52.5	-2.4				AAC	
pre-mir-369	48	-27.8	-29.6	-1.8				AAU	UAU
pre-mir-6503	59	-62.1	-62.9	-0.8				UAG	CAA
pre-mir-605	18	-52.3	-52.4	-0.1				AAA	UUU
pre-mir-641	17	-61.4	-61.5	-0.1				AAA	UUU
pre-let-7e	57	-36.7	-36.7	0	-53.89	-53.65	0.24	UAC	GUA
pre-mir-130a	56	-42	-42	0	-81.57	-81.82	-0.25	CAG	CUA
pre-mir-195	58	-46.6	-46.6	0				UAU	AUA
pre-mir-203b	64	-38.1	-38.1	0				UAA	UAA
pre-mir-27b	66	-49.5	-49.5	0				CAG	UGG
pre-mir-381	52	-35.5	-35.5	0	-57.6	-60.4	-2.8	UAC	GUA

pre-mir-3687	42	-32.9	-32.9	0				CAG	CGG
pre-mir-4731	48	-54.9	-54.9	0	-71.66	-71.58	0.08	AAG	CAU
pre-mir-100	13	-25.7	-25.7	0				AAA	UAU
pre-mir-944	59	-45.3	-45.3	0				UAU	AUA
pre-mir-598	62	-35.9	-35.7	0.2				UAC	GUG
pre-mir-151a	49	-44.4	-44.1	0.3	-81.82	-80.92	0.9	UAG	CUA
pre-mir-641	18	-61.4	-61.1	0.3				AAG	CUU
pre-mir-605	54	-52.3	-52	0.3				AAG	CUU
pre-mir-4662a	8	-35.5	-35.2	0.3				UAG	CUA
pre-mir-376c	48	-25	-24.7	0.3				UAG	CUA
pre-mir-3681	10	-53.1	-52.8	0.3	-62.32	-62.35	-0.03	UAG	CUA
pre-mir-27a	64	-37.1	-36.8	0.3	-68.93	-69.02	-0.09	AAG	CUU
pre-mir-27b	64	-49.5	-49.1	0.4				CAC	GUG
pre-mir-301b	63	-31.2	-30.5	0.7				CAA	CUG
pre-mir-27a	56	-37.1	-36.4	0.7				CAG	CUU
pre-mir-3622a	29	-68.2	-67.5	0.7				CAG	CUG
pre-mir-374b	47	-40.7	-40	0.7				CAG	CUG
pre-mir-214	76	-67	-66.2	0.8				CAG	UUG

pre-mir-421	54	-35.1	-34.3	0.8	-76.79	-76.27	0.52	CAG	UUG
pre-mir-624	23	-54.6	-53.8	0.8				CAG	UUG
pre-mir-589	65	-42	-41.1	0.9				GAA	CUC

Supplemental Table S5B: Two-dimensional structural prediction analysis for pre-miRNAs in the unedited and edited (C-to-U) forms

miRNA	Edited position in pre-mirna	Unedited (MFE in kcal/mol)	Edited (MFE in kcal/mol)	2D-ΔΔG
pre-mir-100	17	-25.7	-30	-4.3
pre-mir-122	25	-45.7	-50	-4.3
pre-mir-491	26	-48	-50.7	-2.7
pre-mir-30d	16	-28.1	-29.2	-1.1
pre-mir-181c	75	-45.2	-46	-0.8
Pre-mir-26b	23	-39.1	-39.4	-0.3
pre-mir-191	25	-46.8	-46.8	0
pre-mir-375	50	-26.8	-26.8	0
pre-mir-5010	31	-43.4	-43.4	0
pre-mir-598	33	-36.1	-36.1	0
pre-mir-99a	24	-39.5	-39.5	0
pre-mir-425	25	-34.5	-33.6	0.9
pre-mir-30a	16	-37.2	-36.3	0.9
pre-mir-330	74	-45	-44	1
pre-mir-99a	29	-39.5	-38.4	1.1
pre-mir-181c	36	-45.2	-43.7	1.5
pre-mir-208a	54	-32.4	-30.8	1.6
pre-mir-23a	47	-33.3	-31.7	1.6
pre-mir-106b	28	-43.2	-41.6	1.6
pre-mir-152	55	-47.2	-45.6	1.6
pre-mir-30e	27	-51.8	-50.1	1.7
pre-mir-769	38	-60.8	-59.1	1.7
pre-mir-200c	52	-30.8	-29.1	1.7

pre-mir-204	43	-42	-40.3	1.7
pre-mir-208b	56	-31.4	-29.6	1.8
pre-mir-425	21	-34.5	-32.7	1.8
pre-mir-323b	60	-40.2	-38.4	1.8
pre-mir-19a	63	-38.7	-36.7	2
pre-mir-342	77	-47.7	-45.7	2
pre-mir-409	23	-35.9	-33.7	2.2
pre-mir-23b	60	-35.5	-33.3	2.2
pre-mir-421	50	-35	-32.8	2.2
pre-mir-598	75	-36.1	-33.9	2.2
pre-mir-99a	25	-39.5	-37.3	2.2
pre-mir-99b	19	-27.6	-25.4	2.2
pre-mir-138-1	25	-58.3	-56	2.3
pre-mir-143	73	-51	-48.7	2.3
pre-mir-22	60	-39.8	-37.5	2.3
pre-mir-451a	27	-43.4	-41.1	2.3
pre-mir-29a	48	-24.6	-22.3	2.3
pre-mir-21	20	-34.6	-32.3	2.3
pre-mir-100	25	-25.7	-23.3	2.4
pre-mir-30a	15	-37.2	-34.8	2.4
pre-mir-34a	36	-47.2	-44.8	2.4
pre-mir-125a	25	-44.2	-41.7	2.5
pre-mir-374b	22	-40.3	-37.7	2.6
pre-mir-451a	26	-43.4	-40.8	2.6
pre-mir-1307	56	-53.8	-51.1	2.7
pre-mir-423	70	-48.8	-46.1	2.7
pre-mir-23b	67	-35.5	-32.8	2.7
pre-mir-30e	26	-51.8	-48.9	2.9
pre-mir-99a	17	-39.5	-36.6	2.9
pre-mir-1296	74	-50.5	-47.5	3
pre-mir-1295a	57	-49.2	-45	4.2
pre-mir-33a	20	-35.6	-31.2	4.4
pre-mir-219a-2	33	-49.3	-44.4	4.9

Supplemental Table S6A: DESeq2 analysis of differential miRNA expression in 5 GBM compared to 6 FC samples

293 miRNAs downregulated in GBM

miRNA name	log2FoldChange	padj
hsa-mir-876-5p	-9.027999076	3.72E-22
hsa-mir-876-3p	-7.866142814	3.44E-16
hsa-mir-873-5p	-7.55774423	9.23E-55
hsa-mir-577	-7.475815624	1.8E-17
hsa-mir-873-3p	-7.395570204	1.1E-24
hsa-mir-490-5p	-7.244154819	2.03E-14
hsa-mir-490-3p	-7.224225136	2.97E-28
hsa-mir-539-5p	-6.832768514	2.19E-33
hsa-mir-139-3p	-6.580859231	1.94E-44
hsa-mir-1298-3p	-6.506883138	3.16E-19
hsa-mir-1264	-6.41395177	3.23E-13
hsa-mir-219a-2-5p	-6.403732865	2.23E-09
hsa-mir-3943	-6.154899291	1.09E-24
hsa-mir-383-3p	-6.038617904	9.77E-10
hsa-mir-433-3p	-6.02225047	1.58E-20
hsa-mir-1298-5p	-5.863129805	1.81E-12
hsa-mir-6841-5p	-5.862070992	1.34E-08
hsa-mir-203b-3p	-5.843248294	8.98E-13
hsa-mir-522-3p	-5.680330609	5.32E-09
hsa-mir-338-3p	-5.615122346	1.98E-15
hsa-mir-1224-5p	-5.587571546	5.8E-30
hsa-mir-1912	-5.47901767	3.11E-08
hsa-mir-137	-5.454686882	4.17E-26
hsa-mir-139-5p	-5.446847498	1.24E-52
hsa-mir-412-5p	-5.288113623	9.24E-05
hsa-mir-3663-3p	-5.101641283	1.79E-06
hsa-mir-6794-3p	-4.907720004	6.67E-08
hsa-mir-1911-5p	-4.885086056	1.24E-05
hsa-mir-203a-5p	-4.646557941	7.5E-10
hsa-mir-1250-5p	-4.602035165	6.41E-09
hsa-mir-504-3p	-4.454854114	7.46E-05
hsa-mir-770-5p	-4.416929605	1.57E-12
hsa-mir-338-5p	-4.383844333	2.01E-08
hsa-mir-504-5p	-4.359551508	2.53E-09
hsa-mir-128-1-5p	-4.311099591	1.16E-16

hsa-mir-6881-3p	-4.237147518	0.000118
hsa-mir-889-3p	-4.186715941	1.77E-12
hsa-mir-7158-5p	-4.130890233	0.001044
hsa-mir-4501	-4.090076139	0.000311
hsa-mir-1249-5p	-4.06296066	3.93E-21
hsa-mir-383-5p	-4.034646639	8.32E-06
hsa-mir-1286	-4.003384938	1.45E-10
hsa-mir-431-3p	-3.928030672	2.34E-07
hsa-mir-767-3p	-3.914104949	5.71E-05
hsa-mir-767-5p	-3.910151623	1.93E-12
hsa-mir-6507-5p	-3.883593703	8.05E-06
hsa-mir-1252-3p	-3.875245681	0.002617
hsa-mir-1252-5p	-3.857867881	1.34E-08
hsa-mir-518e-3p	-3.8269765	0.001732
hsa-mir-668-3p	-3.795620496	4.45E-07
hsa-mir-5680	-3.781870403	7.6E-10
hsa-mir-433-5p	-3.774690548	5.98E-11
hsa-mir-3938	-3.77007807	5.85E-09
hsa-mir-1296-5p	-3.764380953	2.73E-15
hsa-mir-410-3p	-3.709754737	2.64E-09
hsa-mir-656-5p	-3.687994202	0.000286
hsa-mir-184	-3.683985736	1.47E-10
hsa-mir-3200-3p	-3.673893274	1.49E-24
hsa-mir-448	-3.673736952	0.002007
hsa-mir-323b-3p	-3.669751625	6.03E-07
hsa-mir-124-1-3p	-3.650857592	0.003464
hsa-mir-128-2-5p	-3.632501562	5.04E-07
hsa-mir-7-2-5p	-3.619477776	0.009105
hsa-mir-4671-3p	-3.608072907	3.63E-06
hsa-mir-154-3p	-3.576495578	1.61E-11
hsa-mir-874-3p	-3.547345757	1.98E-15
hsa-mir-323a-5p	-3.539278754	4.16E-07
hsa-mir-330-5p	-3.532299146	7.15E-38
hsa-mir-432-3p	-3.531544018	1.4E-10
hsa-mir-598-5p	-3.506106497	2.74E-06
hsa-mir-541-3p	-3.500543128	1.56E-05
hsa-mir-5187-5p	-3.498285172	0.000133
hsa-mir-370-5p	-3.467542898	3.61E-08
hsa-mir-496	-3.46215769	1.06E-08
hsa-mir-7-1-5p	-3.452402411	0.002819
hsa-mir-487b-5p	-3.441981484	9.31E-09

hsa-mir-885-5p	-3.395950492	0.010855
hsa-mir-642a-3p	-3.383086068	0.017726
hsa-mir-330-3p	-3.346447938	2.77E-32
hsa-mir-7855-5p	-3.291415086	0.016843
hsa-mir-211-5p	-3.273830018	0.009147
hsa-mir-4446-5p	-3.265372565	0.006977
hsa-mir-29c-5p	-3.261006517	2.48E-07
hsa-mir-1231	-3.253392619	0.001271
hsa-mir-758-5p	-3.238841775	3.27E-06
hsa-mir-889-5p	-3.233059058	5.95E-07
hsa-mir-6877-3p	-3.214982901	0.000855
hsa-mir-513c-5p	-3.21231178	0.013015
hsa-mir-2682-5p	-3.210508548	2.55E-06
hsa-mir-518b	-3.193923675	2.1E-05
hsa-mir-1277-3p	-3.173881672	0.003292
hsa-mir-495-3p	-3.167733222	1.47E-07
hsa-mir-4743-5p	-3.166577793	0.00264
hsa-mir-769-3p	-3.163844413	2.21E-20
hsa-mir-485-3p	-3.137220851	7.37E-06
hsa-mir-7-3-5p	-3.13534517	0.040057
hsa-mir-487a-5p	-3.110103831	8.08E-06
hsa-mir-4729	-3.109277334	0.029904
hsa-mir-487b-3p	-3.100355228	7.48E-07
hsa-mir-129-2-5p	-3.099399178	0.023068
hsa-mir-584	-3.097641558	2.68E-05
hsa-mir-519a-1-5p	-3.090082747	0.035621
hsa-mir-769-5p	-3.085194037	3.09E-15
hsa-mir-4446-3p	-3.072497479	7.91E-07
hsa-mir-127-3p	-3.058947893	5.9E-10
hsa-mir-4671-5p	-3.057150103	0.007881
hsa-mir-656-3p	-3.054632126	1.5E-08
hsa-mir-323a-3p	-3.043070689	8.05E-06
hsa-mir-491-5p	-3.042641844	1.71E-08
hsa-mir-1197	-3.014903827	1.83E-06
hsa-mir-487a-3p	-2.987337812	8.71E-06
hsa-mir-1179	-2.943466048	3.01E-10
hsa-mir-299-5p	-2.942612197	5.49E-06
hsa-mir-380-5p	-2.930504672	0.000221
hsa-mir-410-5p	-2.912962	0.000585
hsa-mir-3663-5p	-2.883542306	0.028082
hsa-mir-508-3p	-2.871576643	5.41E-06

hsa-mir-132-3p	-2.852509788	7.33E-09
hsa-mir-132-5p	-2.818081821	2.26E-07
hsa-mir-5088-3p	-2.791827875	0.075778
hsa-mir-346	-2.787185325	1.98E-09
hsa-mir-642a-5p	-2.782965153	0.00476
hsa-mir-3167	-2.76595738	0.029904
hsa-mir-411-5p	-2.760367535	6.26E-07
hsa-mir-491-3p	-2.756725365	1.81E-07
hsa-mir-539-3p	-2.749383177	1.06E-06
hsa-mir-670-5p	-2.73996814	0.028637
hsa-mir-935	-2.726220732	6.09E-08
hsa-mir-518c-3p	-2.71603013	0.060933
hsa-mir-2682-3p	-2.70481452	0.000423
hsa-mir-127-5p	-2.673134487	6.57E-06
hsa-mir-6516-5p	-2.6708607	7.73E-07
hsa-mir-6786-3p	-2.670552891	1.5E-05
hsa-mir-377-5p	-2.660617146	8.08E-06
hsa-mir-655-3p	-2.65734661	7.52E-06
hsa-mir-519d-3p	-2.647534885	0.082068
hsa-mir-6872-3p	-2.634381436	0.034351
hsa-mir-582-5p	-2.613229319	5.48E-08
hsa-mir-302c-3p	-2.608460811	0.073792
hsa-mir-1296-3p	-2.603736856	1.56E-06
hsa-mir-4701-5p	-2.600337738	0.004516
hsa-mir-1225-5p	-2.599297458	0.013614
hsa-mir-7109-3p	-2.59285412	0.033366
hsa-mir-5191	-2.578311966	0.053953
hsa-mir-362-5p	-2.576666168	0.039036
hsa-mir-3529-5p	-2.567298608	0.092244
hsa-mir-509-3-5p	-2.549918861	0.061848
hsa-mir-4520a-3p	-2.549552084	0.037649
hsa-mir-6865-3p	-2.544621201	0.001565
hsa-mir-2681-5p	-2.518832439	0.008974
hsa-mir-33a-5p	-2.517101374	7.62E-07
hsa-mir-744-3p	-2.505605422	4.82E-18
hsa-mir-5008-3p	-2.48155264	0.047192
hsa-mir-670-3p	-2.467604642	0.000126
hsa-mir-136-3p	-2.451247833	9.93E-06
hsa-mir-582-3p	-2.450292708	7.65E-08
hsa-mir-101-1-3p	-2.440922695	0.013054
hsa-mir-381-5p	-2.429753819	0.000353

hsa-mir-4525	-2.427867882	0.059995
hsa-mir-4640-5p	-2.413302242	0.061848
hsa-mir-5006-3p	-2.406337934	0.001619
hsa-mir-425-5p	-2.401501245	0.002729
hsa-mir-7156-5p	-2.388373727	0.013614
hsa-mir-33a-3p	-2.364777042	5.51E-05
hsa-mir-29b-2-5p	-2.357209003	1.21E-07
hsa-mir-381-3p	-2.350949709	2.47E-05
hsa-mir-4641	-2.304535286	0.055775
hsa-mir-134-3p	-2.29418199	6.58E-05
hsa-mir-382-3p	-2.290731312	6.35E-05
hsa-mir-149-5p	-2.289317667	5.01E-08
hsa-mir-885-3p	-2.264641621	0.00612
hsa-mir-495-5p	-2.250987556	0.00191
hsa-mir-4780	-2.243435477	0.012473
hsa-mir-3120-3p	-2.235547955	0.095213
hsa-mir-5699-5p	-2.229017588	0.000174
hsa-mir-412-3p	-2.218741033	0.022874
hsa-mir-6810-5p	-2.212331027	0.04903
hsa-mir-6860	-2.210798909	0.073666
hsa-mir-485-5p	-2.205354059	0.001933
hsa-mir-379-3p	-2.18505952	0.000133
hsa-mir-138-1-5p	-2.184599158	0.000105
hsa-mir-766-3p	-2.158988307	2.02E-06
hsa-mir-543	-2.143140595	0.002124
hsa-mir-342-3p	-2.139419944	2.93E-08
hsa-mir-4798-5p	-2.117883975	0.022874
hsa-mir-499a-5p	-2.108692418	3.91E-07
hsa-mir-1343-3p	-2.096397768	0.000315
hsa-mir-432-5p	-2.053713019	0.002422
hsa-mir-411-3p	-2.048473082	0.000258
hsa-mir-6508-3p	-2.043216733	0.003935
hsa-mir-375	-2.030774564	0.012105
hsa-mir-382-5p	-2.002183284	0.000348
hsa-mir-6864-5p	-1.999660068	0.016993
hsa-mir-598-3p	-1.989939136	0.000127
hsa-mir-1909-5p	-1.98808198	0.044071
hsa-mir-6735-5p	-1.959591804	0.018056
hsa-mir-377-3p	-1.954478804	0.0004
hsa-mir-585-5p	-1.926155793	0.090046
hsa-mir-4515	-1.906382764	0.07512

hsa-mir-628-5p	-1.906352744	4.9E-12
hsa-mir-874-5p	-1.895946432	0.000922
hsa-mir-33b-5p	-1.886267091	0.028209
hsa-mir-2467-5p	-1.859650121	2.67E-08
hsa-mir-655-5p	-1.846528295	0.055746
hsa-mir-6733-5p	-1.77024162	0.002211
hsa-mir-103a-2	-1.76880401	0.000195
hsa-mir-190a-5p	-1.767451897	1.35E-05
hsa-mir-154-5p	-1.75864733	0.001917
hsa-mir-299-3p	-1.754898637	0.010141
hsa-mir-758-3p	-1.750002282	0.007901
hsa-mir-136-5p	-1.747307295	0.001618
hsa-mir-30a-5p	-1.745540787	4.04E-05
hsa-mir-3617-5p	-1.721374638	0.019888
hsa-mir-541-5p	-1.716807732	0.004886
hsa-mir-760	-1.712902642	0.000189
hsa-mir-6734-5p	-1.711751982	0.050271
hsa-mir-212-5p	-1.709278428	0.000739
hsa-mir-212-3p	-1.705736951	0.001025
hsa-mir-940	-1.69458573	6.59E-05
hsa-mir-5699-3p	-1.689590731	0.08281
hsa-mir-744-5p	-1.677048759	7.94E-09
hsa-mir-30d-5p	-1.671631382	6.92E-12
hsa-mir-153-2-5p	-1.670621956	0.004137
hsa-mir-3177-5p	-1.655186843	0.043964
hsa-mir-5010-3p	-1.645701114	1.55E-05
hsa-mir-138-2-5p	-1.60806718	0.004588
hsa-mir-5683	-1.592020257	0.0551
hsa-mir-369-3p	-1.581474105	0.003454
hsa-mir-29c-3p	-1.581463722	0.000767
hsa-mir-676-3p	-1.574816458	0.002155
hsa-mir-3139	-1.566535951	0.077656
hsa-mir-107	-1.559040463	0.000258
hsa-mir-4786-5p	-1.542184827	0.00915
hsa-mir-1269a	-1.527643812	0.056416
hsa-mir-585-3p	-1.525438804	0.067922
hsa-mir-6882-5p	-1.518746997	0.006954
hsa-mir-3200-5p	-1.511047255	0.000258
hsa-mir-6721-5p	-1.49689083	0.017906
hsa-mir-6816-3p	-1.479939869	0.090402
hsa-mir-326	-1.430406394	0.002356

hsa-mir-134-5p	-1.421278206	0.019292
hsa-mir-98-5p	-1.400678868	3.13E-06
hsa-mir-4738-3p	-1.389175532	0.034906
hsa-mir-374a-5p	-1.382680934	0.000961
hsa-mir-6516-3p	-1.377674918	0.008965
hsa-mir-2681-3p	-1.377630972	0.059019
hsa-mir-95-3p	-1.366505589	0.000902
hsa-mir-409-3p	-1.340539473	0.044186
hsa-mir-191-3p	-1.303109552	5.6E-05
hsa-mir-4767	-1.301720541	0.013482
hsa-mir-29a-5p	-1.291343236	0.001383
hsa-mir-30e-3p	-1.271006371	9.03E-06
hsa-mir-204-5p	-1.265013518	0.056829
hsa-mir-191-5p	-1.259840601	0.001238
hsa-mir-340-3p	-1.258716736	0.001383
hsa-mir-488-3p	-1.237419566	0.02955
hsa-mir-3117-3p	-1.218307837	0.005913
hsa-mir-4787-3p	-1.207100624	1.56E-05
hsa-mir-3064-3p	-1.187295875	0.056416
hsa-mir-340-5p	-1.174749646	0.01814
hsa-mir-181a-1-3p	-1.169565944	0.027
hsa-mir-361-5p	-1.166473567	0.000133
hsa-mir-369-5p	-1.158911456	0.05748
hsa-mir-659-5p	-1.13213103	0.03234
hsa-mir-1307-5p	-1.127079307	0.006361
hsa-mir-30b-3p	-1.090164017	0.075778
hsa-mir-337-5p	-1.08571573	0.02589
hsa-mir-30e-5p	-1.077129538	0.003134
hsa-mir-324-5p	-1.070201475	0.000344
hsa-mir-101-1-5p	-1.06776533	0.011192
hsa-mir-376a-1-5p	-1.067606174	0.093089
hsa-mir-101-2	-1.055011141	0.07808
hsa-mir-30b-5p	-1.050026261	0.004179
hsa-mir-1306-5p	-1.027276897	0.003547
hsa-mir-190a-3p	-1.02246198	0.036136
hsa-mir-1180-3p	-1.001321632	0.037912
hsa-mir-2110	-0.993507271	0.017946
hsa-mir-628-3p	-0.991482387	0.000739
hsa-mir-26a-2-5p	-0.96328031	0.049949
hsa-mir-181d	-0.961788964	0.052313
hsa-mir-181c-5p	-0.923042485	0.037912

hsa-mir-328-3p	-0.913621809	0.000757
hsa-mir-331-5p	-0.899587113	0.005327
hsa-let-7g-3p	-0.894303816	0.017906
hsa-mir-1301-3p	-0.887754992	0.016155
hsa-mir-146b-5p	-0.882802256	0.07608
hsa-let-7d-3p	-0.819888083	0.008053
hsa-mir-425-3p	-0.748013309	0.01814
hsa-let-7d-5p	-0.669784979	0.063803
hsa-mir-29a-3p	-0.664546817	0.004525
hsa-mir-30a-3p	-0.656755167	0.063878
hsa-mir-1307-3p	-0.614239589	0.004773

263 miRNAs upregulated in GBM

miRNA name	log2FoldChange	padj
hsa-mir-615-3p	8.754048903	7.33481E-13
hsa-mir-199a-1-3p	7.278434937	1.33786E-12
hsa-mir-10a-3p	7.146310697	1.44779E-10
hsa-mir-10b-3p	6.457874774	8.69112E-15
hsa-mir-892b	5.685030773	5.33793E-07
hsa-mir-888-5p	5.64151116	1.71611E-13
hsa-mir-1908-5p	5.277463042	2.79467E-07
hsa-mir-891a-5p	5.264511879	6.6424E-13
hsa-mir-199b-5p	5.096158265	4.67831E-12
hsa-mir-6503-5p	5.091795275	2.31595E-10
hsa-mir-6503-3p	5.072145474	1.0897E-13
hsa-mir-21-5p	4.924199432	1.35761E-58
hsa-mir-3679-5p	4.898773849	4.32805E-06
hsa-mir-196b-3p	4.839208998	0.00013029
hsa-mir-4466	4.674129004	0.000241292
hsa-mir-891b	4.668171373	7.48083E-07
hsa-mir-96-5p	4.479282212	2.09907E-10
hsa-mir-503-3p	4.428458358	0.000117111
hsa-mir-196a-2-5p	4.400791494	0.004995367
hsa-mir-3591-5p	4.362894094	0.005196511
hsa-mir-892c-3p	4.349275301	0.001136576
hsa-mir-190b	4.275031103	3.7396E-08
hsa-mir-182-3p	4.208326332	0.008303119
hsa-mir-4474-3p	4.188739768	0.002078837
hsa-mir-3619-5p	4.17986215	0.001014962
hsa-mir-892a	4.160232618	1.20529E-07

hsa-mir-21-3p	4.14795477	5.08741E-39
hsa-mir-4423-3p	4.145887092	0.000146342
hsa-mir-196a-2-3p	4.140094459	0.055100072
hsa-mir-27a-5p	4.134179792	5.67205E-15
hsa-mir-10b-5p	4.115891647	1.11634E-06
hsa-mir-4772-5p	3.996637499	0.000399774
hsa-mir-4772-3p	3.971861044	0.001689109
hsa-mir-6125	3.968236377	0.001401232
hsa-mir-3659	3.896989811	0.000516322
hsa-mir-3143	3.829523148	3.1582E-06
hsa-mir-10a-5p	3.798454248	1.98924E-07
hsa-mir-615-5p	3.778495813	0.018690691
hsa-mir-193a-3p	3.742732904	4.45084E-08
hsa-mir-5001-5p	3.608307947	0.006485676
hsa-mir-4741	3.561303275	0.000654687
hsa-mir-888-3p	3.532203383	0.029904065
hsa-mir-144-5p	3.496240277	9.04795E-12
hsa-mir-3651	3.479960604	0.009337371
hsa-mir-450b-5p	3.461692974	1.18644E-12
hsa-mir-4649-5p	3.343550446	0.018718978
hsa-mir-450a-1-5p	3.33632797	0.057479868
hsa-mir-142-3p	3.326423838	1.52106E-11
hsa-mir-155-5p	3.273055179	1.75099E-26
hsa-mir-210-3p	3.271111592	1.86503E-17
hsa-mir-449c-5p	3.26979318	3.11237E-05
hsa-mir-2114-3p	3.255872283	2.76105E-05
hsa-mir-599	3.236100507	0.030483378
hsa-mir-199a-2-3p	3.222106311	0.006935055
hsa-mir-296-3p	3.185849773	3.88209E-08
hsa-mir-3911	3.179402597	0.040600554
hsa-mir-424-3p	3.177628566	5.3982E-12
hsa-mir-224-5p	3.171293254	4.14828E-12
hsa-mir-142-5p	3.146775682	3.08782E-15
hsa-mir-449b-5p	3.141731108	0.001270347
hsa-mir-4711-5p	3.09243447	0.076581855
hsa-mir-6727-5p	3.090626717	0.049358159
hsa-mir-214-5p	3.068353757	2.47919E-05
hsa-mir-92b-5p	3.054241367	7.14583E-15
hsa-mir-675-5p	3.02831834	0.012389814
hsa-mir-24-2-5p	3.021157136	2.98367E-24
hsa-mir-3937	3.008890464	0.039423898

hsa-mir-4777-3p	2.966605551	0.024576343
hsa-mir-4521	2.965270252	0.000520051
hsa-mir-2114-5p	2.95281094	0.000199197
hsa-mir-3913-1-5p	2.861445289	0.031400809
hsa-mir-4516	2.844834588	0.039599925
hsa-mir-4649-3p	2.839448256	0.00171963
hsa-mir-223-5p	2.828245161	2.85138E-07
hsa-mir-493-3p	2.814567186	6.93097E-06
hsa-mir-451a	2.757845052	2.55068E-07
hsa-mir-7976	2.747953	0.00183087
hsa-mir-219a-1-5p	2.709824515	2.51765E-05
hsa-mir-4648	2.709096553	0.012021171
hsa-mir-503-5p	2.708598594	1.96795E-08
hsa-mir-365b-5p	2.704165682	1.53918E-06
hsa-mir-144-3p	2.700448641	1.0377E-09
hsa-mir-135a-1-3p	2.688539952	0.000581361
hsa-mir-3129-3p	2.684505484	1.50868E-06
hsa-mir-3194-5p	2.670137503	1.11448E-05
hsa-mir-92b-3p	2.632405299	3.29693E-08
hsa-mir-3609	2.586632504	0.001977073
hsa-mir-493-5p	2.574552644	0.000205031
hsa-mir-4433b-5p	2.539754656	0.002210715
hsa-mir-23a-3p	2.5388906	7.62861E-17
hsa-mir-7705	2.532390789	6.03021E-07
hsa-mir-23a-5p	2.52011803	0.008997858
hsa-mir-605-3p	2.518348339	0.002210715
hsa-mir-3614-5p	2.515499107	0.001933278
hsa-mir-4732-5p	2.458219615	0.007288501
hsa-mir-217	2.456142127	0.008053021
hsa-mir-135b-3p	2.455922234	0.000510669
hsa-mir-210-5p	2.449390945	8.39814E-12
hsa-mir-150-5p	2.429252291	0.00012549
hsa-mir-195-3p	2.422804858	7.47803E-11
hsa-mir-15b-3p	2.403395714	3.46928E-07
hsa-mir-4746-5p	2.400142524	8.48557E-07
hsa-mir-500b-3p	2.390775457	0.009232485
hsa-mir-4709*	2.368912469	0.07081071
hsa-mir-6852-5p	2.362003617	6.13655E-07
hsa-mir-216a-3p	2.337857402	0.048264568
hsa-mir-4802-5p	2.323250257	0.096302512
hsa-mir-4725-5p	2.28173713	0.061848407

hsa-mir-2115-3p	2.276995301	0.008303119
hsa-mir-455-3p	2.275312376	1.03249E-11
hsa-mir-92a-1-5p	2.261980851	0.00156495
hsa-mir-16-2-5p	2.254277983	0.017747245
hsa-mir-1262	2.250316908	9.54445E-06
hsa-mir-4687-3p	2.247857614	0.033345187
hsa-mir-942-5p	2.246838455	1.88035E-06
hsa-mir-150-3p	2.244999495	0.000480151
hsa-mir-4690-3p	2.244712179	0.035185119
hsa-mir-3194-3p	2.241776888	0.016215761
hsa-mir-548e-3p	2.241753454	2.70668E-06
hsa-mir-222-5p	2.237491594	3.36722E-05
hsa-mir-6502-5p	2.221548508	0.003354854
hsa-mir-34c-5p	2.195247487	0.018993208
hsa-mir-214-3p	2.157939156	0.003926674
hsa-mir-6875-5p	2.15267133	0.029407208
hsa-mir-152-5p	2.129099947	0.025889587
hsa-mir-3942-5p	2.108877204	0.001392274
hsa-mir-16-1-5p	2.106970919	0.013282638
hsa-mir-371b-5p	2.104178462	0.009194738
hsa-mir-25-5p	2.102524939	2.76105E-05
hsa-mir-3616-5p	2.095785512	0.09956403
hsa-mir-188-5p	2.087037643	4.45084E-08
hsa-mir-4423-5p	2.082804093	0.016155072
hsa-mir-223-3p	2.061932626	4.92894E-06
hsa-mir-629-3p	2.029797519	0.000391246
hsa-mir-4642	2.010932108	0.099476534
hsa-mir-3653-5p	2.007859671	0.02306824
hsa-mir-143-5p	2.001456102	1.36031E-05
hsa-mir-497-5p	1.995953066	1.93049E-11
hsa-mir-502-3p	1.989885621	0.000160093
hsa-mir-296-5p	1.967881172	6.63637E-05
hsa-mir-497-3p	1.958148398	1.28153E-06
hsa-mir-1276	1.947653067	0.003323155
hsa-mir-216a-5p	1.939586176	0.063599166
hsa-mir-106b-3p	1.932946808	8.54236E-09
hsa-mir-27a-3p	1.903977084	3.83095E-09
hsa-mir-501-3p	1.894355052	0.001330594
hsa-mir-1228-3p	1.891485449	5.59289E-05
hsa-mir-216b-5p	1.885297468	0.056335796
hsa-mir-6513-3p	1.847503864	0.012105259

hsa-mir-6501-5p	1.847010963	0.063599166
hsa-mir-556-5p	1.832109357	0.006976922
hsa-mir-937-3p	1.824681552	3.62264E-05
hsa-mir-224-3p	1.817929762	0.008611713
hsa-mir-542-5p	1.816107313	0.001933278
hsa-mir-2115-5p	1.810327304	0.099476534
hsa-mir-15b-5p	1.78121826	3.11237E-05
hsa-mir-455-5p	1.780233046	4.33421E-09
hsa-mir-1228-5p	1.773954561	0.005055697
hsa-mir-3150b-3p	1.753801238	0.094679612
hsa-mir-4326	1.736010798	0.001213291
hsa-mir-339-5p	1.732079938	0.007835861
hsa-mir-4647	1.723893806	0.079785879
hsa-mir-4803	1.715773616	0.029165869
hsa-mir-3176	1.707509791	5.89564E-10
hsa-mir-20b-3p	1.686861987	0.091149203
hsa-mir-186-3p	1.675713248	0.021746815
hsa-mir-576-5p	1.674666718	0.037357891
hsa-mir-627-5p	1.667872178	8.88101E-06
hsa-mir-219b-5p	1.656320007	0.086192464
hsa-mir-3661	1.646589478	0.000084878
hsa-mir-548ba	1.634435297	0.006485676
hsa-mir-423-3p	1.625382387	1.33776E-08
hsa-mir-4709-5p	1.619964325	0.046429831
hsa-mir-486-2-5p	1.609439646	0.020890656
hsa-mir-3622a-3p	1.59353813	0.019757722
hsa-mir-93-3p	1.584648402	0.000211055
hsa-mir-4661-5p	1.576343026	0.021601294
hsa-mir-4758-3p	1.552988829	0.051653419
hsa-mir-551b-3p	1.551497473	2.12406E-05
hsa-mir-3129-5p	1.551433164	0.055878258
hsa-mir-3187-3p	1.549770976	0.005239952
hsa-mir-93-5p	1.543707132	0.0000526
hsa-mir-193a-5p	1.543182556	0.004772616
hsa-mir-320a	1.540282257	3.22131E-09
hsa-mir-589-3p	1.52122931	2.75035E-05
hsa-mir-424-5p	1.521117166	0.001070467
hsa-mir-532-3p	1.515499298	1.47502E-06
hsa-mir-34a-3p	1.512032164	0.012734017
hsa-mir-3065-3p	1.501433396	0.017946399
hsa-mir-542-3p	1.500372426	0.005938413

hsa-mir-34c-3p	1.486174164	0.074491592
hsa-mir-3136-5p	1.471743995	0.085133398
hsa-mir-148a-3p	1.470462766	0.016369034
hsa-mir-1226-5p	1.446347582	0.046429831
hsa-mir-618	1.444598433	0.006874383
hsa-mir-19a-3p	1.434596251	0.021214217
hsa-mir-551b-5p	1.432443378	0.061355946
hsa-mir-25-3p	1.430502531	0.0000526
hsa-mir-3940-3p	1.429672353	0.011655485
hsa-mir-3151-5p	1.427140061	0.056416404
hsa-mir-152-3p	1.422680986	0.003218852
hsa-mir-431-5p	1.408593213	0.089196046
hsa-mir-27b-5p	1.406147046	0.008997858
hsa-mir-4636	1.363820714	0.060564085
hsa-mir-449a	1.318326117	0.053007051
hsa-mir-548al	1.31047952	0.002675554
hsa-mir-548k	1.290700927	0.000157173
hsa-mir-200b-3p	1.285793008	0.00717205
hsa-mir-215-5p	1.279410708	0.085408907
hsa-mir-28-3p	1.278641522	4.36725E-05
hsa-mir-452-3p	1.270316379	0.019707535
hsa-mir-3691-5p	1.265364844	0.018836615
hsa-mir-4775-5p	1.258660424	8.71995E-05
hsa-mir-556-3p	1.252747617	0.093602432
hsa-mir-423-5p	1.243219683	5.91883E-05
hsa-mir-653-3p	1.240114763	0.071154201
hsa-mir-624-3p	1.238998527	0.099122726
hsa-mir-130a-5p	1.234627742	0.069641811
hsa-mir-452-5p	1.233628073	0.033230581
hsa-mir-28-5p	1.233340763	0.000169527
hsa-mir-362-3p	1.227750847	0.002155649
hsa-mir-26b-3p	1.227432453	0.004995367
hsa-mir-3922-3p	1.217309978	0.023943344
hsa-mir-939-5p	1.216907108	0.073666091
hsa-mir-532-5p	1.212322554	4.35778E-05
hsa-let-7c-3p	1.210491583	1.54707E-05
hsa-mir-502-5p	1.181445839	0.002790689
hsa-mir-208b-3p	1.17014128	0.013603999
hsa-mir-148a-5p	1.164473655	0.03761744
hsa-mir-130b-3p	1.140796655	0.001384145
hsa-mir-1266-5p	1.133151461	0.033390675

hsa-mir-335-5p	1.12282491	0.064411152
hsa-mir-3174	1.118614061	0.047868847
hsa-mir-130a-3p	1.117167069	0.000917078
hsa-mir-106b-5p	1.086925628	0.003922355
hsa-mir-454-5p	1.078507243	0.015760122
hsa-mir-301a-5p	1.078385494	0.003174819
hsa-mir-454-3p	1.061396726	0.001845931
hsa-mir-193b-3p	1.01014283	0.00467807
hsa-mir-2277-5p	0.983096271	9.85371E-05
hsa-mir-505-3p	0.975746312	0.014272899
hsa-mir-548ah-5p	0.969394154	0.079785879
hsa-mir-17-3p	0.955899372	0.04626329
hsa-mir-4645-3p	0.939969512	0.011236671
hsa-mir-193b-5p	0.938294779	0.034350891
hsa-mir-671-5p	0.925149786	0.094189085
hsa-mir-500a-3p	0.8874222	0.000931657
hsa-mir-548b-3p	0.87527052	0.056416404
hsa-mir-18a-3p	0.871166969	0.086204615
hsa-mir-148b-5p	0.869651834	0.000080927
hsa-mir-5001-3p	0.861165509	0.056416404
hsa-let-7i-5p	0.853143385	0.007931787
hsa-mir-580-3p	0.839344317	0.035575895
hsa-mir-195-5p	0.821324386	0.009073724
hsa-mir-345-5p	0.773474931	0.012021171
hsa-mir-3613-5p	0.745181282	0.039036124
hsa-mir-652-5p	0.739209013	0.055100072
hsa-mir-141-3p	0.736617728	0.073443627
hsa-mir-501-5p	0.700181879	0.023355635
hsa-mir-125a-3p	0.663627429	0.058260766
hsa-mir-22-3p	0.63677942	0.035621221
hsa-mir-660-5p	0.608362845	0.06380315
hsa-mir-99a-3p	0.603292795	0.008390121

Supplemental Table S6B: DESeq2 analysis of differential miRNA expression in 5 GBM compared to 6 CC samples

<u>268 miRNAs downregulated in GBM</u>

miRNA	log2FoldChange	padj
hsa-mir-577	-9.61670562	1.03E-45
hsa-mir-219a-2-5p	-8.777002667	1.53E-32
hsa-mir-3663-3p	-7.724301873	8.2E-07
hsa-mir-876-5p	-7.272390415	3.08E-13

hsa-mir-7158-5p	-7.049953415	1.79E-05
hsa-mir-6828-5p	-7.025756938	1.12E-05
hsa-mir-338-3p	-6.963040557	1.54E-56
hsa-mir-490-5p	-6.625747233	5.05E-05
hsa-mir-873-5p	-6.332942951	1.13E-46
hsa-mir-873-3p	-6.20798061	2.03E-19
hsa-mir-1250-5p	-5.919995165	1.56E-27
hsa-mir-338-5p	-5.820935941	3.19E-18
hsa-mir-5196-3p	-5.778692526	0.000676
hsa-mir-1911-5p	-5.535279644	6.99E-06
hsa-mir-383-3p	-5.517894051	0.002361
hsa-mir-6881-3p	-5.50729916	1.82E-05
hsa-mir-876-3p	-5.497097178	2.13E-05
hsa-mir-1298-5p	-5.476330799	1.7E-08
hsa-mir-4729	-5.380335112	4.31E-05
hsa-mir-490-3p	-5.379841066	1.03E-15
hsa-mir-539-5p	-5.363605372	2.89E-40
hsa-mir-6841-5p	-5.276088158	0.003184
hsa-mir-3663-5p	-5.212751019	0.003218
hsa-mir-513c-5p	-5.184152194	1.86E-06
hsa-mir-642a-3p	-5.09888222	7.04E-07
hsa-mir-1264	-5.082670339	1.25E-07
hsa-mir-184	-5.024295966	3.61E-14
hsa-mir-1298-3p	-4.910220367	6.94E-08
hsa-mir-657	-4.88936164	0.006173
hsa-mir-3173-3p	-4.881097625	0.008279
hsa-mir-3943	-4.852442801	2.52E-19
hsa-mir-584-5p	-4.796011039	2.84E-37
hsa-mir-1249-5p	-4.671137232	1.06E-26
hsa-mir-6872-5p	-4.590377901	0.019394
hsa-mir-1912	-4.590127095	4.37E-05
hsa-mir-6827-5p	-4.578694358	0.01265
hsa-mir-139-3p	-4.485234749	4.95E-31
hsa-mir-6507-5p	-4.441945937	8.44E-07
hsa-mir-181a-1-5p	-4.431096109	0.017091
hsa-mir-412-5p	-4.408575499	0.041612
hsa-mir-1224-5p	-4.271253316	2.1E-21
hsa-mir-211-5p	-4.268718472	0.000676
hsa-mir-6872-3p	-4.255413207	0.005573
hsa-mir-522-3p	-4.244170635	0.002691
hsa-mir-3612	-4.227673191	0.03405

hsa-mir-642a-5p	-4.160199936	3.63E-13
hsa-mir-433-3p	-4.128069789	3.26E-14
hsa-mir-139-5p	-4.118897024	1.87E-34
hsa-mir-330-5p	-4.077495221	1.03E-57
hsa-mir-6876-5p	-4.070226653	0.036058
hsa-mir-4501	-3.998571356	0.050103
hsa-mir-3167	-3.979225167	0.000854
hsa-mir-1277-3p	-3.977243845	1.94E-05
hsa-mir-203b-3p	-3.947752383	5.22E-06
hsa-mir-330-3p	-3.874472796	5.09E-33
hsa-mir-584-3p	-3.83563945	2.11E-06
hsa-mir-5008-3p	-3.799795634	0.010928
hsa-mir-4780	-3.767562182	8.56E-08
hsa-mir-302c-3p	-3.762719452	0.062893
hsa-mir-4525	-3.750513162	0.002472
hsa-mir-3120-5p	-3.656693998	0.078051
hsa-mir-5191	-3.610237423	0.00039
hsa-mir-520a-3p	-3.562556132	0.07693
hsa-mir-181a-2-5p	-3.554387844	0.000275
hsa-mir-3139	-3.542805141	9.87E-12
hsa-mir-3200-3p	-3.483306634	1.6E-25
hsa-mir-3120-3p	-3.472196322	0.004703
hsa-mir-5699-5p	-3.441510591	3.37E-25
hsa-mir-1250-3p	-3.422347289	0.017622
hsa-mir-4640-5p	-3.406743563	0.006774
hsa-mir-203a-5p	-3.373768643	7.96E-06
hsa-mir-6794-3p	-3.357270536	0.008827
hsa-mir-874-3p	-3.335611129	1.27E-13
hsa-mir-1286	-3.321039123	8.64E-08
hsa-mir-4446-5p	-3.320861564	0.031642
hsa-mir-1231	-3.26942406	0.000645
hsa-mir-137	-3.253805991	1.18E-07
hsa-mir-4641	-3.25097157	0.001659
hsa-mir-346	-3.246582515	2.09E-12
hsa-mir-3938	-3.200758506	1.92E-05
hsa-mir-3919	-3.155012378	0.036603
hsa-mir-6734-5p	-3.111357101	3.34E-05
hsa-mir-4515	-3.087338154	0.000988
hsa-mir-4798-5p	-3.003297933	4.73E-05
hsa-mir-1296-5p	-2.998271356	3.55E-19
hsa-mir-499a-5p	-2.951335913	1.82E-22

hsa-mir-6877-3p	-2.918043324	0.009025
hsa-mir-4683	-2.897992887	0.088313
hsa-mir-889-3p	-2.887188603	6.96E-06
hsa-mir-548v	-2.884522164	0.00199
hsa-mir-6837-5p	-2.870282484	0.092431
hsa-mir-33a-5p	-2.85132041	1.56E-08
hsa-mir-33b-5p	-2.803411473	4.69E-05
hsa-mir-2276-5p	-2.803207542	0.006057
hsa-mir-3617-5p	-2.7736988	0.000129
hsa-mir-4640-3p	-2.732967145	4.14E-15
hsa-mir-4671-3p	-2.692064976	0.010309
hsa-mir-770-5p	-2.691991474	7.22E-05
hsa-mir-375	-2.661623896	0.003828
hsa-mir-5680	-2.627558688	4.3E-05
hsa-mir-29c-5p	-2.619462566	3.13E-05
hsa-mir-5187-5p	-2.593095627	0.013765
hsa-mir-767-3p	-2.588172216	0.086596
hsa-mir-767-5p	-2.561542889	5.46E-06
hsa-mir-1296-3p	-2.559424419	2.07E-06
hsa-mir-5699-3p	-2.553008641	0.000243
hsa-mir-499a-3p	-2.5337667	2.13E-05
hsa-mir-6865-3p	-2.530407423	0.003997
hsa-mir-154-3p	-2.52438009	3.5E-06
hsa-mir-4731-3p	-2.486035274	6.25E-05
hsa-mir-323b-3p	-2.469540812	0.000296
hsa-mir-23c-5p	-2.412188467	0.007408
hsa-mir-410-3p	-2.407545188	0.000337
hsa-mir-433-5p	-2.404262061	3.51E-05
hsa-mir-6786-3p	-2.384723392	0.000361
hsa-mir-3188	-2.356863582	0.006902
hsa-mir-3173-5p	-2.350174546	2.46E-05
hsa-mir-504-5p	-2.3490941	0.001939
hsa-mir-1252-5p	-2.321716355	0.004722
hsa-mir-6777-5p	-2.311100134	0.03001
hsa-mir-541-3p	-2.310545393	0.018361
hsa-mir-431-3p	-2.292609482	0.005421
hsa-mir-3617-3p	-2.292414732	0.043363
hsa-mir-4696	-2.276322168	0.042731
hsa-mir-383-5p	-2.268251448	0.021438
hsa-mir-181a-1-3p	-2.249843876	1.35E-07
hsa-mir-574-3p	-2.231871798	3.71E-08

hsa-mir-1287-3p	-2.230659437	0.015016
hsa-mir-4738-3p	-2.222032323	2.65E-05
hsa-mir-4639-3p	-2.205172397	0.088313
hsa-mir-128-1-5p	-2.184849244	2.4E-09
hsa-mir-4522	-2.161929403	0.086738
hsa-mir-668-3p	-2.161050845	0.001487
hsa-mir-33a-3p	-2.158204841	1.82E-05
hsa-mir-598-5p	-2.121227226	0.002472
hsa-mir-340-5p	-2.111971226	4.6E-15
hsa-mir-744-3p	-2.102262015	1.33E-17
hsa-mir-487b-5p	-2.095337846	0.000337
hsa-mir-496	-2.092772572	0.000729
hsa-mir-190a-3p	-2.078502972	2.45E-07
hsa-mir-3622b-5p	-2.052894994	0.07474
hsa-mir-1287-5p	-2.035257608	2.04E-07
hsa-mir-342-3p	-2.006392974	2.56E-07
hsa-mir-2467-5p	-1.970681033	2.95E-11
hsa-mir-5006-3p	-1.953153803	0.007238
hsa-mir-3117-3p	-1.951920261	7.86E-05
hsa-mir-6816-3p	-1.942709147	0.017795
hsa-mir-769-5p	-1.939223644	1.07E-09
hsa-mir-3200-5p	-1.938810033	1.14E-05
hsa-mir-1197	-1.937891982	0.005439
hsa-mir-889-5p	-1.934658854	0.013785
hsa-mir-5706	-1.923073585	0.00022
hsa-mir-6864-5p	-1.920186095	0.036325
hsa-mir-323a-3p	-1.918737409	0.008186
hsa-mir-4639-5p	-1.914133229	0.013894
hsa-mir-4524a-3p	-1.912735979	0.034391
hsa-mir-370-5p	-1.90491632	0.005587
hsa-mir-676-3p	-1.879756194	5.73E-05
hsa-mir-374a-5p	-1.876208259	2.78E-08
hsa-mir-23b-3p	-1.875196703	0.002026
hsa-mir-487a-3p	-1.870353599	0.005565
hsa-mir-6716-3p	-1.838309883	8.96E-06
hsa-mir-495-3p	-1.826598721	0.001327
hsa-mir-432-3p	-1.817771016	0.004092
hsa-mir-4786-5p	-1.816579544	0.00345
hsa-mir-4446-3p	-1.814624891	0.001304
hsa-mir-24-1-5p	-1.809976247	0.000136
hsa-mir-6854-5p	-1.795998563	0.041639

hsa-mir-6801-3p	-1.792177474	0.04266
hsa-mir-487b-3p	-1.773577578	0.005449
hsa-mir-3124-5p	-1.762217806	4.28E-05
hsa-mir-874-5p	-1.741697344	0.003599
hsa-mir-200a-5p	-1.739392198	0.036325
hsa-mir-127-3p	-1.730542796	0.000237
hsa-mir-656-3p	-1.725533881	0.001404
hsa-mir-340-3p	-1.710301433	1.13E-05
hsa-mir-425-5p	-1.707001652	0.021668
hsa-mir-6733-5p	-1.684821375	0.008351
hsa-mir-4767	-1.669098003	0.000322
hsa-mir-570-3p	-1.630876897	0.004528
hsa-mir-3622b-3p	-1.630019717	0.032081
hsa-let-7b-3p	-1.622922449	4.71E-05
hsa-mir-491-3p	-1.615357504	0.007136
hsa-mir-769-3p	-1.606787467	6.96E-06
hsa-mir-128-2-5p	-1.575449237	0.020267
hsa-mir-381-3p	-1.564133634	0.025578
hsa-mir-6513-5p	-1.5524301	0.004703
hsa-mir-508-3p	-1.54732057	0.028869
hsa-mir-34b-3p	-1.536658622	0.05301
hsa-mir-487a-5p	-1.533604134	0.016524
hsa-mir-539-3p	-1.524742692	0.021111
hsa-mir-382-3p	-1.501630213	0.012798
hsa-mir-1179	-1.460102871	0.000735
hsa-mir-374b-5p	-1.455315394	7.8E-05
hsa-mir-582-5p	-1.450128597	0.0015
hsa-mir-3157-3p	-1.437588285	0.008414
hsa-mir-4787-3p	-1.415243601	1.02E-06
hsa-mir-6516-5p	-1.410725012	0.036503
hsa-mir-30a-5p	-1.397896473	0.000842
hsa-mir-19b-1-5p	-1.393045504	0.044401
hsa-mir-190a-5p	-1.389059801	0.000338
hsa-mir-1243	-1.386412968	0.009165
hsa-mir-5000-3p	-1.367874101	0.006057
hsa-mir-323a-5p	-1.365155104	0.094132
hsa-mir-1307-5p	-1.353103997	0.005016
hsa-mir-26a-2-5p	-1.342686601	0.001579
hsa-mir-3064-3p	-1.332588047	0.026172
hsa-mir-2116-5p	-1.330017776	0.014271
hsa-let-7d-3p	-1.325634161	1.71E-08

hsa-mir-582-3p	-1.308913011	0.006669
hsa-mir-2355-5p	-1.304273301	0.005455
hsa-mir-3177-3p	-1.303889539	0.003188
hsa-mir-3605-5p	-1.296930828	2.67E-06
hsa-mir-2682-5p	-1.289446577	0.074934
hsa-mir-548aw	-1.289309098	0.03109
hsa-mir-1306-5p	-1.289099472	6.22E-06
hsa-mir-30e-3p	-1.282757412	8.23E-11
hsa-mir-411-5p	-1.281877882	0.03707
hsa-mir-127-5p	-1.271041985	0.062211
hsa-mir-98-5p	-1.254655455	4.31E-05
hsa-mir-2355-3p	-1.250224897	0.001181
hsa-mir-491-5p	-1.249652635	0.025213
hsa-mir-4757-3p	-1.23285736	0.063755
hsa-mir-132-3p	-1.220077107	0.004703
hsa-mir-760	-1.215528606	0.004148
hsa-mir-29b-2-5p	-1.20869022	0.003667
hsa-mir-30d-5p	-1.163269099	0.000121
hsa-mir-598-3p	-1.163215254	0.034804
hsa-mir-421	-1.157078383	0.000197
hsa-mir-2276-3p	-1.156498558	0.046465
hsa-mir-103a-2-5p	-1.155804686	0.005806
hsa-mir-136-3p	-1.145208129	0.043472
hsa-mir-377-5p	-1.144030054	0.07354
hsa-let-7g-3p	-1.114272501	0.002022
hsa-mir-3615	-1.091814269	0.000489
hsa-mir-101-1-5p	-1.08985508	0.007773
hsa-mir-1271-5p	-1.067338323	0.093646
hsa-mir-6882-5p	-1.061708395	0.040394
hsa-mir-30e-5p	-1.053859568	0.001815
hsa-mir-3157-5p	-1.052139957	0.050103
hsa-mir-379-3p	-1.02487354	0.098471
hsa-mir-548e-5p	-1.009342823	0.010383
hsa-mir-29c-3p	-1.006520589	0.05112
hsa-mir-887-3p	-0.993547218	0.005071
hsa-mir-374b-3p	-0.975164486	0.011259
hsa-mir-6847-5p	-0.9523956	0.088672
hsa-mir-377-3p	-0.94743454	0.069548
hsa-mir-1294	-0.920995434	0.061254
hsa-mir-5010-3p	-0.920692378	0.000154
hsa-mir-744-5p	-0.917332691	0.001091

hsa-mir-18a-5p	-0.911448427	0.040929
hsa-mir-151a-3p	-0.901481697	0.001041
hsa-mir-185-5p	-0.900677333	0.01874
hsa-mir-200a-3p	-0.894108186	0.0072
hsa-mir-590-3p	-0.8828646	0.009719
hsa-mir-191-5p	-0.877008257	0.032978
hsa-mir-628-5p	-0.871183517	0.009826
hsa-mir-1343-3p	-0.838614958	0.06286
hsa-mir-590-5p	-0.836899236	0.07241
hsa-mir-140-3p	-0.832746602	7.66E-07
hsa-mir-185-3p	-0.808968134	0.005955
hsa-mir-29a-5p	-0.800814179	0.079153
hsa-mir-361-5p	-0.79534152	0.003733
hsa-mir-301a-3p	-0.794135626	0.001585
hsa-mir-628-3p	-0.737117266	0.028928
hsa-mir-197-3p	-0.695531065	0.023074
hsa-mir-328-3p	-0.678189635	0.013872
hsa-mir-374a-3p	-0.600607387	0.057143

303 miRNAs upregulated in GBM

miRNAs	log2FoldChange	padj
hsa-mir-199a-1-3p	10.64434196	5.36E-13
hsa-mir-615-3p	9.010995011	8.52E-12
hsa-mir-891b	8.338166241	1.29E-07
hsa-mir-6503-5p	7.787404321	1.19E-10
hsa-mir-892b	7.563046072	3.26E-06
hsa-mir-10b-3p	7.490793441	1.87E-24
hsa-mir-892a	7.46119447	3.44E-12
hsa-mir-891a-5p	7.193847133	1.5E-23
hsa-mir-3614-5p	7.126210092	6.96E-06
hsa-mir-6503-3p	7.037387117	6.7E-18
hsa-mir-196a-2-3p	6.965720644	0.000124
hsa-mir-675-5p	6.938105039	6.05E-05
hsa-mir-888-5p	6.679125184	6.95E-13
hsa-mir-10a-3p	6.414455237	2.06E-10
hsa-mir-199b-5p	6.302721448	5.89E-18
hsa-mir-4466	6.278754683	0.000225
hsa-mir-182-3p	6.188031394	0.001039
hsa-mir-196a-2-5p	6.062396213	0.000847
hsa-mir-4772-3p	6.024460792	0.000525

hsa-mir-3591-5p	5.964815058	0.001162
hsa-mir-599	5.960017647	0.001441
hsa-mir-96-5p	5.941830619	8.56E-17
hsa-mir-4474-3p	5.86214989	0.000815
hsa-mir-3619-5p	5.784464207	0.000865
hsa-mir-892c-3p	5.777927088	0.000112
hsa-mir-1908-5p	5.727832272	5.65E-08
hsa-mir-4516	5.678844182	7.82E-06
hsa-mir-615-5p	5.536571677	0.002546
hsa-mir-23a-5p	5.479908709	4.72E-08
hsa-mir-888-3p	5.313469015	0.003979
hsa-mir-3937	5.311168658	0.00489
hsa-mir-5001-5p	5.269621673	0.003007
hsa-mir-144-5p	5.226401556	3.23E-35
hsa-mir-3679-5p	5.166979855	7.8E-05
hsa-mir-4492	5.163014958	0.000125
hsa-mir-4732-5p	5.131183186	0.000139
hsa-mir-4741	5.079748452	0.000153
hsa-mir-3913-1-5p	4.988908301	0.005729
hsa-mir-3154	4.973593475	0.007408
hsa-mir-4711-5p	4.873385416	0.011004
hsa-mir-449c-5p	4.84365578	7.61E-10
hsa-mir-6727-5p	4.82369116	0.009674
hsa-mir-199a-2-3p	4.78188066	0.001039
hsa-mir-4792	4.749834494	0.003258
hsa-mir-10b-5p	4.703317623	2.28E-09
hsa-mir-135a-1-3p	4.663486707	1.31E-07
hsa-mir-3150b-3p	4.604819601	0.000986
hsa-mir-10a-5p	4.586851821	4.06E-11
hsa-mir-875-5p	4.574541378	0.02003
hsa-mir-6501-5p	4.515318008	0.001879
hsa-mir-214-5p	4.515068612	2.52E-12
hsa-mir-224-5p	4.509014911	2.57E-35
hsa-mir-450b-3p	4.457671673	0.020733
hsa-mir-449b-5p	4.445686838	4.32E-05
hsa-mir-548ac	4.442071825	0.023328
hsa-mir-3152-5p	4.406824605	0.036612
hsa-mir-3940-5p	4.383428174	0.024559
hsa-mir-573	4.350267753	0.025848
hsa-mir-7641-1	4.345418817	0.04592
hsa-mir-196b-3p	4.315677427	0.000558

hsa-mir-21-5p	4.286838128	9.39E-68
hsa-mir-4649-5p	4.284220542	0.007136
hsa-mir-6125	4.271650171	0.002899
hsa-mir-144-3p	4.219337399	2.96E-16
hsa-mir-3920	4.109459812	0.044401
hsa-mir-451a	4.096527861	7.68E-13
hsa-mir-551b-5p	4.095224344	0.00018
hsa-mir-493-5p	4.057823668	2.08E-08
hsa-mir-135b-3p	4.012573246	2.9E-06
hsa-mir-1193	4.001081848	0.054796
hsa-mir-4433b-5p	3.959245353	0.000213
hsa-mir-6781-5p	3.950222004	0.07241
hsa-mir-3144-5p	3.923773142	0.078102
hsa-mir-4423-3p	3.897883408	0.000184
hsa-mir-493-3p	3.881828409	1.38E-09
hsa-mir-2115-5p	3.872872727	0.001596
hsa-mir-4772-5p	3.853879734	0.001908
hsa-mir-6720-3p	3.816643641	0.007899
hsa-mir-3652	3.797572585	0.077263
hsa-mir-6789-5p	3.790347197	0.074345
hsa-mir-4420	3.782966445	0.074849
hsa-mir-27a-5p	3.781444267	2.36E-09
hsa-mir-152-5p	3.775886002	0.001036
hsa-mir-214-3p	3.762880894	6.14E-10
hsa-mir-3606-5p	3.740836791	0.094132
hsa-mir-450b-5p	3.622671495	2.62E-14
hsa-mir-3651	3.585175099	0.000424
hsa-mir-4425	3.527773141	0.036325
hsa-mir-24-2-5p	3.512296126	5.44E-38
hsa-mir-3129-5p	3.511953024	0.001082
hsa-mir-3667-3p	3.501295082	0.036336
hsa-mir-3143	3.47687018	3.95E-06
hsa-mir-653-3p	3.475809892	2.77E-07
hsa-mir-4735-5p	3.468336361	0.063576
hsa-mir-150-5p	3.441809783	1.18E-07
hsa-mir-21-3p	3.384853023	1.6E-25
hsa-mir-216a-3p	3.384423666	0.008186
hsa-mir-92b-5p	3.374178997	1.24E-20
hsa-mir-222-5p	3.346011938	1.19E-10
hsa-mir-3151-5p	3.28726322	5.41E-05
hsa-mir-142-3p	3.263198797	5.5E-10

hsa-mir-6502-5p	3.253964929	0.000104
hsa-mir-653-5p	3.247047817	8.52E-08
hsa-mir-1245a	3.242946008	0.029596
hsa-mir-455-3p	3.234908476	2.16E-24
hsa-mir-581	3.233345217	0.099567
hsa-mir-503-5p	3.199446715	6.76E-12
hsa-mir-4517	3.174231533	0.024433
hsa-mir-155-5p	3.161263202	5.98E-26
hsa-mir-193a-3p	3.120519531	1.23E-05
hsa-mir-187-3p	3.099487298	0.000114
hsa-mir-9-1-5p	3.088030078	0.012714
hsa-mir-142-5p	3.083703236	3.14E-14
hsa-mir-6783-5p	3.070817773	0.069103
hsa-mir-4649-3p	3.05506392	5.85E-05
hsa-mir-449a	3.04773531	1.21E-06
hsa-mir-503-3p	3.046053501	0.003828
hsa-mir-497-3p	3.038173332	1.51E-05
hsa-mir-143-5p	3.020608046	2.99E-11
hsa-mir-210-3p	2.999022745	1.62E-13
hsa-mir-548ah-5p	2.997875771	5.59E-05
hsa-mir-486-2-5p	2.993217334	1.43E-05
hsa-mir-371b-5p	2.964888003	0.00038
hsa-mir-497-5p	2.946719999	3.58E-27
hsa-mir-219a-1-5p	2.941168853	1.31E-05
hsa-mir-7845-5p	2.930600707	0.025461
hsa-mir-296-3p	2.92688727	1.35E-07
hsa-mir-629-3p	2.925306851	6.7E-09
hsa-mir-210-5p	2.885724503	1.55E-19
hsa-mir-671-5p	2.879534696	6.8E-12
hsa-mir-424-3p	2.847405877	2.36E-12
hsa-mir-4648	2.842468096	0.001129
hsa-mir-4802-5p	2.805186086	0.07693
hsa-mir-431-5p	2.802158447	5.06E-05
hsa-mir-6813-5p	2.779422326	0.049002
hsa-mir-3125	2.749709627	0.003258
hsa-mir-224-3p	2.740316448	0.00021
hsa-mir-3648	2.732664667	0.009491
hsa-mir-3616-3p	2.694608606	0.050318
hsa-mir-190b	2.659951972	0.000729
hsa-mir-92b-3p	2.655957808	1.61E-08
hsa-mir-3681-5p	2.648658264	0.010004

hsa-mir-23a-3p	2.643320408	5.57E-23
hsa-mir-605-3p	2.639788781	0.00039
hsa-mir-145-3p	2.63313504	0.085883
hsa-mir-2115-3p	2.6268815	0.008701
hsa-mir-208b-3p	2.623669268	9.95E-11
hsa-mir-4521	2.609264921	0.001404
hsa-mir-3614-3p	2.602107505	0.085883
hsa-mir-551b-3p	2.5963246	2.09E-08
hsa-mir-195-3p	2.578475676	1.09E-11
hsa-mir-618	2.576638569	2.65E-09
hsa-mir-223-5p	2.574723893	7.34E-06
hsa-mir-3653-5p	2.572739801	0.002691
hsa-mir-4529-3p	2.545602948	0.082561
hsa-mir-2114-3p	2.52615738	0.004269
hsa-mir-4777	2.514942667	0.04251
hsa-mir-455-5p	2.507895919	9.53E-18
hsa-mir-188-5p	2.494303025	1.49E-13
hsa-mir-7705	2.485467174	8.84E-13
hsa-mir-34a-5p	2.48024137	2.5E-11
hsa-mir-489-3p	2.47817385	0.001413
hsa-mir-7976	2.465812395	0.00116
hsa-mir-1262	2.451160333	1.22E-08
hsa-mir-221-5p	2.422283786	2.64E-05
hsa-mir-217	2.390310949	0.012097
hsa-mir-335-5p	2.368474571	1.32E-05
hsa-mir-1228-5p	2.341541147	3.13E-05
hsa-mir-1228-3p	2.337993802	9.24E-12
hsa-mir-34a-3p	2.322649695	5.25E-09
hsa-mir-150-3p	2.320024718	0.001413
hsa-mir-3660	2.299181705	0.078343
hsa-mir-944	2.290271539	0.001009
hsa-mir-486-5p	2.253027222	0.001128
hsa-mir-452-5p	2.239374262	1.45E-06
hsa-mir-1276	2.230785921	0.000585
hsa-mir-675-3p	2.227597422	0.018361
hsa-mir-130a-3p	2.224897036	2.73E-19
hsa-mir-3194-5p	2.224266896	0.000168
hsa-mir-31-5p	2.213113642	0.001585
hsa-mir-3144-3p	2.212310666	0.09439
hsa-mir-4709-3p	2.184555208	0.084189
hsa-mir-365b-5p	2.180101164	0.000106

hsa-mir-4758-3p	2.174043188	0.013898
hsa-mir-3065-3p	2.164016919	0.001009
hsa-mir-500b-3p	2.148243706	0.015018
hsa-mir-2114-5p	2.118147291	0.039047
hsa-mir-148a-3p	2.095352716	0.002162
hsa-mir-542-5p	2.089303642	0.000397
hsa-mir-502-3p	2.085863982	6.54E-08
hsa-mir-1247-3p	2.083392861	0.004804
hsa-mir-130a-5p	2.076211659	4.15E-05
hsa-mir-16-2-5p	2.075702911	0.046842
hsa-mir-25-5p	2.07241588	3.43E-08
hsa-mir-135b-5p	2.071163782	0.001585
hsa-mir-3129-3p	2.06014871	0.000147
hsa-mir-3687	2.043425119	0.017386
hsa-mir-3659	2.018908799	0.034391
hsa-mir-193a-5p	2.000240214	0.000122
hsa-mir-335-3p	1.969685982	0.006313
hsa-mir-27a-3p	1.943334761	6.75E-08
hsa-mir-4443	1.872535318	0.063487
hsa-mir-376a-2-5p	1.87016591	0.036421
hsa-mir-125b-1-5p	1.853185987	0.037788
hsa-mir-15b-3p	1.845616161	3.75E-05
hsa-mir-3940-3p	1.841297504	0.001408
hsa-mir-215-5p	1.83030077	0.005729
hsa-mir-3194-3p	1.826913167	0.07403
hsa-mir-4803	1.806016361	0.014746
hsa-mir-483-5p	1.790356845	0.052319
hsa-mir-223-3p	1.781544573	0.000182
hsa-mir-18b-3p	1.76742456	0.058868
hsa-mir-4709-5p	1.7469835	0.049839
hsa-mir-3176	1.734054519	2.4E-07
hsa-mir-6852-5p	1.723758637	0.001401
hsa-mir-4690-3p	1.705674515	0.085883
hsa-mir-4687-3p	1.705132464	0.078102
hsa-mir-146b-3p	1.688348474	2.06E-05
hsa-let-7c-3p	1.687789091	4.36E-10
hsa-mir-339-5p	1.686605981	0.004379
hsa-mir-494-3p	1.684042774	0.011388
hsa-mir-1248	1.680062251	0.083343
hsa-mir-3922-3p	1.670103933	0.000676
hsa-mir-3607-3p	1.665333214	0.00036

hsa-mir-532-3p	1.655178084	2.63E-07
hsa-mir-542-3p	1.640358045	0.001794
hsa-mir-222-3p	1.637117311	0.000468
hsa-mir-1247-5p	1.620314816	0.034315
hsa-mir-3942-5p	1.613380678	0.044401
hsa-mir-452-3p	1.609705392	0.003298
hsa-mir-2277-5p	1.58357003	2.66E-13
hsa-mir-5002-5p	1.544243344	0.004841
hsa-mir-548al	1.522041209	0.003396
hsa-mir-548ba	1.521477688	0.025848
hsa-mir-3622a-3p	1.513949174	0.014239
hsa-mir-488-5p	1.496687639	0.04027
hsa-mir-942-5p	1.477391243	0.001145
hsa-mir-548e-3p	1.475200257	0.001457
hsa-mir-31-3p	1.471610377	0.063755
hsa-mir-148a-5p	1.450328071	0.04555
hsa-mir-5091	1.440495483	0.062329
hsa-mir-3661	1.394569619	0.00017
hsa-mir-3065-5p	1.374175461	0.0503
hsa-mir-337-3p	1.367780501	0.031006
hsa-mir-221-3p	1.356747064	0.004711
hsa-mir-106b-3p	1.319560047	1.03E-07
hsa-mir-4775	1.285673282	1.11E-05
hsa-mir-877-5p	1.284517571	5.42E-08
hsa-mir-195-5p	1.284238258	1.52E-05
hsa-mir-28-5p	1.27851179	0.000435
hsa-mir-362-3p	1.261121222	0.007136
hsa-mir-152-3p	1.260255395	0.003994
hsa-mir-1468-5p	1.251278681	0.009171
hsa-mir-423-3p	1.244585844	0.000319
hsa-mir-502-5p	1.242368493	0.008283
hsa-mir-15b-5p	1.238590423	0.00427
hsa-mir-30d-3p	1.237730301	1.9E-06
hsa-mir-193b-5p	1.23168067	0.000865
hsa-mir-532-5p	1.228978092	2.6E-05
hsa-mir-4326	1.222525482	0.028057
hsa-mir-652-5p	1.215158205	0.007342
hsa-mir-589-3p	1.211485868	0.008804
hsa-mir-126-3p	1.187123192	8.96E-06
hsa-mir-501-3p	1.175903225	0.082561
hsa-mir-125a-3p	1.152142926	0.000538

hsa-mir-363-3p	1.143863016	0.000231
hsa-mir-92a-1-5p	1.139003545	0.06023
hsa-mir-660-3p	1.131528943	0.010643
hsa-mir-145-5p	1.112050845	0.00069
hsa-mir-671-3p	1.079562258	0.003772
hsa-mir-296-5p	1.057528607	0.021065
hsa-mir-28-3p	1.030273339	2.95E-05
hsa-mir-22-5p	1.028234596	0.003795
hsa-mir-93-3p	1.020870424	0.055331
hsa-mir-20b-5p	1.017728002	0.002664
hsa-mir-3691-5p	1.015500511	0.086964
hsa-mir-30b-3p	1.007876989	0.058235
hsa-mir-708-5p	1.003105777	0.028869
hsa-mir-548b-3p	0.989668069	0.045466
hsa-mir-29b-1-5p	0.979171562	0.018663
hsa-mir-365a-5p	0.97864232	0.063487
hsa-let-7c-5p	0.969665784	0.000969
hsa-mir-500a-3p	0.96851741	0.000676
hsa-mir-22-3p	0.954830527	0.000112
hsa-mir-320a	0.946964376	1.49E-05
hsa-mir-423-5p	0.939106154	0.001088
hsa-mir-339-3p	0.939050496	1.28E-05
hsa-mir-4645-3p	0.894507086	0.028869
hsa-mir-708-3p	0.88225033	0.059794
hsa-mir-186-3p	0.878073991	0.08693
hsa-mir-106a-5p	0.875773005	0.013765
hsa-mir-200b-3p	0.871480659	0.035436
hsa-mir-3187-3p	0.867203895	0.092431
hsa-mir-424-5p	0.856745368	0.056305
hsa-mir-664a-5p	0.843250825	0.001121
hsa-mir-25-3p	0.800612988	7.28E-05
hsa-mir-627-5p	0.767598621	0.063697
hsa-mir-93-5p	0.751179377	0.000194
hsa-mir-342-5p	0.720584364	0.025213
hsa-mir-501-5p	0.717129359	0.030507
hsa-mir-99a-3p	0.645931205	0.037788
hsa-mir-454-3p	0.636579146	0.005984
hsa-mir-106b-5p	0.615864603	0.061155

Supplemental Table S7: Delta-Ct values of hypoedited miRNAs in GBM which are downregulated when compared to both FC and CC samples

hsa-miR-889		hsa-miR-598		hsa-miR-377		hsa-miR-411	
889_CC	889_GBM	598_CC	598_GBM	377_CC	377_GBM	411_CC	411_GBM
-5.84	-6.7	-4.26	-4.83	-6.64	-7.50	-5.17	-6.17
-5.49	-6.79	-4.60	-5.48	-7.01	-8.46	-5.15	-4.91
-6.77	-8.66	-4.32	-5.18	-8.50	-8.28	-4.92	-5.83
-5.23	-7.30	-3.10	-6.16	-7.07	-9.46	-3.66	-4.82
-6.08	-8.91	-4.2	-5.90	-7.11	-9.31	-4.33	-7.16

Supplemental Table S8: C-to-U hypoedited miRNAs in Glioblastoma Multiforme (GBM)

					Target Prediction	
miRNAs	aPresence in samples	bMedian editing (in %)	Seed editing event	Position in precursor	Before/After editing	cOverlap (%)
hsa-mir-29a-3p	4/5/0	0.17/0.2/0	Yes	48	851/164	11 (1.29)
hsa-mir-23b-3p	0/3/0	0/0.51/0	Yes	60	838/1150	152 (18.14)
hsa-mir-100-5p	6/6/3	1.20/1.06/0.12	No	25	NA	NA
hsa-mir-99a-5p	6/6/5	2.63/4.05/1	No	25	NA	NA
hsa-mir-125a-5p	5/0/0	0.19/0/0	No	25	NA	NA
hsa-mir-374b-5p	4/5/0	0.32/0.27/0	No	22	NA	NA
hsa-mir-33a-5p	4/4/0	1.15/0.97/0	No	20	NA	NA
hsa-mir-204-5p	1/5/0	-/0.2/0	No	43	NA	NA
hsa-mir-99b-5p	2/3/0	-/0.78/0	No	19	NA	NA
hsa-mir-219a-2-5p	1/3/0	-/0.27/0	No	33	NA	NA

10 miRNAs were found to be significantly hypoedited in GBM (wilcoxon-two-tailed, $p<0.05$). All the events reported are novel hypoediting events.

a: Presence in samples (FC/CC/GBM)

b: Median editing (FC/CC/GBM)

c: Percentage overlap was calculated by overlapped targets/targets before editing

NA, not applicable because they were not seed editing events

”-“ indicates miRNAs where median value could not be calculated
